# Phytochemical Characterization and Cytotoxic Potential of the Ethyl Acetate Fraction of *Schima superba* Bark: An *In Vitro* and *In Silico* Investigation

**DOI:** 10.3390/molecules31142550

**Published:** 2026-07-22

**Authors:** Hieu Phu Chi Truong, Hong Khuyen Thi Pham, Thuy Mi Pham Lam, Tuan Anh Le, Van Ngo Thai Bich, Phu Tran Vinh Pham, Tan Khanh Nguyen, Kim Lien Thi Giang, Manh Hung Tran

**Affiliations:** 1 Faculty of Pharmacy, School of Medicine and Pharmacy, The University of Danang, Danang City 550000, Vietnam; tpchieu@smp.udn.vn (H.P.C.T.); phamthihongkhuyendtht123@gmail.com (H.K.T.P.); 2 Faculty of Physics and Chemistry, University of Education, The University of Danang, Danang City 550000, Vietnam; 3144122013@ued.udn.vn; 3 Mientrung Institute for Scientific Research, Vietnam National Museum of Nature, Vietnam Academy of Science and Technology (VAST), Hue City 530000, Vietnam; ltanh@misr.vast.vn; 4 Faculty of Chemical Engineering, University of Science and Technology, The University of Danang, Danang City 550000, Vietnam; ntbvan@dut.udn.vn; 5 Biomedical Science Department, VN-UK Institute for Research and Executive Education, The University of Danang, Danang City 550000, Vietnam; phu.pham@vnuk.udn.vn (P.T.V.P.); lien.giang@vnuk.edu.vn (K.L.T.G.); 6 Institute for Biocomputation and Physics of Complex Systems, University of Zaragoza, 50018 Zaragoza, Spain; khanhnt@donga.edu.vn

**Keywords:** *Schima superba*, stem bark, human cancer cells, in vitro, in silico

## Abstract

Natural products represent a valuable source of anticancer agents, although their mechanisms of action are often incompletely understood. In this study, we evaluated the cytotoxic effects of the ethyl acetate (EA) fraction derived from *Schima superba* bark. The EA fraction exhibited selective cytotoxicity against HepG2 and MCF-7 cancer cells, with minimal effects on normal HEK293 cells, and induced apoptosis as evidenced by time- and dose-dependent activation of caspase-3. Phytochemical profiling by UPLC-QTOF-MS/MS identified 18 major constituents, predominantly phenylethanoid glycosides and triterpenoids. To gain insight into the underlying mechanism, molecular docking, 200 ns molecular dynamics simulations, and MM-PBSA analyses were performed targeting poly (ADP-ribose) polymerase 1 (PARP1) and caspase-3. Among the identified compounds, cistanoside D displayed favorable binding affinity toward both targets, but formed a more stable and energetically favorable complex with PARP1 during molecular dynamics simulations, whereas its interaction with caspase-3 was comparatively weak. These findings suggest that cistanoside D may preferentially interact with PARP1, while the observed activation of caspase-3 is likely associated with downstream apoptotic processes rather than direct enzymatic modulation. This study provides an integrated evaluation of the phytochemical composition and anticancer potential of *Schima superba* bark extract, and identifies cistanoside D as a promising candidate for further investigation. These results contribute to a better understanding of the molecular basis underlying the bioactivity of this medicinal plant and support its potential as a source of anticancer agents.

## 1. Introduction

Cancer ranks as the second leading cause of death worldwide, following cardiovascular diseases, and has emerged as a major barrier to increasing life expectancy in the 21st century [1]. According to the International Agency for Research on Cancer (IARC) via the GLOBOCAN database, an estimated 19.3 million new cancer cases and nearly 10 million cancer-related deaths were reported globally in 2020 alone [2]. The epidemiological transition from infectious diseases to non-communicable diseases, coupled with population aging and increasing exposure to environmental risk factors, has significantly accelerated the global cancer burden, particularly in developing countries [3].

Despite significant advances in chemotherapy, the emergence of drug resistance and severe side effects continue to limit treatment efficacy, highlighting the urgent need for novel therapeutic agents with improved safety and effectiveness. In response to this growing challenge, the discovery of natural compounds capable of selectively inducing cytotoxicity through apoptosis has become a key strategy in modern pharmacology for the development of next-generation anticancer therapies [4,5]. Natural products have long served as a rich source of bioactive compounds for drug discovery, particularly in oncology. Numerous plant-derived secondary metabolites, including flavonoids, alkaloids, and triterpenoids, have demonstrated potent anticancer activities through diverse mechanisms such as induction of apoptosis, inhibition of cell proliferation, and modulation of key signaling pathways. In this context, medicinal plants used in traditional medicine represent a valuable reservoir for identifying new lead compounds [6,7].

The genus *Schima* (Theaceae) comprises several evergreen tree species widely distributed in tropical and subtropical regions of Asia [8,9]. Among them, *Schima superba* Gardn. et Champ.; commonly known in Vietnamese as “Vối răng cưa,” has attracted increasing attention due to its pharmacological potential [10]. Traditionally, various parts of this plant have been used in folk medicine for the treatment of inflammatory conditions, detoxification, and pain relief. Phytochemical investigations have revealed that *S. superba* is rich in bioactive secondary metabolites, particularly triterpenoid saponins, flavonoids, and polyphenolic compounds [11,12,13]. Among the isolates, kaempferol 3-O-rhamnoside has been shown to exert neuroprotective effects through modulation of calcium-dependent signaling pathways. In addition, extracts from *S. superba* bark have demonstrated significant antifungal activity against *Candida albicans*, with sasanquasaponin III identified as a major active constituent [11]. Furthermore, several novel oleanane-type triterpenoid saponins had been isolated from the roots of this plant, some of which exhibited notable cytotoxic activity against melanoma cell lines [12].

Despite these promising findings, the anticancer potential of *S. superba*, particularly against leukemia cells, remains insufficiently explored. To date, there is a lack of systematic studies evaluating the cytotoxic effects of its solvent fractions, as well as limited understanding of the underlying molecular interactions responsible for its biological activity. In particular, the integration of experimental and computational approaches to elucidate the mechanism of action of *S. superba* derived compounds has not been fully addressed. Therefore, the present study aimed to characterize the phytochemical composition of the ethyl acetate fraction obtained from *S. superba* bark and evaluate its cytotoxic activity against human cancer cell lines. In addition, apoptosis-related assays and computational approaches were employed to explore potential mechanisms associated with the observed biological activity. We hypothesized that bioactive constituents present in the ethyl acetate fraction contribute to the cytotoxic effects and may interact with molecular targets involved in cancer cell survival and apoptosis.

## 2. Results

### 2.1. Cytotoxic Activity Results

The cytotoxic effects of different fractions obtained from the bark of *S. superba* were evaluated against three human cancer cell lines (A549, HepG2, and MCF-7) and a normal human embryonic kidney cell line (HEK293) (Table 1). Among the fractions, the ethyl acetate fraction showed the lowest IC_50_ values across the tested cancer cell lines, with IC_50_ values of 27.98 ± 1.63 µg/mL for HepG2, 29.46 ± 0.87 µg/mL for MCF-7, and 45.30 ± 2.31 µg/mL for A549 cells. In comparison, the dichloromethane fraction demonstrated moderate activity, with IC_50_ values ranging from 57.82 ± 2.12 to 70.10 ± 2.71 µg/mL. The *n*-hexane fraction exhibited lower activity, particularly against A549 cells (IC_50_ > 100 µg/mL), while its effects on HepG2 and MCF-7 cells remained within a higher IC_50_ range (67.87 ± 3.15 and 76.37 ± 3.04 µg/mL, respectively). The methanol extract showed moderate cytotoxicity, with IC_50_ values of 36.86 ± 1.82 µg/mL for HepG2, 48.05 ± 2.09 µg/mL for MCF-7, and 75.36 ± 1.89 µg/mL for A549 cells.

A consistent pattern was observed in which HepG2 and MCF-7 cells were more sensitive to the tested fractions compared to A549 cells. This trend was evident across most fractions, suggesting cell line-dependent differences in susceptibility. In contrast, none of the fractions exhibited notable cytotoxic effects on HEK293 cells, with IC_50_ values exceeding 300 µg/mL in all cases, indicating a lack of detectable toxicity toward normal cells under the tested conditions. Ellipticine was included as a positive control and displayed substantially lower IC_50_ values in the submicromolar range (0.32–0.40 µM), confirming the responsiveness of the assay system. Although the fractions showed higher IC_50_ values compared to the reference compound, the observed activities, particularly for the ethyl acetate fraction, fall within a range commonly reported for bioactive plant-derived extracts. These results suggest that cytotoxic constituents of *S. superba* bark are more likely to be concentrated in the ethyl acetate fraction, while non-polar fractions contribute less to the observed activity. The absence of cytotoxicity toward HEK293 cells further indicates a degree of selectivity of the tested fractions toward cancer cell lines.

### 2.2. Caspase 3 Activation Analysis

**Figure 1:**
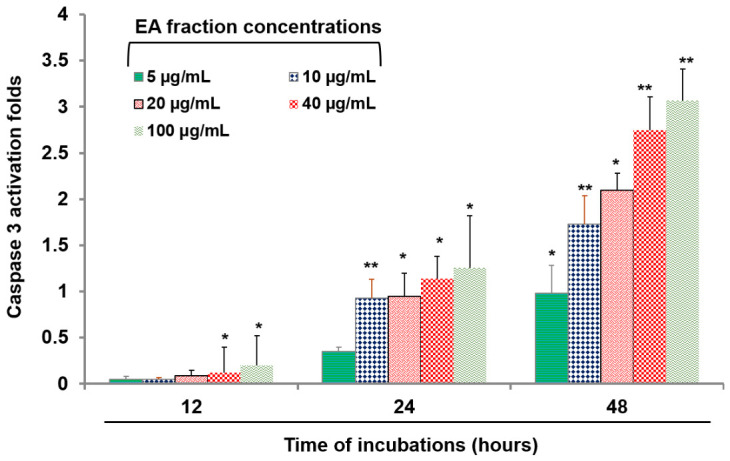
Caspase-3 activation in HepG2 cells. HepG2 cells were treated with EA fractions (10, 20, and 40 μg/mL) for 12–48 h. The control group was treated with 1% DMSO. Data are expressed as the mean ± standard deviation (SD) of three independent experiments (* *p* < 0.01; ** *p* < 0.05).

Based on its lowest IC_50_ values against HepG2 cells among the tested fractions, the ethyl acetate fraction (EA) was selected for further evaluation of its apoptotic effect via caspase-3 activation in HepG2 cell line. Caspase-3 activation in HepG2 cells following treatment with the EA fraction was assessed at concentrations ranging from 5 to 100 µg/mL over incubation periods of 12, 24, and 48 h (Figure 1). The results demonstrate a progressive increase in caspase-3 activity that depends on both treatment duration and concentration. At 12 h, caspase-3 activation remained at a low level across all tested concentrations, with only minor increases observed at 20–100 µg/mL, not exceeding approximately 0.2–0.3-fold relative to the control. By 24 h, a more pronounced elevation in caspase-3 activity was detected. Cells treated with 5 µg/mL showed a modest increase (~0.3-fold), whereas higher concentrations (10–100 µg/mL) resulted in activation levels ranging from approximately 0.9- to 1.2-fold. Several of these increases reached statistical significance, indicating the initiation of apoptotic signaling. At 48 h, caspase-3 activation increased further across all concentrations. The activity reached approximately 1.0-fold at 5 µg/mL and progressively increased to around 3.0-fold at 100 µg/mL. Notably, significant differences were observed at concentrations of 10 µg/mL and above, with the highest activation levels recorded at 40 and 100 µg/mL. The data indicated that the EA fraction induced caspase-3 activation in HepG2 cells in a time- and dose-dependent manner. The gradual increase in caspase-3 activity, particularly at later time points, is consistent with the involvement of apoptotic mechanisms in the cytotoxic effects observed for this fraction.

### 2.3. UPLC-QTOF-MS/MS Analysis Results

Comprehensive phytochemical profiling of the EA fraction was performed using UPLC–QTOF–MS/MS. The equations were based on the excimer [M + H]+ with a mass error tolerance of 10 ppm and partial isotopic abundance. The conducted searches in chemical databases, including ChemSpider (https://www.chemspider.com/ (accessed on 21 April 2026)) and MassBank (https://www.massbank.jp/MassBank/ (accessed on 21 April 2026)). A total of 18 compounds were detected, among which several were structurally assigned based on their molecular formulas and fragmentation patterns, while some peaks remained unidentified. The identified compounds were mainly classified into phenolic compounds, terpenoids (including diterpenoids and triterpenoids), steroids, coumarins, anthranoids, and other nitrogen- or glycoside-containing constituents (Table 2 and Figure S1 in Supporting Information). Among these, phenolic compounds represented a major group, including 1,7-diphenyl-5-hydroxy-4,6-heptadien-3-one, 2,4,7-trimethoxyphenanthrene, cistanoside D, flavokawain B, and hispidin. These compounds are commonly associated with redox-modulating properties and may contribute to the observed biological activity. In addition to phenolics, some terpenoid-derived compounds were identified, including 19-acetoxy-9(11),15-pimaradiene (diterpenoid), phytolaccagenin, toosendanin, and yemuoside YM6 (triterpenoids), as well as yadanzioside A (terpenoid). The presence of these compounds suggests that the EA fraction contains structurally complex secondary metabolites that may be involved in cytotoxic mechanisms, as terpenoids are frequently reported to exhibit anticancer activity through apoptosis-related pathways.

Notably, steroidal glycosides, such as periplocoside C and periplocoside M, were also detected. These compounds have been previously associated with membrane-disrupting effects and modulation of cell survival pathways. The detection of coumarin derivatives (e.g., scopoletin derivative) and anthranoid compounds further indicates the chemical diversity of the EA fraction, as these classes are known to possess various pharmacological activities, including cytotoxic and pro-apoptotic effects. Several compounds classified nitrogen-containing molecules (2,6-dimethylaniline, eleutherazine B) and glycosides (glucosinalbin, nuezhenidic acid), were also identified, suggesting the presence of additional bioactive constituents that may contribute synergistically to the overall activity of the fraction. It is noteworthy that a number of detected peaks could not be assigned to known structures, indicating that the EA fraction may contain previously uncharacterized or less-reported compounds. This highlights the chemical complexity of *S. superba* bark and suggests the need for further isolation and structural elucidation studies. Notably, the LC/MS profiling reveals that the ethyl acetate fraction is enriched in semi-polar bioactive compounds, particularly phenolics and terpenoids, which may underlie the cytotoxic and apoptosis-inducing effects observed in HepG2 cells.

### 2.4. Docking Simulation Analysis

#### 2.4.1. Binding Modes and Key Interactions with Caspase-3

**Figure 2:**
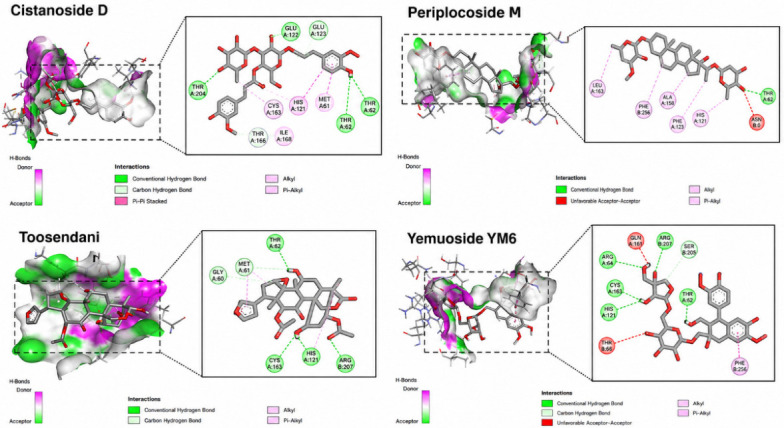
Molecular docking analysis of potential bioactive compounds within the binding pocket of Caspase-3 (PDB ID: 2XYP). Left panels: 3D visualization of the ligands within the solvent-accessible surface of the catalytic site. Right panels: 2D interaction diagrams illustrating specific amino acid residues involved in hydrogen bonding (green), hydrophobic contacts (pink/purple), and unfavorable interactions (red).

To further elucidate the molecular mechanism underlying the pro-apoptotic activity of the isolated compounds, molecular docking simulations of all identified compounds were performed within the catalytic site of caspase-3 (PDB ID: 2XYP) (see Supporting Information file). The targeted binding pocket was defined by a constellation of critical residues from both Chain A and Chain B, including His61, Arg121, Ser122, Gln123, Trp128, Cys163, Ser166, Asn204, and Trp256 (Figure 2 and Table 3). The computational results demonstrated that the candidate ligands effectively occupied the enzymatic cleft, establishing diverse interaction networks with the catalytic residues. Among these, cistanoside D exhibited a robust binding affinity characterized by a dense hydrogen-bonding network with His61 and Gly122. The stability of the complex was further augmented by pi-pi T-shaped and pi-alkyl interactions with Met61 and Cys163, suggesting a strong anchoring within the active site. Toosendanin demonstrated a highly favorable binding orientation, forming direct hydrogen bonds with Thr62, Cys163, and Arg207. Notably, the interaction with Cys163, a key residue of the Caspase-3 catalytic triad, indicates that Toosendanin may directly modulate the enzyme’s proteolytic function. In addition, yemuoside YM6 established a complex multifaceted interaction profile, involving hydrogen bonds with Arg64, Asn204, Ser121, and Arg207. Its aromatic scaffold was further stabilized by a pi-pi T-shaped interaction with Phe256, although minor unfavorable acceptor-acceptor overlaps were noted. Meanwhile, periplocoside M utilized its steroidal framework to form extensive hydrophobic contacts, including alkyl and pi-alkyl interactions with Phe128, Cys163, and His121.

#### 2.4.2. Binding Modes and Key Interactions with PARP1

**Figure 3:**
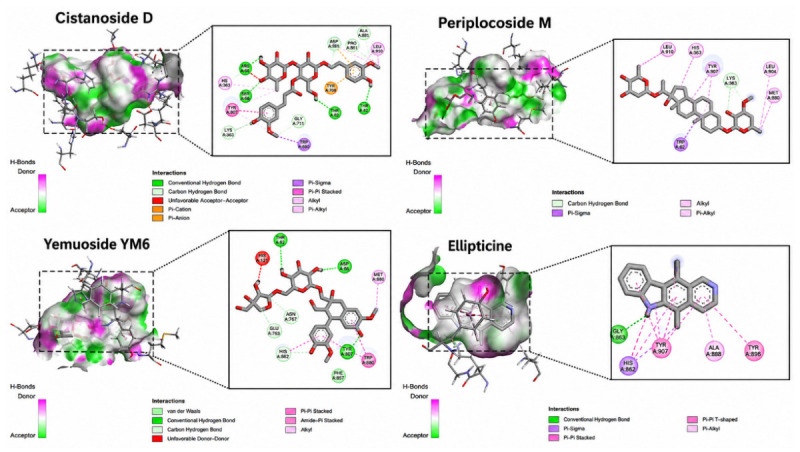
Molecular docking models of selected compounds within the catalytic pocket of PARP1 (PDB ID: 7KK4). Left panels illustrate the solvent-accessible surface of the binding pocket colored by hydrophobicity/hydrogen-bonding potential; right panels represent detailed 2D ligand-residue interaction diagrams.

The computational analysis revealed that the three selected compounds as cistanoside D, periplocoside M, and yemuoside YM6 effectively occupied the catalytic cleft, establishing interaction networks comparable to those of the reference inhibitor ellipticine (Figure 3 and Table 4). Among them, cistanoside D demonstrated high structural stability within the active site, primarily mediated by a robust hydrogen-bonding network. Key conventional hydrogen bonds were observed with Arg881 and Ser894, which are critical for anchoring inhibitors within the PARP1 catalytic domain. Additionally, pi-alkyl interactions with Ala880 further stabilized the phenylpropanoid glycoside scaffold. Despite its steroidal framework, periplocoside M exhibited favorable binding through extensive hydrophobic contacts. It established significant alkyl and pi-alkyl interactions with Leu713 and Ala898, effectively filling the hydrophobic cavity adjacent to the catalytic center. Analysis of yemuoside YM6 revealed a multifaceted binding affinity. Notably, it formed a crucial hydrogen bond with Glu988, a residue essential for the enzymatic activity of PARP1. The binding was further reinforced by pi-pi stacked interactions with the aromatic ring of Phe897, enhancing the overall complex stability. Meanwhile, the planar structure of Ellipticine allowed for deep penetration into the narrow binding cleft. It established a conventional hydrogen bond with Gly863 and was stabilized by pi-pi T-shaped and pi-alkyl interactions with His862, Tyr907, and Ala898. The docking data indicate that the isolated compounds target the critical catalytic residues of PARP1, particularly Arg881 and Glu988. These interactions are consistent with the established mechanism of PARP1 inhibitors, which function by preventing DNA repair in malignant cells. These molecular insights provide a theoretical basis for the cytotoxic activity observed in the experimental assays against the cancer cells, suggesting that PARP1 inhibition may be a contributing factor to the induced apoptosis.

#### 2.4.3. Physicochemical Properties and Drug-likeness Assessment

To evaluate the pharmacokinetic potential of the bioactive compounds, a comprehensive in silico ADME (Absorption, Distribution, Metabolism, and Excretion) profile was generated using the SwissADME platform (http://www.swissadme.ch) (Table 5). The analysis revealed that ellipticine, the reference control, strictly adhered to Lipinski’s criteria with zero violations, characterized by a low molecular weight (246.31 g/mol) and high lipophilicity (Log P = 3.92), suggesting excellent passive membrane diffusion. Among the natural constituents, periplocoside M emerged as the most promising drug-like candidate, exhibiting only a single violation regarding its molecular mass (>500 Da). Its balanced lipophilicity (Log P = 3.40) indicates a favorable equilibrium between aqueous solubility and lipid permeability. In contrast, cistanoside D and yemuoside YM6 showed significant deviations from the Ro5 with three violations each, primarily due to their elevated molecular weights (>650 g/mol) and an excessive number of hydrogen bond donors (nHBD) and acceptors (nHBA). These characteristics, coupled with high Total Polar Surface Area (TPSA) values exceeding 140AA, suggest limited passive gastrointestinal absorption and low blood–brain barrier (BBB) penetrability. Aqueous solubility Log S is a critical determinant for oral drug absorption. Yemuoside YM6 displayed the highest solubility (−2.31), whereas periplocoside M and ellipticine exhibited lower solubility profiles (−5.63 and −5.05, respectively), indicating a more lipophilic nature. Furthermore, the molecular flexibility, represented by the number of rotatable bonds (nRotB), was notably higher in cistanoside D and yemuoside YM6, which may facilitate conformational adjustments during protein-ligand binding despite the potential entropy penalties.

#### 2.4.4. Pharmacokinetic Profiles and In Silico Toxicity Predictions

The ADME and toxicity profiles of the selected compounds were further elucidated using SwissADME and ProTox 3.0 platforms to evaluate their clinical viability (Table 6 and Table 7). According to the SwissADME predictions, periplocoside M and ellipticine displayed high gastrointestinal (GI) absorption, whereas cistanoside D, toosendanin, and yemuoside YM6 exhibited low absorption profiles, likely due to their complex glycosidic structures and high molecular weights. Notably, only ellipticine was predicted to cross the blood–brain barrier (BBB), suggesting that the natural compounds may have minimal central nervous system side effects. Regarding metabolism, the natural constituents showed a favorable profile with negligible inhibition of major Cytochrome P450 isoforms (CYP1A2, 2C19, 2C9, and 3A4), potentially reducing the risk of drug–drug interactions compared to the multifaceted inhibition observed with ellipticine. In silico toxicity assessment via ProTox 3.0 classified the compounds into different toxicity classes based on their predicted LD50 values. Cistanoside D was identified as the least toxic agent (LD = 5000 mg/kg, Class V), while yemuoside YM6 and periplocoside M were categorized as Class IV (harmful). Toosendanin exhibited the highest acute toxicity risk (LD50 = 244 mg/kg, Class III). These findings also suggest that cistanoside D and periplocoside M represent safer lead candidates for further drug development, balancing potent bioactivity with favorable safety margins.

### 2.5. Molecular Dynamic Actions

Among the potential compounds present in the EA extract, we selected cistanoside D for further investigation using molecular dynamics simulation. The results of the molecular dynamics simulation showed that the protein-ligand system achieved a relatively stable structural state throughout the 200 ns simulation.

**Figure 4:**
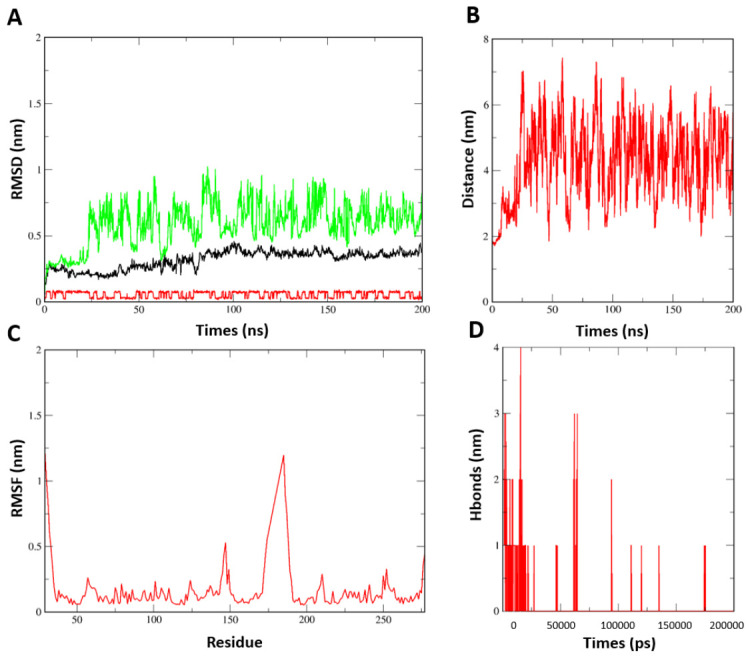
MD simulation results between ligand cistanoside D and caspase-3 over 200 ns. (**A**): RMSD of ligand and caspase-3 (black: protein backbone, red: ligand, and green: complex); (**B**): Distance between ligand and protein; (**C**): RMSF; (**D**): Number of hydrogen bonds between ligand and protein.

For caspase-3, the interaction profile suggested unstable binding, as the compound moved away from the binding site after approximately 20 ns (Figure 4). This observation was consistent with the increased fluctuation in complex RMSD after this time point and the limited hydrogen-bond formation between the compound and protein during the simulation. These results suggest that cistanoside D may not strongly or directly interact to caspase-3, but may instead influence apoptosis through indirect mechanisms.

**Figure 5:**
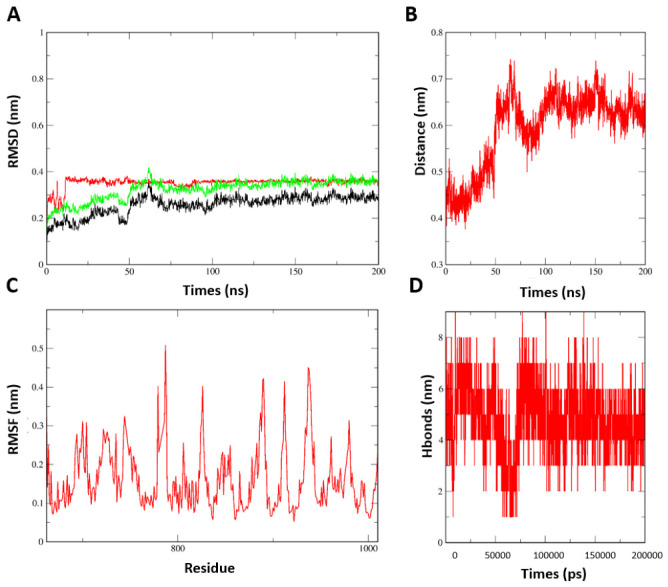
MD simulation results between cistanoside D and PARP1 over 200 ns (**A**). RMSD of ligand and PARP1 (black: protein backbone, red: ligand, and green: complex); (**B**): Distance between ligand and protein; (**C**): RMSF; (**D**): Number of hydrogen bonds between ligand and protein.

Next, we performed molecular dynamics (MD) simulation of the complex between cistanoside D and PARP1 (Figure 5). The results showed that the complex achieved high structural stability and maintained stable interactions throughout the 200 ns simulation, indicating the significant inhibitory potential of this compound toward the target protein. RMSD analysis (Figure 5A) revealed that the backbone structure of PARP1 remained stable within the range of ~0.15–0.30 nm and rapidly reached equilibrium after approximately 30–40 ns, suggesting that the protein did not undergo significant structural deformation during interaction with the ligand. Notably, the RMSD of the ligand remained stable around ~0.30–0.38 nm without sudden fluctuations, reflecting the good accommodation of cistanoside D within the binding pocket. The RMSD of the complex (~0.25–0.38 nm) also remained stable, indicating that the protein-ligand system reached a reliable dynamic equilibrium state. The distance between the ligand and the protein (Figure 5B) fluctuated within a narrow range (~0.4–0.7 nm) and remained relatively stable after the initial phase of the simulation. This indicates that cistanoside D was well retained within the binding region of PARP1 and did not dissociate from the active site, in clear contrast to the caspase-3 system described previously. RMSF analysis (Figure 5C) showed that most residues of PARP1 exhibited low flexibility (<0.3 nm), with only a few loop regions showing higher fluctuations, which is a typical characteristic of protein structures. Importantly, no significant increase in fluctuations was observed in the active site, indicating that ligand binding did not destabilize the functional structure of the enzyme. Notably, the number of hydrogen bonds between the ligand and the protein (Figure 5D) remained high and stable (approximately 3–8 hydrogen bonds throughout the simulation), indicating the formation of a robust polar interaction network. This is a key factor contributing to the stabilization of the complex and the enhancement of binding affinity. Due to its hydroxyl-rich structure, cistanoside D is capable of forming multiple simultaneous hydrogen bonds with residues in the active site of PARP1, thereby reinforcing long-term binding stability. These results suggest that cistanoside D exhibits strong and stable binding with PARP1, maintained by a combination of persistent hydrogen bonds and other non-covalent interactions. This mechanism indicates that this compound may act as a potential PARP1 inhibitor by occupying the NAD^+^ binding site, thereby interfering with the catalytic activity of the enzyme. Compared with the caspase-3 system, the interaction with PARP1 demonstrates significantly higher specificity and stability, highlighting the potential role of PARP1 as a primary molecular target of cistanoside D in its anticancer mechanism.

MM-PBSA analysis showed a clear preference of cistanoside D for PARP1 over caspase-3. The PARP1 complex exhibited a highly favorable binding free energy (ΔGtotal = −46.6 kcal/mol), whereas the caspase-3 complex showed negligible binding affinity (ΔGtotal = −0.19 kcal/mol) (Table 8). This difference was mainly driven by stronger van der Waals and electrostatic interactions in the PARP1 binding pocket. Consistently, MD simulations showed stable ligand retention, protein-ligand distance, and hydrogen-bond interactions for PARP1, while cistanoside D failed to maintain stable interactions with caspase-3. These findings support PARP1, rather than caspase-3, as the more plausible direct target of cistanoside D.

## 3. Discussion

Natural products continue to play an important role in anticancer drug discovery, serving both as direct therapeutic agents and as valuable sources of structurally diverse lead compounds [14,15]. In the present study, the cytotoxic potential of different fractions obtained from the bark of *Schima superba* was evaluated against several human cancer cell lines. Among the tested fractions, the ethyl acetate (EA) fraction exhibited the strongest cytotoxic activity against HepG2 and MCF-7 cells, while showing no detectable toxicity toward HEK293 cells at concentrations up to 300 μg/mL. These findings suggest that bioactive semi-polar constituents enriched in the EA fraction may contribute to the observed antiproliferative activity. Similar observations have been reported for numerous medicinal plants in which phenolic compounds, glycosides, and terpenoid derivatives concentrated in ethyl acetate fractions are associated with cytotoxic effects against cancer cells [16,17].

To investigate whether apoptosis contributes to the observed cytotoxicity, caspase-3 activation was examined in HepG2 cells. Caspase-3 is a key executioner protease responsible for the cleavage of multiple intracellular substrates during programmed cell death and is widely used as a biochemical marker of apoptosis [18]. Treatment with the EA fraction resulted in a concentration- and time-dependent increase in caspase-3 activity, with a more pronounced response observed after prolonged exposure (Figure 1). The gradual increase in enzyme activity suggests activation of apoptosis-associated signaling pathways following treatment with the extract. Although the present study did not evaluate additional apoptotic markers, the observed caspase-3 activation supports the possibility that apoptosis contributes, at least in part, to the cytotoxic effects of the EA fraction.

Previous phytochemical studies have demonstrated that *S. superba* contains a diverse array of secondary metabolites, including triterpenoid saponins, lignans, flavonoids, and phenolic glycosides [10,11,12,13,19]. Some of these constituents have been reported to exhibit cytotoxic, anti-inflammatory, or antimicrobial activities. In the present study, UPLC-QTOF-MS/MS analysis detected 18 major chromatographic peaks and enabled the tentative annotation of several compounds, including cistanoside D, periplocoside M, toosendanin, and yemuoside YM6. Compound annotation was based on accurate mass measurements, retention times, MS/MS fragmentation patterns, and comparison with available databases and published literature. Because authentic reference standards were not available, these assignments should be regarded as tentative rather than definitive identifications. The phytochemical composition observed in the present study differs in part from previous reports on *S. superba*, which may reflect variations in geographical origin, environmental conditions, harvesting season, plant chemotype, or extraction procedures. In particular, highly polar saponins previously reported as major constituents of *S. superba* may preferentially partition into aqueous or n-butanol fractions rather than the EA fraction.

The biological activity observed for the EA fraction is unlikely to be attributable to a single constituent. Instead, the cytotoxic effects may result from the combined contribution of multiple metabolites present within the extract. Phenylethanoid glycosides, terpenoid derivatives, and phenolic compounds identified in the fraction have previously been associated with modulation of cell survival pathways, oxidative stress responses, and apoptosis-related signaling. Therefore, synergistic or additive interactions among these constituents may contribute to the overall biological activity observed in the present study. To obtain preliminary insight into potential molecular mechanisms, selected compounds identified in the EA fraction were further evaluated using molecular docking against caspase-3 and PARP1 (Figure 2 and Figure 3). These proteins were selected because of their established roles in apoptosis and DNA damage response pathways [18,20,21,22]. Several compounds exhibited favorable predicted binding affinities toward both targets, with cistanoside D showing particularly strong predicted interactions. However, molecular docking is inherently a predictive computational approach and does not provide direct evidence of ligand binding or target modulation. Consequently, the docking results should be interpreted as hypothesis-generating observations that may assist in prioritizing compounds and targets for future experimental investigation.

To further assess the stability of the predicted interactions, molecular dynamics simulations were performed using cistanoside D as a representative compound. Interestingly, distinct interaction patterns were observed for the two protein targets. In the caspase-3 system, cistanoside D gradually dissociated from the predicted binding pocket during the simulation period, accompanied by increased ligand fluctuations and a reduction in intermolecular contacts. These findings suggest that stable direct binding between cistanoside D and caspase-3 is unlikely under the simulated conditions (Figure 4). This observation is consistent with the experimental finding that treatment with the EA fraction increased caspase-3 activity rather than suppressing it. Therefore, if cistanoside D contributes to apoptosis induction, its effects are more likely mediated through upstream cellular pathways that ultimately lead to caspase activation rather than through direct modulation of the catalytic site of caspase-3. In contrast, the cistanoside D-PARP1 complex remained relatively stable throughout the 200 ns simulation period (Figure 5). RMSD analysis, intermolecular contact profiles, hydrogen-bond occupancy, and MM/GBSA calculations collectively suggested persistent interactions between the ligand and the predicted binding region of PARP1 (Table 8). Because PARP1 is a key regulator of DNA repair and genomic stability, these observations raise the possibility that DNA damage response pathways may be involved in the biological effects of the EA fraction. Nevertheless, no direct biochemical or biophysical experiments were performed to confirm PARP1 inhibition or target engagement. Therefore, the computational findings should not be interpreted as evidence that cistanoside D acts as a PARP1 inhibitor, but rather as a rationale for future mechanistic studies.

The ADMET analysis provided additional information regarding the drug-likeness and pharmacokinetic characteristics of the selected compounds. Among the evaluated metabolites, periplocoside M displayed the most favorable overall profile, combining acceptable physicochemical properties with relatively favorable pharmacokinetic predictions. In contrast, cistanoside D and yemuoside YM6 exhibited multiple violations of Lipinski’s rule of five, suggesting potential limitations in oral bioavailability. Although toosendanin demonstrated favorable docking characteristics, its predicted toxicity profile was less favorable than those of several other compounds. These observations do not establish the therapeutic suitability of any individual constituent but may help prioritize compounds for future isolation, optimization, and biological evaluation.

Several limitations of this study should be acknowledged. First, compound annotation was based on UPLC-QTOF-MS/MS analysis and was not confirmed using authentic standards or NMR spectroscopy. Second, the biological assays were conducted using the EA fraction rather than purified compounds, preventing direct attribution of activity to individual metabolites. Third, the docking, molecular dynamics, and ADMET analyses represent computational predictions that require experimental validation. Despite these limitations, the combined phytochemical, biological, and computational findings provide a useful foundation for future bioassay-guided fractionation and mechanistic studies of *S. superba*.

## 4. Materials and Methods

### 4.1. Plant Material and Extraction

The stem bark of *Schima superba* was harvested in October 2024 from the mountainous area of Lao Bao District, Quang Tri Province, Vietnam. Botanical identification of the plant material was carried out by Dr. Tuan Anh Le (Vietnam Academy of Science and Technology, VAST). A voucher specimen has been deposited at the Laboratory of the Department of Pharmacognosy and Drug Control, Faculty of Pharmacy, School of Medicine and Pharmacy, University of Danang. The air-dried plant material was subjected to extraction using a combination of maceration and solvent–solvent partitioning techniques. Specifically, the dried stem bark was soaked in 96% ethanol at ambient temperature for seven days, and the process was repeated three times to ensure exhaustive extraction. After each extraction cycle, the solution was filtered, and all filtrates were combined. The pooled extract was then concentrated under reduced pressure using a rotary evaporator to yield the crude ethanolic extract. Subsequently, the crude extract was reconstituted in a hydroethanolic solution and fractionated sequentially with solvents of increasing polarity, including n-hexane, dichloromethane, and ethyl acetate, leaving a final aqueous fraction. Each partitioning step was performed in triplicate. The respective organic fractions were collected separately and concentrated under reduced pressure to obtain the n-hexane, dichloromethane, ethyl acetate, and aqueous residue fractions.

### 4.2. Chemicals and Equipment

Cell culture experiments were performed using Dulbecco’s Modified Eagle Medium (DMEM) or Minimum Essential Medium with Eagle’s salts (MEM), both supplemented with L-glutamine, sodium pyruvate, sodium bicarbonate (NaHCO3), penicillin/streptomycin, and 10% fetal bovine serum (FBS). Trypsin–EDTA solution (0.05%) was used for routine cell detachment and subculturing. The study utilized standard laboratory equipment, including an inverted microscope (Axiovert 40 CFL, Carl Zeiss, Gottingen, Germany), a phase-contrast inverted microscope (EUROMEX OX.2053-PLPH, Duiven, The Netherlands), a hemocytometer (Fisher Scientific, Waltham, MA, USA) for cell counting, and a microplate spectrophotometer (BioTek, Winooski, VT, USA) for absorbance measurements. Cell cultures were maintained in a humidified CO_2_ incubator, while samples and reagents were stored using a −80 °C deep freezer and liquid nitrogen system. Additional equipment included an analytical balance, a pH meter, and other conventional laboratory instruments. Analytical-grade reagents were used throughout the study, including dimethyl sulfoxide (DMSO), trichloroacetic acid (TCA), Tris base, phosphate-buffered saline (PBS), ellipticine (used as a positive control), sulforhodamine B (SRB), and acetic acid.

### 4.3. UPLC-QTOF-MS/MS Analysis

Chromatographic separation was achieved via an Acquity UPLC I-class Plus/Xevo G2-XS Q-ToF system (Waters Corp.; Milford, MA, USA). Chromatographic separation was conducted at 40 °C with a Waters HSS T3 column (C18, 2.1 100 mm, 1.8 μm), the injection volume was 5 µL, and the flow rate was 0.4 mL/min. The mobile phases consisted of water containing 0.1% formic acid (A) and acetonitrile containing 0.1% formic acid (B) (Merck, Darmstadt, Germany). The gradient program was as follows: 5% B (0–2 min), 5–40% B (2–10 min), 40–95% B (10–20 min), 95% B (20–25 min), 95–10% B (25–26 min), and 10% B (26–30 min) [23]. Mass spectrometric analysis was performed using electrospray ionization in positive mode (ESI+). The instrument was operated in the mass range of *m*/*z* 100–1200 Da. The capillary voltage was set to 3.0 kV, the cone voltage to 40 V, the source temperature to 125 °C, and the desolvation temperature to 350 °C. Cone gas and desolvation gas flows were maintained at 50 L/h and 650 L/h, respectively. MS/MS fragmentation was conducted with a collision energy range of 10–40 eV [24,25]. Data acquisition and processing were performed using MassLynx 4.1 software (Waters Corp.; Milford, MA, USA), while compound identification was carried out with UNIFI NPAS software in combination with multiple spectral libraries, including the University of Ottawa Natural Product Library, the University of Mississippi Natural Product Library, and the Green Tea Library. Identification was further supported by cross-referencing with online chemical databases such as ChemSpider (www.chemspider.com), PubMed (http://www.ncbi.nlm.nih.gov/pubmed (accessed on 20 April, 2026)), and MassBank (http://www.massbank.jp (accessed on 20 April, 2026)). Stringent criteria (mass error ≤ ±5 ppm, response intensity ≥ 5000 counts, RMR ≤ 20 ppm, and RMS ≤ 15 ppm) were applied to ensure reliable identification. In addition, [M + H]^+^ ion fragments, retention time, mass spectral databases, and relevant literature were used to confirm molecular formulas and structural assignments.

### 4.4. Cell Lines

The human cancer cell lines used in this study included HepG2 (hepatocellular carcinoma), MCF-7 (breast adenocarcinoma), and A549 (lung carcinoma). These cell lines were originally obtained from the American Type Culture Collection (ATCC, Manassas, VA, USA). The cells were kindly provided by Prof. J. M. Pezzuto (Long Island University, USA) and Prof. Jeanette Maier (University of Milan, Italy).

### 4.5. In Vitro Cytotoxicity Assay

The in vitro cytotoxic potential was evaluated using the Sulforhodamine B (SRB) assay, a standard protocol validated by the National Cancer Institute (NCI) for screening agents that inhibit cell growth or induce cell death [26]. This assay quantifies total cellular protein content based on optical density (OD) measurements after staining with SRB. The measured OD value is directly proportional to the amount of SRB bound to cellular proteins, reflecting the cell population density. Test samples were dissolved in 100% DMSO to prepare stock solutions of 20 mM (for pure compounds) or 20 mg/mL (for extracts). These stocks were further diluted in a culture medium (without FBS) across four serial concentrations. Targeted cells were harvested using trypsinization and adjusted to the required density using a hemocytometer. Subsequently, 190 µL of cell suspension (supplemented with 5% FBS) was seeded into 96-well plates and incubated in a 5% CO_2_ incubator for 18–20 h to ensure stable attachment. Following the stabilization period, 10 µL of the diluted test samples were added to the designated wells. Control wells (Day 0) contained 190 µL of cell suspension and 10 µL of 1% DMSO; these were fixed with 20% trichloroacetic acid (TCA) after 1 h of treatment. The remaining plates were incubated for 48 h before being fixed with cold 20% (*v*/*v*) TCA for 1 h. After removing the TCA and washing with dH_2_O, the plates were air-dried at room temperature. The cells were then stained with 0.4% (*w*/*v*) SRB in 1% acetic acid for 30 min at 37 °C. Residual dye was removed by washing three times with 1% acetic acid. The bound SRB was solubilized in 200 µL of 10 mM unbuffered Tris base. After gentle shaking for 10 min, the OD values were recorded at 540 nm using an ELISA Plate Reader (BioTek, Winooski, VT, USA). The percentage of growth inhibition was calculated using the following formula:

$$\%Inhibition=100-(\frac{ODt-ODo}{ODdmso-ODo})\;\times \;100$$

where ODt, ODc, and ODo represent the optical density of the test sample, control, and Day 0, respectively.

All assays were performed in triplicate to ensure reproducibility. Ellipticine at concentrations of 10, 2, 0.4, and 0.08 µL served as the positive control, while 1% DMSO (final concentration in wells at 0.05%) was used as the negative control. Half-maximal inhibitory concentration, IC_50_, values were determined using TableCurve 2D v4 software.

### 4.6. Caspase-3 Activity Assay

Caspase-3 activation was determined using a fluorometric assay based on cleavage of the Ac-DEVD-AFC substrate. HEPG2 cells (1 × 10^6^ cells/well) were treated with EA (5–100 µg/mL) for 12, 24, and 48 h. Cells were harvested, washed with PBS, and lysed. Reaction buffer and fluorogenic substrate were added, and samples were incubated at 37 °C for 1 h. Fluorescence was measured at 370 nm (excitation) and 505 nm (emission) [27]. Caspase-3 activity was expressed as the fold increase relative to untreated control cells. Data were analyzed using GraphPad Prism 10.

### 4.7. Molecular Simulation Methods

Docking and MD simulations were performed following our previously established protocol [28,29]. Briefly, PARP1 (PDB ID: 7KK4) and caspase-3 (PDB ID: 2XYP) structures were obtained from the Protein Data Bank. The grid box parameters were set at x = −9.44, y = 6.11, and z = 27.54, and dimensions of 20 × 30 × 20 Å and at x = 35.89, y = 36.00, and z = 32.28, with dimensions of 30 × 15 × 15 Å for PARP1 and Caspase-3, respectively. Docking was conducted using AutoDock Vina 1.2.3 with predefined grid boxes for each target, and binding poses were visualized in Discovery Studio Visualizer 2020 (see Supporting Information file). Selected complexes were then subjected to 200 ns MD simulations using GROMACS 2020.4. Systems were prepared through CHARMM-GUI with the CHARMM36 force field, solvated using the TIP3P water model, neutralized with KCl ions, and simulated at 300 K and 1 atm. Trajectories and energies were recorded every 10 ps, and binding free energies were estimated using the MM/GBSA approach with gmx_MM.PBSA.

### 4.8. ADMET Properties and Physicochemical Parameters

In the present study, Lipinski’s Rule of Five was applied to assess the drug-likeness of the identified phytoconstituents [30]. A combination model was used to predict the pharmacological potential of the compounds. The parameters were retrieved by ADMET properties. In this study, the pkCSM web-based platform (https://biosig.lab.uq.edu.au/pkcsm/prediction (accessed on 20 April 2026)) was utilized to predict pharmacokinetic behaviour and toxicity endpoints. The predictions were generated using the simplified molecular-input line-entry system (SMILES) representations of the compounds as input data [30].

### 4.9. Statistical Analysis

Analysis of variance (ANOVA) followed by Tukey’s test was performed on pre-validated data. Additionally, statistical analysis was conducted using Prism 10 (GraphPad Software, San Diego, CA, USA) with a *t*-test for comparisons between two groups or ANOVA followed by Bonferroni post hoc analysis for multiple group comparisons and correlation analysis. Data were expressed as mean ± standard deviation (SD).

## 5. Conclusions

In conclusion, the present study demonstrates that the ethyl acetate fraction derived from Vietnamese *Schima superba* bark exhibits potent and selective cytotoxic effects against HepG2 and MCF-7 cancer cells, while showing negligible toxicity toward normal HEK293 cells. The observed time- and dose-dependent activation of caspase-3 indicates that apoptosis is the primary mechanism underlying its anticancer activity. Phytochemical profiling using UPLC-QTOF-MS/MS identified 18 major constituents, predominantly phenylethanoid glycosides and triterpenoids. Importantly, the integration of in silico approaches targeting two key proteins involved in apoptosis pathways, namely caspase-3 and PARP1, provided critical mechanistic insights into the molecular targets of these compounds. Among them, cistanoside D was selected as a promising candidate not only due to its high binding affinity, ranking among the top-performing compounds, but also because it exhibited an initial dual-binding tendency toward both targets at the docking stage. Further analyses clarified its mechanistic selectivity, cistanoside D was identified as a stable and energetically favorable ligand for PARP1 based on 200 ns molecular dynamics simulations and MM-PBSA analysis. The strong binding affinity, together with a persistent hydrogen-bonding network, suggests that cistanoside D may interact with PARP1. In contrast, the lack of stable interaction with caspase-3 supports the hypothesis that the observed caspase activation in vitro is a downstream consequence of impaired DNA repair rather than direct enzymatic targeting. Collectively, these findings highlight *Schima superba* as a promising source of natural pro-apoptotic agents and identify PARP1 as a potential molecular target of cistanoside D. This study provides a solid scientific foundation for further pharmacological validation, structural optimization, and in vivo investigations, ultimately contributing to the development of novel anticancer therapies targeting DNA repair pathways.

## Figures and Tables

**Table 1:** Cytotoxic activity of fractions from the bark of *S. superba*.

Fractions	Cells, IC_50_ (μg/mL) ^a^			
	A549	HepG2	MCF-7	HEK293
MeOH	75.36 ± 1.89	36.86 ± 1.82	48.05 ± 2.09	>300
n-Hexane	>100	67.87 ± 3.15	76.37 ± 3.04	>300
Dimethylene chloride	70.10 ± 2.71	57.82 ± 2.12	65.44 ± 2.56	>300
Ethyl acetate	45.30 ± 2.31	27.98 ± 1.63	29.46 ± 0.87	>300
Ellipticine ^b^	0.40 ± 0.03	0.32 ± 0.03	0.35 ± 0.03	-

^a^ IC_50_ values were calculated from three independent experiments and are expressed in µg/mL. ^b^ Ellipticine was used as a positive control, and its IC_50_ values are expressed in µM. Data are presented as mean ± SD (*n* = 3) within acceptable experimental variation. (-): no test.

**Table 2:** Phytochemical constituents of EA fraction.

No	Compound Names	Formula	Classification
1	1,7-Diphenyl-5-hydroxy-4,6-heptadien-3-one	C_19_H_18_O_2_	Polyphenol
2	19-Acetoxy-9(11),15-pimaradiene	C_22_H_34_O_2_	Diterpenoid
3	2,4,7-Trimethoxy phenanthrene	C_17_H_16_O_3_	Phenolic
4	2,6-Dimethylaniline	C_8_H_11_N	Others
5	2-Carboxymethyl-3-prenyl-2,3-epoxy-1,4-naphthpeuinone	C_17_H_16_O_4_	Anthranoid
6	7-O-(3,3-Dimethylallyl)-scopoletin	C_15_H_16_O_4_	Coumarin
7	Cistanoside D	C_31_H_40_O_15_	Phenolic
8	Eleutherazine B	C_22_H_36_N_4_O_8_	Others
9	Flavokawain B	C_17_H_16_O_4_	Phenolic
10	Glucosinalbin	C_14_H_19_NO_10_S_2_	Others
11	Hispidin	C_13_H_10_O_5_	Phenolic
12	Nuezhenidic acid	C_17_H_24_O_14_	Others
13	Periplocoside C	C_49_H_76_O_16_	Steroid
14	Periplocoside M	C_34_H_52_O_9_	Steroid
15	Phytolaccagenin	C_31_H_48_O_7_	Triterpenoid
16	Toosendanin_1	C_30_H_38_O_11_	Triterpenoid
17	Yadanzioside A	C_32_H_44_O_16_	Terpenoid
18	Yemuoside YM6	C_31_H_42_O_16_	Triterpenoid

**Table 3:** Interactions between compounds and caspase 3.

Compound	Best Binding Energy (kcal/mol)	H-Bond Interactions	Hydrophobic Interactions	Other Interactions
1,7-Diphenyl-5-hydroxy-4,6-heptadien-3-one	−4.867		HIS121, CYS163	MET61
19-Acetoxy-9(11),15-pimaradiene	−5.861	TYR204, ARG207	HIS121, CYS163	
2,4,7-Trimethoxy phenanthrene	−5.626	CYS163	MET61, HIS121, PHE128, CYS163	CYS163
2,6-Dimethylaniline	−3.944	HIS121	HIS121, CYS163, TYR204	ARG207
2-Carboxymethyl-3-prenyl-2,3-epoxy-1,4-naphthoquinone	−6.205	GLY122, CYS163, GLY165	HIS121, LEU168, TYR204, PHE256	MET61
7-O-(3,3-Dimethylallyl)-scopoletin	−6.075	ARG64, GLN161, ARG207	HIS121, CYS163, TYR204, TRP206, ARG207	ARG207
Cistanoside D	−6.688	THR62, GLY122, GLU123, THR166, TYR204	MET61, HIS121, CYS163, LEU168	
Eleutherazine B	−6.504	MET61, THR62, GLU123, GLY165	ARG64, HIS121, TYR204	
Flavokawain B	−5.899	ARG207	MET61, HIS121, PHE128, CYS163, ARG207	ARG207
Glucosinalbin	−6.539	HIS121, CYS163, GLY165, SER205, ARG207	HIS121, TYR204	CYS163
Hispidin	−5.911	TYR204, ARG207	HIS121, CYS163, TYR204	ARG207
Nuezhenidic acid	−5.683	THR62, GLY122, GLU123, ARG207		
Periplocoside C	−5.515	HIS121, GLU123, CYS163, THR166	HIS121, CYS163, TYR204	
Periplocoside M	−6.749	THR62	HIS121, PHE128, CYS163, LEU168, PHE256	
Phytolaccagenin	−6.019	CYS163, TYR204, SER205	CYS163, TYR204, PHE256	
Toosendanin	−6.749	GLY60, MET61, THR62, HIS121, CYS163, ARG207	MET61, HIS121	
Yadanzioside A	−5.58	THR166	HIS121, PHE128, LEU168, TYR204, PHE256	
Yemuoside YM6	−6.695	ARG64, HIS121, CYS163, THR166, SER205, ARG207	PHE256	
Ellipticine ^a^	−6.084	GLY60	MET61, PHE128	MET61, CYS163

^a^ Positive control.

**Table 4:** Interactions between compounds and PARP1.

Compound	Best Binding Energy (kcal/mol)	H-Bond Interactions	Hydrophobic Interactions	Other Interactions
1,7-Diphenyl-5-hydroxy-4,6-heptadien-3-one	−7.35	ASN767, ASN868	HIS862, TYR907	
19-Acetoxy-9(11),15-pimaradiene	−9.399	ARG878	HIS862, TYR889, TYR896, TYR907	
2,4,7-Trimethoxy phenanthrene	−8.163	ASN767, ASN868	HIS862, TYR896, TYR907	
2,6-Dimethylaniline	−5.592		HIS862, ALA898, TYR907	
2-Carboxymethyl-3-prenyl-2,3-epoxy-1,4-naphthoquinone	−8.985	ASN767, SER864, ARG865, ASN868	HIS862, TYR907	ASP766
7-O-(3,3-Dimethylallyl)-scopoletin	−8.107	HIS862, GLY863, SER904	HIS862, TYR889, TYR896, ALA898, TYR907	
Cistanoside D	−10.954	TYR710, GLY863, SER864, ASN868, ARG878, ALA880, PRO881, GLY894, LYS903	LEU769, HIS862, ARG878, PRO881, TYR907, HIS909	ASP770, ARG878
Eleutherazine B	−9.195	HIS862, ASN868, TYR889, ILE895, TYR896, TYR907	TYR889, TYR896, TYR907	
Flavokawain B	−7.941	TYR689, ASN767, HIS862, GLY863, HIS909	ARG865, ALA898, TYR907, HIS909	
Glucosinalbin	−9.126	ASP766, ASN767, ASP770, SER864, ARG865, ASN868, ARG878, HIS909	HIS862, TYR907	HIS862
Hispidin	−8.838	SER864, ASN868, PHE897	HIS862, TYR907	
Nuezhenidic acid	−8.576	ASP766, HIS862, GLY863, SER904, TYR907, GLU988	TYR907	
Periplocoside C	−10.351	TYR710, ASP766, ASN906	HIS826, HIS862, ALA880, TYR889, TYR896, TYR907, LEU985	
Periplocoside M	−11.287	LYS903	LEU769, HIS826, MET890, TYR896, TYR907, LEU984	
Phytolaccagenin	−9.636	ASP766, ASN868, TYR907, GLU988	TYR907	
Toosendanin	−9.164	HIS862, SER864, TYR896	HIS862, TYR896	ASP770, ARG878
Yadanzioside A	−9.198	ASP770, HIS862, ARG878, MET890, TYR907	HIS862, TYR896, TYR907	MET890
Yemuoside YM6	−10.489	GLU763, ASP766, ASN767, ASP770, HIS862, TYR907	HIS862, MET890, TYR896, TYR907	
Ellipticine ^a^	−9.98	GLY863	HIS862, TYR896, ALA898, TYR907	

^a^ Positive control.

**Table 5:** Physicochemical and ADME properties of selected compounds.

Compound	MW (g/mol)	Log P	nHBD	nHBA	TPSA	MR	Lipinski Violation	Log S	nRotB
Cistanoside D	652.64	0.17	7	15	223.29	157.36	3	−3.32	13
Periplocoside M	604.77	3.40	3	9	123.91	160.55	1	−5.63	6
Toosendanin	574.62	1.36	3	11	165.26	138.59	2	−3.59	5
Yemuoside YM6	670.66	−1.11	10	16	257.68	157.36	3	−2.31	11
Ellipticine ^a^	246.31	3.92	1	1	28.68	81.04	0	−5.05	0

Molecular weight (MW), partition coefficient (Log P), hydrogen bond donors/acceptors (nHBD/nHBA), total polar surface area (TPSA), molar refractivity (MR), solubility (Log S), and rotatable bonds (nRotB). ^a^ Positive control.

**Table 6:** ADME predictions of compounds.

				Inhibitor Interaction					
Compound	Log K_p_ (cm/s)	GI Abs	BBB per	P-gp Substrate	CYP1A2 Inhibitor	CYP2C19 Inhibitor	CYP2C9 Inhibitor	CYP2D6 Inhibitor	CYP3A4 Inhibitor
Cistanoside D	−10.17	Low	No	Yes	No	No	No	No	No
Periplocoside M	−7.25	High	No	Yes	No	No	No	No	No
Toosendanin	−9.32	Low	No	Yes	No	No	No	Yes	No
Yemuoside YM6	−11.69	Low	No	Yes	No	No	No	No	No
Ellipticine ^a^	−4.39	High	Yes	Yes	Yes	Yes	No	Yes	Yes

Log Kp: log of skin permeability; GI Abs: Gastro-intestinal absorption; BBB Per: Blood–brain barrier permeability; P-gp: P-glycoprotein; CYP: cytochrome-P. ^a^ Positive control.

**Table 7:** Toxicity of compounds.

Compound	LD_50_ (mg/kg)	Toxicity Class
Cistanoside D	5000	Class V: may be harmful if swallowed
Periplocoside M	500	Class IV: harmful if swallowed
Toosendanin	244	Class III: toxic if swallowed
Yemuoside YM6	823	Class IV: harmful if swallowed
Ellipticine ^a^	940	Class IV: harmful if swallowed

^a^ Positive control.

**Table 8:** Summary of the interaction energies and the binding free energy for two complexes (kcal/mol).

Energies	Cistanoside D-Caspase-3	Cistanoside D-PARP1
Δ$G_{sol}$	5.71	68.55
Δ$G_{gas}$	−5.9	−115.15
Δ$G_{total}$	−0.19	−46.6

## Data Availability

The original contributions presented in this study are included in this article. Further inquiries can be directed to the corresponding author.

## Supplementary Materials

The following supporting information can be downloaded at: https://www.mdpi.com/article/10.3390/molecules31142550/s1.

## Abbreviations

The following abbreviations are used in this manuscript: EA, ethyl acetate; DMSO, dimethyl sulfoxide; PARP1, Poly (ADP-ribose) polymerase; IC_50_, 50% inhibitory concentration; UPLC-QTOF-MS/MS, ultra-performance liquid chromatography quadrupole time of flight mass spectrometry; MD, molecular dynamic; SRB, Sulforhodamine B; OD, optical density; MM-PBSA, Molecular Mechanics-Poisson Boltzman Surface Area; HepG2, hepatocellular carcinoma; MCF-7, breast adenocarcinoma; A549, lung carcinoma; HEK; ATCC, American Type Culture Collection; DMEM, Dulbecco’s Modified Eagle Medium; MEM, Minimum Essential Medium; RMSD, Root Mean Square Deviation; RMSF, Root Mean Square Fluctuation.
