# Supplementary material for: Phytochemical Characterization and Cytotoxic Potential of the Ethyl Acetate Fraction of Schima superba Bark: An In Vitro and In Silico Investigation
Source: Molecules. 2026 Jul 22;31(14):2550. doi: 10.3390/molecules31142550 (PMC13415642; doi:10.3390/molecules31142550)
Supplement: Supplementary file 1 [file molecules-31-02550-s001.zip › molecules-4345907-supplementary.pdf]

Supporting Information to:

Phytochemical Characterization and Cytotoxic Potential of the Ethyl Acetate Fraction of *Schima superba* Bark: An *In Vitro* and *In Silico* Investigation

Hieu Phu Chi Truong [1], Hong Khuyen Thi Pham [1], Thuy Mi Pham Lam [2], Tuan Anh Le [3], Van Ngo Thai Bich [4], Phu Tran Vinh Pham [5], Tan Khanh Nguyen [6], Kim Lien Thi Giang [5] and Manh Hung Tran [1,*]

[1] Faculty of Pharmacy, School of Medicine and Pharmacy, The University of Danang, Danang City 550000, Vietnam; tpchieu@smp.udn.vn (H.P.C.T.); phamthihongkhuyendtht123@gmail.com (H.K.T.P.)
[2] Faculty of Physics and Chemistry, University of Education, The University of Danang, Danang City 550000, Vietnam; 3144122013@ued.udn.vn
[3] Mientrung Institute for Scientific Research, Vietnam National Museum of Nature, Vietnam Academy of Science and Technology (VAST),
Hue City 530000, Vietnam; ltanh@misr.vast.vn
[4] Faculty of Chemical Engineering, University of Science and Technology, The University of Danang, Danang City 550000, Vietnam; ntbvan@dut.udn.vn
[5] Biomedical Science Department, VN-UK Institute for Research and Executive Education, The University of Danang, Danang City 550000, Vietnam; phu.pham@vnuk.udn.vn (P.T.V.P.); lien.giang@vnuk.edu.vn (K.L.T.G.)
[6] Institute for Biocomputation and Physics of Complex Systems, University of Zaragoza,
50009 Zaragoza 50018, Spain; khanhnt@donga.edu.vn
* Correspondence: tmhung@smp.udn.vn

Docking simulation information

Molecular docking done by UCSF Chimera 1.19, AutoDock Vina 1.2.7, Windows 11, 13th Gen Intel(R) Core(TM) i7-13620H (2.40 GHz)

- Compound characteristic

No.	Compound	PubChem CID	SMILES
1	1,7-Diphenyl-5-hydroxy-4,6-heptadien-3-one	45483921	C1=CC=C(C=C1)CCC(=O)/C=C(\C=C\C2=CC=CC=C2)/O
2	19-Acetoxy-9(11),15-pimaradiene		C[C@@]1(COC(C)=O)CCC[C@]2(C)C3=CC[C@](C=C)(C)C[C@]3([H])CCC12
3	2,4,7-Trimethoxy phenanthrene	15693458	COC1=CC2=C(C=C1)C3=C(C=C(C=C3C=C2)OC)OC
4	2,6-Dimethylaniline	6896	CC1=C(C(=CC=C1)C)N
5	2-Carboxymethyl-3-prenyl-2,3-epoxy-1,4-naphthoquinone		O=C(C1=C2C=CC=C1)C3(C/C=C(C)\C)C(O3)(CC(O)=O)C2=O

6	7-O-(3,3-Dimethylallyl)-scopoletin	1764914	CC(=CCOC1=C(C=C2C=CC(=O)OC2=C1)OC)C
7	Cistanoside D	5315930	C[C@H]1[C@@H]([C@H]([C@H]([C@@H](O1)O[C@@H]2[C@H]([C@@H](O[C@@H]([C@H]2OC(=O)/C=C/C3=CC(=C(C=C3)O)OC)CO)OCCC4=CC(=C(C=C4)O)OC)O)O)O)O
8	Eleutherazine B	20839739	C/C(=C\C(=O)NCCCC1NC(=O)C(NC1=O)CCCNC(=O)/C=C(/CCO)\C)/CCO
9	Flavokawain B	5356121	COC1=CC(=C(C(=C1)OC)C(=O)/C=C/C2=CC=CC=C2)O
10	Glucosinalbin	9601115	C1=CC(=CC=C1C/C(=N\OS(=O)(=O)O)/S[C@H]2[C@@H]([C@H]([C@@H]([C@H](O2)CO)O)O)O)O
11	Hispidin	54685921	C1=CC(=C(C=C1/C=C/C2=CC(=CC(=O)O2)O)O)O
12	Nuezhenidic acid	133561674	COC(=O)C1=COC(C(C1CC(=O)O)(CC(=O)O)O)OC2C(C(C(C(O2)CO)O)O)O
13	Periplocoside C	163943	CC1C=C(C(=O)C(O1)OC2CCC3(C4CCC5(C(C4CC=C3C2)CCC5(C(C)OC6CC7C(C(O6)C)OOC8(CC(C(C(O8)C)OC9CC(C(C(O9)C)O)OC)OC)CO7)O)C)C)OC
14	Periplocoside M	131845091	C[C@@H]1C=C(C(=O)[C@@H](O1)O[C@H]2CC[C@@]3([C@H]4CC[C@]5([C@H]([C@@H]4CC=C3C2)CC[C@@]5([C@H](C)O[C@H]6C[C@H]([C@@H]([C@H](O6)C)O)O)O)C)C)OC
15	Phytolaccagenin	21594228	C[C@@]1(CC[C@@]2(CC[C@@]3(C(=CC[C@H]4[C@]3(CC[C@@H]5[C@@]4(C[C@@H]([C@@H]([C@@]5(C)CO)O)O)C)C)[C@@H]2C1)C)C(=O)O)C(=O)OC
16	Toosendanin	9851101	CC(=O)O[C@@H]1C[C@@H]([C@@]23CO[C@H]([C@@]1([C@@H]2C[C@H]([C@@]4([C@@H]3C(=O)[C@@H]([C@@]5([C@]46[C@H](O6)C[C@H]5C7=COC=C7)C)OC(=O)C)C)O)C)O)O
17	Yadanzioside A	72956	C[C@H]1[C@@H]2C[C@@H]3[C@@]45CO[C@]([C@@H]4[C@H](C(=O)O3)OC(=O)CC(C)C)([C@H]([C@@H]([C@@H]5[C@]2(C=C(C1=O)O[C@H]6[C@@H]([C@H]([C@@H]([C@H](O6)CO)O)O)O)C)O)O)C(=O)OC
18	Yemuoside YM6	15613263	COC1=C(C=C2C(C(C(CC2=C1)(COC3C(C(C(C(O3)COC4C(C(CO4)(CO)O)O)O)O)O)CO)C5=CC(=C(C=C5)O)OC)O
REF	Ellipticine	3213	CC1=C2C=CN=CC2=C(C3=C1NC4=CC=CC=C43)C

- Poly(ADP-ribose) polymerase 1

PDB ID: 7KK4

Co crystal structure: Olaparib

Discovery Studio Visualizer 2025 was used to determine the binding site of the protein, which consists of some residues of chain A of the protein:

710,766,769,861,862,863,878,879,880,881,889,894,895,896,897,898,903,904,907,988

Based on these residues, the center of grid box was provided by UCSF Chimera. The size of grid box was set to cover the binding site of the protein.

Grid box	x	y	z
Center	-9.44	6.11	27.54
Size	20	30	20

A

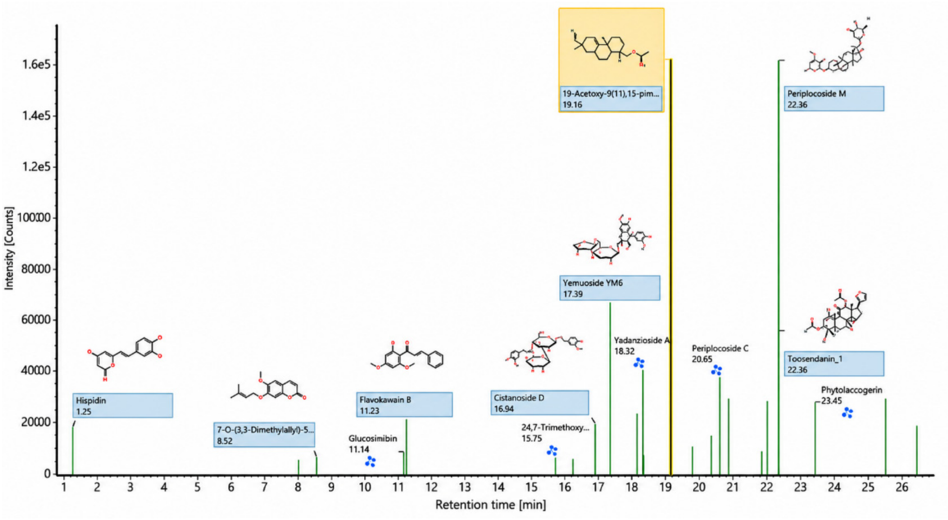

B

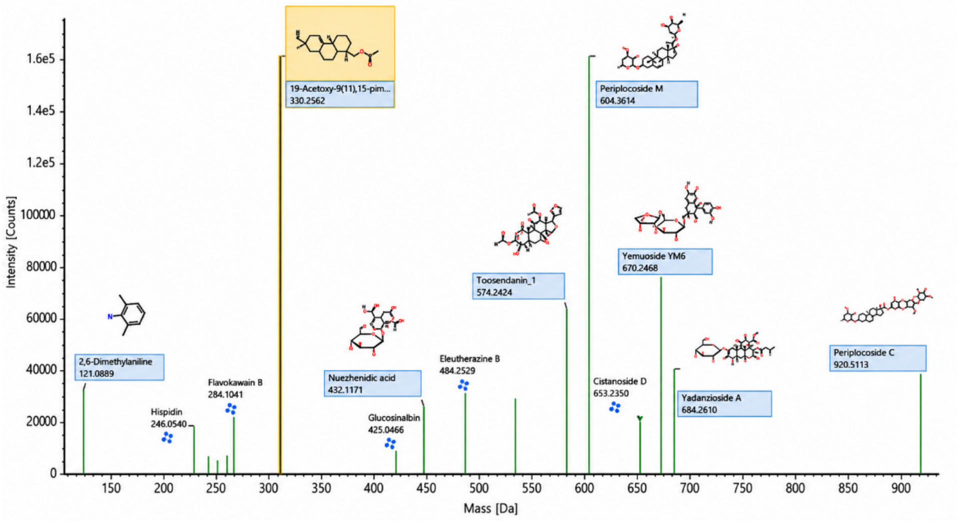

Figure S1. UPLC–QTOF–MS/MS phytochemical profiling of the ethyl acetate (EA) fraction from *Schima superba*. (**A**) Representative chromatogram showcasing the retention times of identified secondary metabolites; (**B**) Mass spectrum (MS) illustrating the distribution of detected compounds based on their mass-to-charge (*m*/*z*) ratios. Major identified peaks including cistanoside D, yemuoside YM6, and periplocoside M are annotated.
