# Supplementary material for: Phytochemical Characterization and Cytotoxic Potential of the Ethyl Acetate Fraction of Schima superba Bark: An In Vitro and In Silico Investigation
Source: Molecules. 2026 Jul 22;31(14):2550. doi: 10.3390/molecules31142550 (PMC13415642; doi:10.3390/molecules31142550)
Supplement: Supplementary file 1 [file molecules-31-02550-s001.zip › Supporting information-LCMS data.pdf]

Created by: Administrator, UNIFI

Created on: May 08, 2026

Created time: 14:44:42 SE Asia Standard Time

Item name: La_VT_08052026

Analysis Information

Item name:	La_VT_08052026	Analysis Method Item name:	MS_ESI+_12112025
Version:	2	Analysis Method Version:	1
Modified date:	May 08, 2026 14:19:48 SE Asia Standard Time	Sample Set Created date:	
Modified by:	Administrator, UNIFI	Sample Set Instrument system name:	
Folder:	Company/sponge		

Analysis injection list

.	Item name	Item description	Sample type	Replicate number	Sample position	Injection volume (μL)	Acquisition status	Limit status
1	blank_EtOH_10042026		Reference	1	1:25	5.00	Complete	No Checks Performed
2	La_VT_Ea_10042026		Unknown	1	1:29	5.00	Complete	No Checks Performed

Item name: La_VT_08052026

Item name: La_VT_Ea_10042026

.	Retention time (min)	Area	% Area (%)	Height	Width (s)	Integration type	Peak width parameter (s)	Threshold parameter
1	25.48	1263512	21.04	48730	32.72	VV	80.64	1491.60
2	26.46	1305146	21.60	82149	75.85	VB	80.37	1469.98
3	1.24	785291	90.49	174200	12.61	VV	8.06	32.93
4	8.51	266098	100.00	60951	23.10	BB	8.43	33.42
5	8.00	234890	100.00	31669	26.90	BB	8.43	33.27
6	15.76	204123	8.01	14802	26.80	VV	22.25	335.10
7	18.32	299983	95.04	30489	25.84	BV	10.39	128.36
8	11.23	796404	16.10	64169	27.96	BV	17.61	258.56
9	16.25	227059	4.60	23635	23.10	VV	17.61	261.91
10	19.80	542041	6.44	65111	27.33	VV	11.70	79.10
11	20.38	614793	7.30	98281	22.66	VV	11.70	79.64
12	19.16	6662248	79.09	1038442	27.43	VV	11.71	82.60
13	21.84	315689	99.84	56678	20.55	BV	11.71	82.89
14	11.13	315675	34.32	67513	28.49	BV	8.18	17.66
15	18.15	714519	59.24	119670	15.78	BV	11.23	133.70
16	20.88	942929	13.87	160225	16.93	VV	9.19	57.94
17	23.44	661975	26.54	62182	31.56	VV	16.71	244.84
18	22.06	724047	17.94	63834	17.37	VV	15.91	252.06
19	22.36	2556499	63.35	244824	29.02	VV	15.91	241.18
20	22.35	5448791	29.37	645149	29.45	VV	11.75	62.69
21	16.93	567746	28.54	69638	21.08	VV	13.99	174.04
22	17.40	1974370	43.35	226541	22.13	VV	15.42	199.17
23	18.31	1187773	44.09	178697	17.37	VV	11.68	78.01
24	20.67	1047320	13.62	55669	25.22	VV	24.55	201.08

Item name: La_VT_Ea_10042026, Sample position: 1:29, Replicate number: 1

.	Component name	Formula	Identification status	Neutral mass (Da)
1	1,7-Diphenyl-5-hydroxy-4,6-heptadien-3-one	C19H18O2	Identified	278.13068
2	19-Acetoxy-9(11),15-pimaradiene	C22H34O2	Identified	330.25588
3	19-Acetoxy-9(11),15-pimaradiene	C22H34O2	Identified	330.25588
4	19-Acetoxy-9(11),15-pimaradiene	C22H34O2	Identified	330.25588
5	19-Acetoxy-9(11),15-pimaradiene	C22H34O2	Identified	330.25588
6	2,4,7-Trimethoxy phenanthrene	C17H16O3	Identified	268.10994
7	2,6-Dimethylaniline	C8H11N	Identified	121.08915
8	2,6-Dimethylaniline	C8H11N	Identified	121.08915
9	2-Carboxymethyl-3-prenyl-2,3-epoxy-1,4-naphthpeuinone	C17H16O4	Identified	284.10486
10	7-O-(3,3-Dimethylallyl)-scopoletin	C15H16O4	Identified	260.10486
11	7-O-(3,3-Dimethylallyl)-scopoletin	C15H16O4	Identified	260.10486
12	Cistanoside D	C31H40O15	Identified	652.23672
13	Eleutherazine B	C22H36N4O8	Identified	484.25331
14	Flavokawain B	C17H16O4	Identified	284.10486
15	Glucosinalbin	C14H19NO10S2	Identified	425.04504

.	Component name	Formula	Identification status	Neutral mass (Da)
16	Hispidin	C13H10O5	Identified	246.05282
17	Nuezhenidic acid	C17H24O14	Identified	452.11661
18	Periplocoside C	C49H76O16	Identified	920.51334
19	Periplocoside M	C34H52O9	Identified	604.36113
20	Phytolaccagenin	C31H48O7	Identified	532.34000
21	Toosendanin_1	C30H38O11	Identified	574.24141
22	Toosendanin_1	C30H38O11	Identified	574.24141
23	Yadanzioside A	C32H44O16	Identified	684.26294
24	Yemuoside YM6	C31H42O16	Identified	670.24729

.	Component name	Formula	Observed neutral mass (Da)	Observed m/z	Mass error (mDa)
1	1,7-Diphenyl-5-hydroxy-4,6-heptadien-3-one	C19H18O2	278.1315	279.1388	0.8
2	19-Acetoxy-9(11),15-pimaradiene	C22H34O2	330.2552	331.2625	-0.7
3	19-Acetoxy-9(11),15-pimaradiene	C22H34O2	330.2555	331.2628	-0.4
4	19-Acetoxy-9(11),15-pimaradiene	C22H34O2	330.2562	331.2635	0.3
5	19-Acetoxy-9(11),15-pimaradiene	C22H34O2	330.2562	331.2635	0.4
6	2,4,7-Trimethoxy phenanthrene	C17H16O3	268.1108	269.1181	0.9
7	2,6-Dimethylaniline	C8H11N	121.0889	122.0962	-0.2
8	2,6-Dimethylaniline	C8H11N	121.0890	122.0963	-0.2
9	2-Carboxymethyl-3-prenyl-2,3-epoxy-1,4-naphthpeuinone	C17H16O4	284.1044	285.1117	-0.5
10	7-O-(3,3-Dimethylallyl)-scopoletin	C15H16O4	260.1057	261.1130	0.8
11	7-O-(3,3-Dimethylallyl)-scopoletin	C15H16O4	260.1061	261.1134	1.3
12	Cistanoside D	C31H40O15	652.2350	653.2422	-1.8
13	Eleutherazine B	C22H36N4O8	484.2529	485.2601	-0.5
14	Flavokawain B	C17H16O4	284.1041	285.1114	-0.8
15	Glucosinalbin	C14H19NO10S2	425.0466	426.0539	1.6
16	Hispidin	C13H10O5	246.0540	247.0612	1.1
17	Nuezhenidic acid	C17H24O14	452.1171	453.1244	0.5
18	Periplocoside C	C49H76O16	920.5113	921.5186	-2.0
19	Periplocoside M	C34H52O9	604.3614	605.3687	0.3
20	Phytolaccagenin	C31H48O7	532.3407	533.3479	0.6
21	Toosendanin_1	C30H38O11	574.2412	575.2485	-0.2
22	Toosendanin_1	C30H38O11	574.2424	575.2497	1.0
23	Yadanzioside A	C32H44O16	684.2610	685.2683	-2.0
24	Yemuoside YM6	C31H42O16	670.2468	671.2541	-0.5

.	Component name	Formula	Mass error (ppm)	Expected RT (min)	Observed RT (min)
1	1,7-Diphenyl-5-hydroxy-4,6-heptadien-3-one	C19H18O2	3.0		18.33
2	19-Acetoxy-9(11),15-pimaradiene	C22H34O2	-2.1		19.80
3	19-Acetoxy-9(11),15-pimaradiene	C22H34O2	-1.2		20.39
4	19-Acetoxy-9(11),15-pimaradiene	C22H34O2	0.9		19.16
5	19-Acetoxy-9(11),15-pimaradiene	C22H34O2	1.1		21.85
6	2,4,7-Trimethoxy phenanthrene	C17H16O3	3.3		15.75
7	2,6-Dimethylaniline	C8H11N	-1.8		25.48
8	2,6-Dimethylaniline	C8H11N	-1.3		26.44

.	Component name	Formula	Mass error (ppm)	Expected RT (min)	Observed RT (min)
9	2-Carboxymethyl-3-prenyl-2,3-epoxy-1,4-naphthpeuinone	C17H16O4	-1.6		16.25
10	7-O-(3,3-Dimethylallyl)-scopoletin	C15H16O4	3.2		8.52
11	7-O-(3,3-Dimethylallyl)-scopoletin	C15H16O4	4.8		8.01
12	Cistanoside D	C31H40O15	-2.7		16.94
13	Eleutherazine B	C22H36N4O8	-0.9		20.89
14	Flavokawain B	C17H16O4	-2.7		11.23
15	Glucosinalbin	C14H19NO10S2	3.7		11.14
16	Hispidin	C13H10O5	4.6		1.25
17	Nuezhenidic acid	C17H24O14	1.0		18.15
18	Periplocoside C	C49H76O16	-2.2		20.65
19	Periplocoside M	C34H52O9	0.5		22.36
20	Phytolaccagenin	C31H48O7	1.2		23.45
21	Toosendanin_1	C30H38O11	-0.4		22.01
22	Toosendanin_1	C30H38O11	1.8		22.36
23	Yadanzioside A	C32H44O16	-2.9		18.32
24	Yemuoside YM6	C31H42O16	-0.7		17.39

.	Component name	Formula	Detector counts	Response	Adducts
1	1,7-Diphenyl-5-hydroxy-4,6-heptadien-3-one	C19H18O2	8880	7516	+H
2	19-Acetoxy-9(11),15-pimaradiene	C22H34O2	12797	10678	+H
3	19-Acetoxy-9(11),15-pimaradiene	C22H34O2	17663	14606	+H
4	19-Acetoxy-9(11),15-pimaradiene	C22H34O2	192390	162655	+H
5	19-Acetoxy-9(11),15-pimaradiene	C22H34O2	10704	8804	+H
6	2,4,7-Trimethoxy phenanthrene	C17H16O3	7152	5979	+H
7	2,6-Dimethylaniline	C8H11N	29480	28314	+H
8	2,6-Dimethylaniline	C8H11N	19932	18773	+H
9	2-Carboxymethyl-3-prenyl-2,3-epoxy-1,4-naphthpeuinone	C17H16O4	7132	5927	+H
10	7-O-(3,3-Dimethylallyl)-scopoletin	C15H16O4	7563	6553	+H
11	7-O-(3,3-Dimethylallyl)-scopoletin	C15H16O4	6241	5549	+H
12	Cistanoside D	C31H40O15	33709	19033	+H
13	Eleutherazine B	C22H36N4O8	39483	28666	+H
14	Flavokawain B	C17H16O4	23241	20391	+H
15	Glucosinalbin	C14H19NO10S2	17053	8464	+H
16	Hispidin	C13H10O5	18845	17437	+H
17	Nuezhenidic acid	C17H24O14	29245	23291	+H
18	Periplocoside C	C49H76O16	68113	36455	+H
19	Periplocoside M	C34H52O9	231956	162403	+H
20	Phytolaccagenin	C31H48O7	36343	26495	+H
21	Toosendanin_1	C30H38O11	38174	27802	+H
22	Toosendanin_1	C30H38O11	72933	54898	+H
23	Yadanzioside A	C32H44O16	64629	39426	+H
24	Yemuoside YM6	C31H42O16	95517	68157	+H

Component name: 1,7-Diphenyl-5-hydroxy-4,6-heptadien-3-one

Item name: La_VT_08052026

Item name: La_VT_Ea_10042026
Channel name: 1,7-Diphenyl-5-hydroxy-4,6-heptadien-3-one [+H] : (45.5 PPM) 279.1388

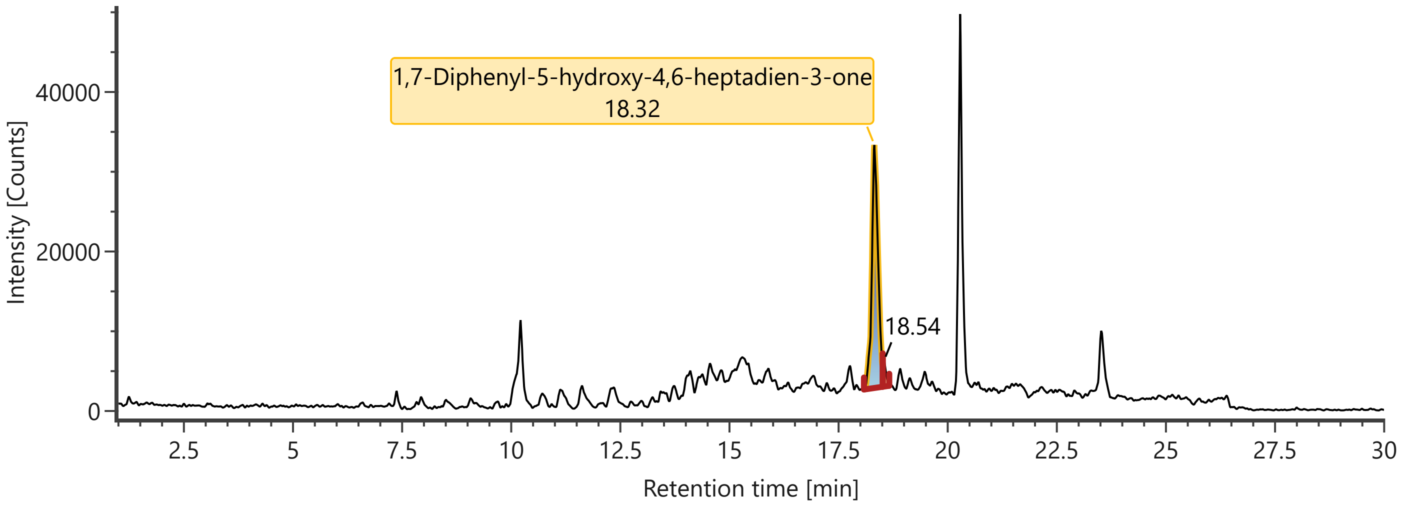

Item name: La_VT_Ea_10042026
Item description:

Channel name: Low energy : Time 18.3327 +/- 0.0525 minutes

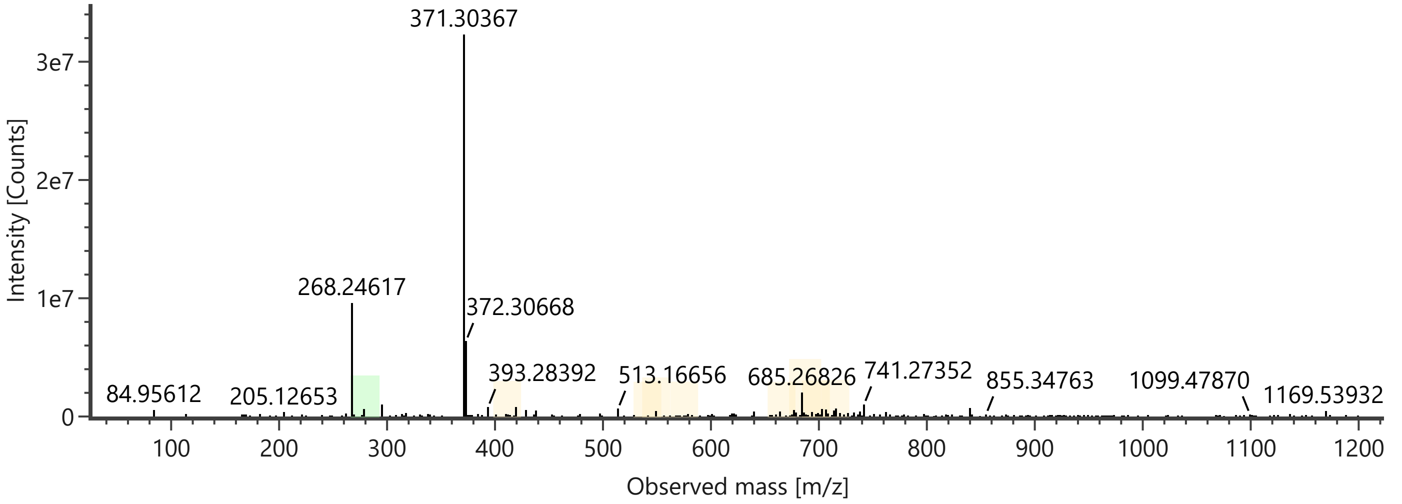

Item name: La_VT_08052026

Item name: La_VT_Ea_10042026
Item description:

Channel name: High energy : Time 18.3327 +/- 0.0525 minutes

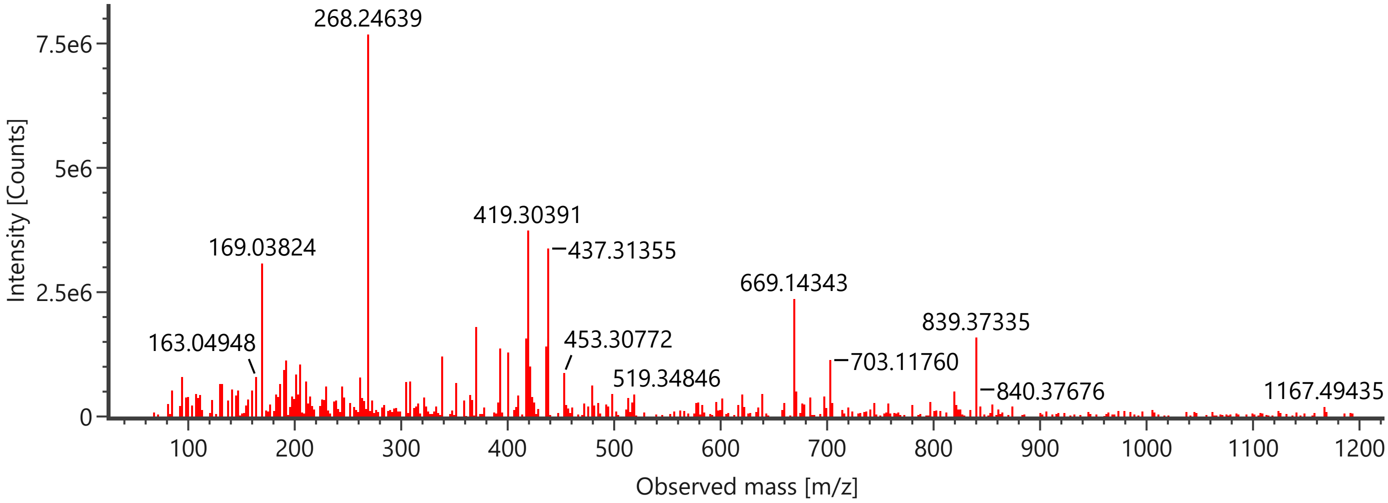

Component name: 19-Acetoxy-9(11),15-pimaradiene

Item name: La_VT_Ea_10042026
Channel name: 19-Acetoxy-9(11),15-pimaradiene [+H] : (45.5 PPM) 331.2625

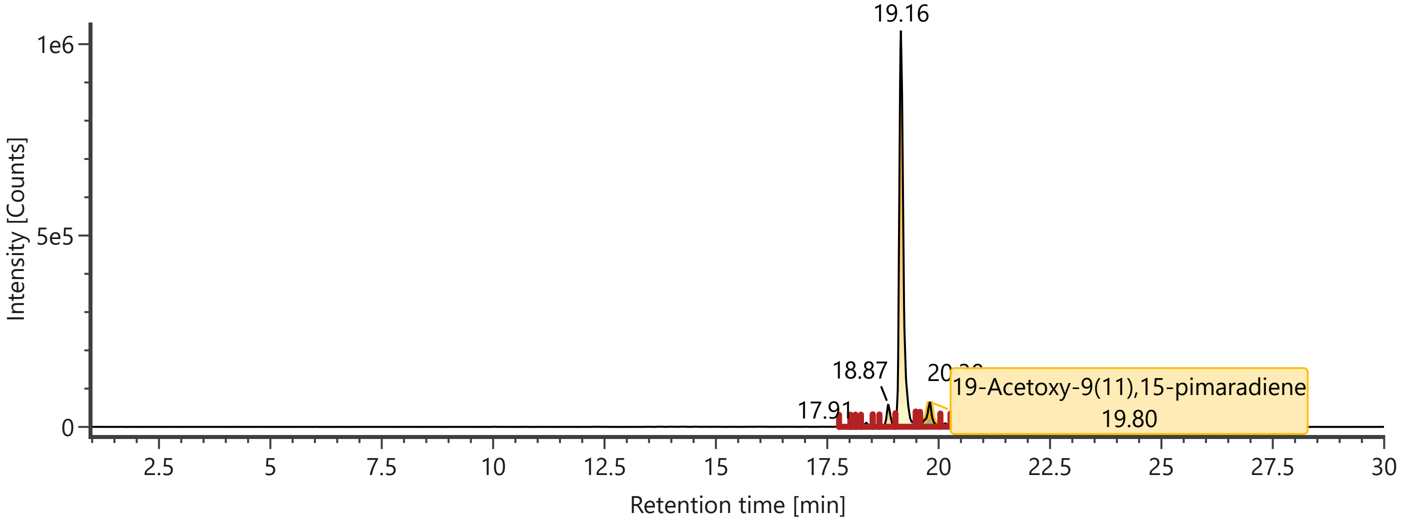

Item name: La_VT_Ea_10042026
Item description:

Channel name: Low energy : Time 19.7993 +/- 0.0525 minutes

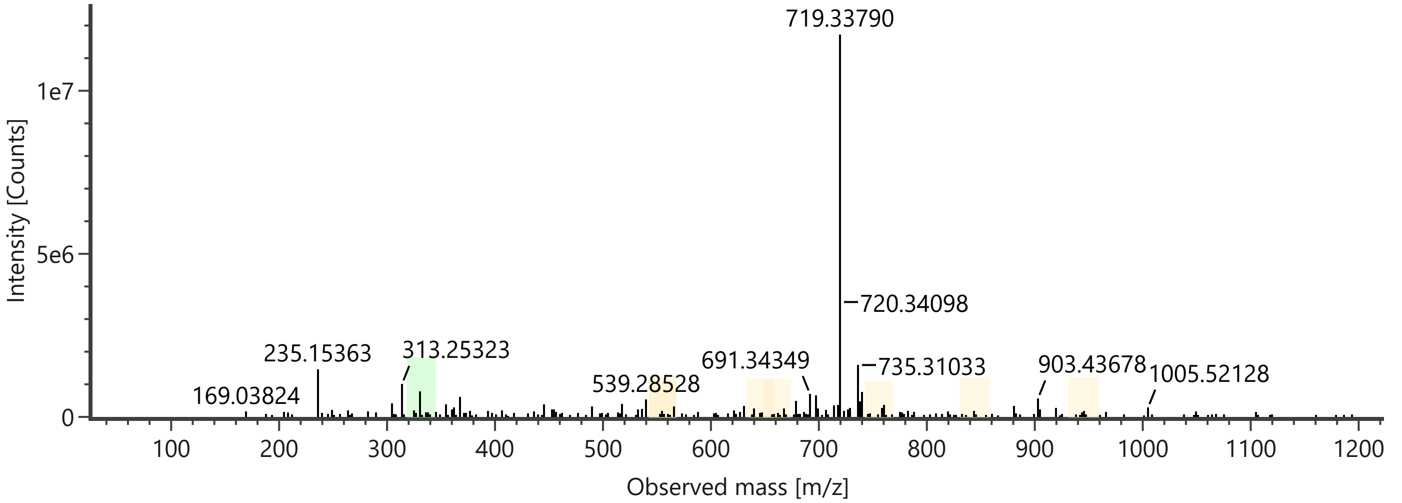

Item name: La_VT_Ea_10042026

Item description:

Channel name: High energy : Time 19.7993 +/- 0.0525 minutes

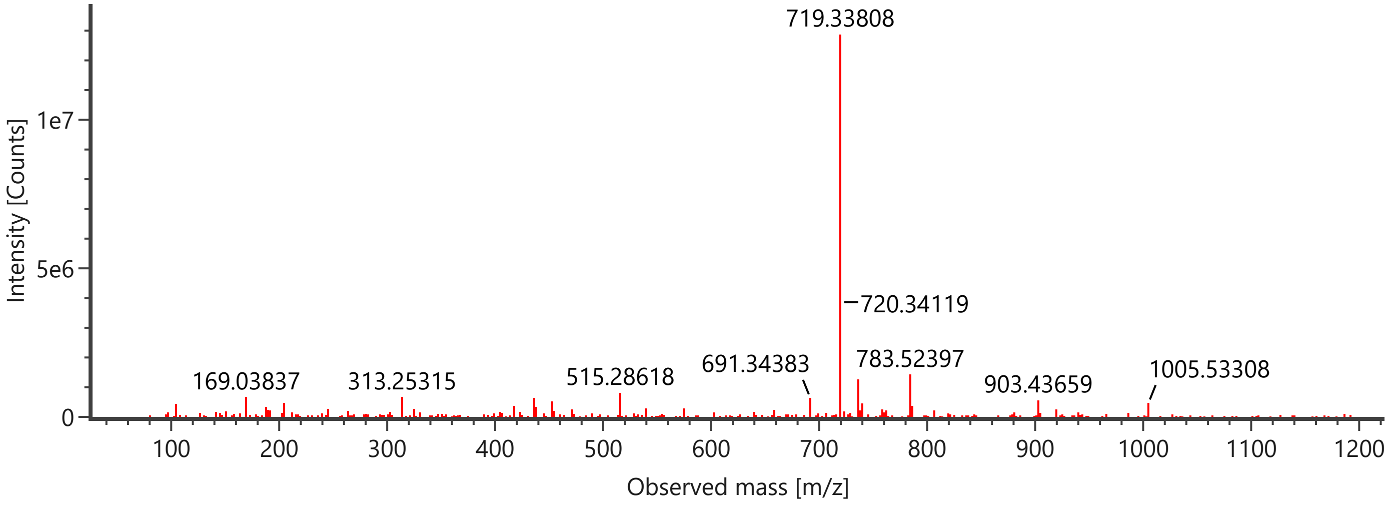

Item name: La_VT_08052026

Created by: Administrator, UNIFI

Created on: May 08, 2026

Created time: 14:44:42 SE Asia Standard Time

Component name: 19-Acetoxy-9(11),15-pimaradiene

Item name: La_VT_Ea_10042026
Channel name: 19-Acetoxy-9(11),15-pimaradiene [+H] : (45.5 PPM) 331.2628

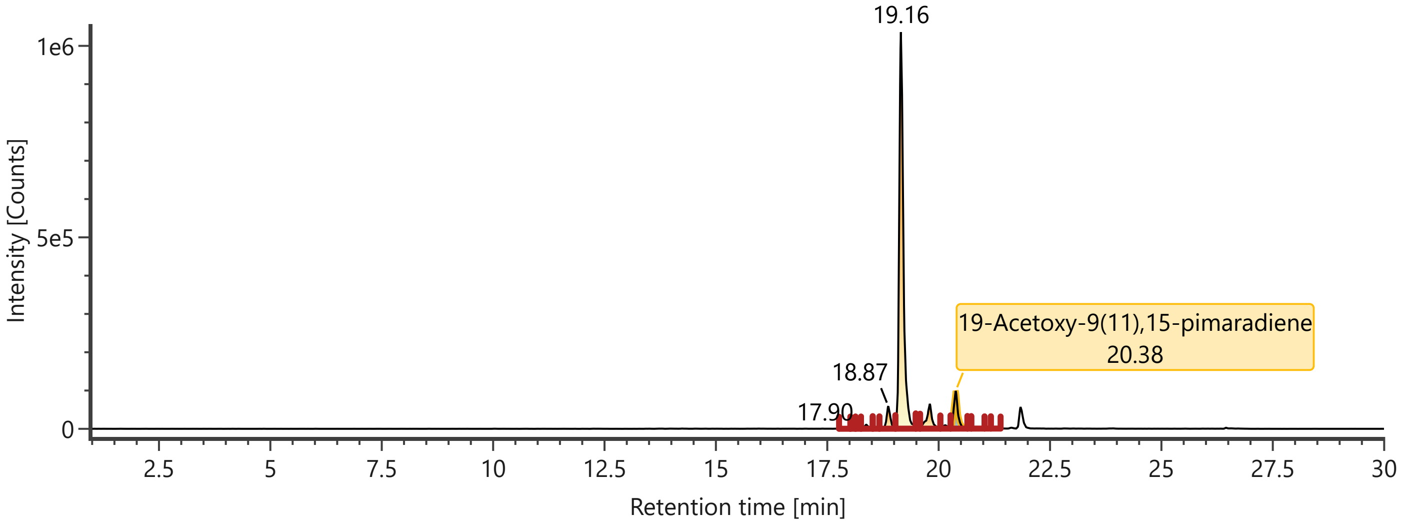

Item name: La_VT_Ea_10042026
Item description:

Channel name: Low energy : Time 20.3855 +/- 0.0525 minutes

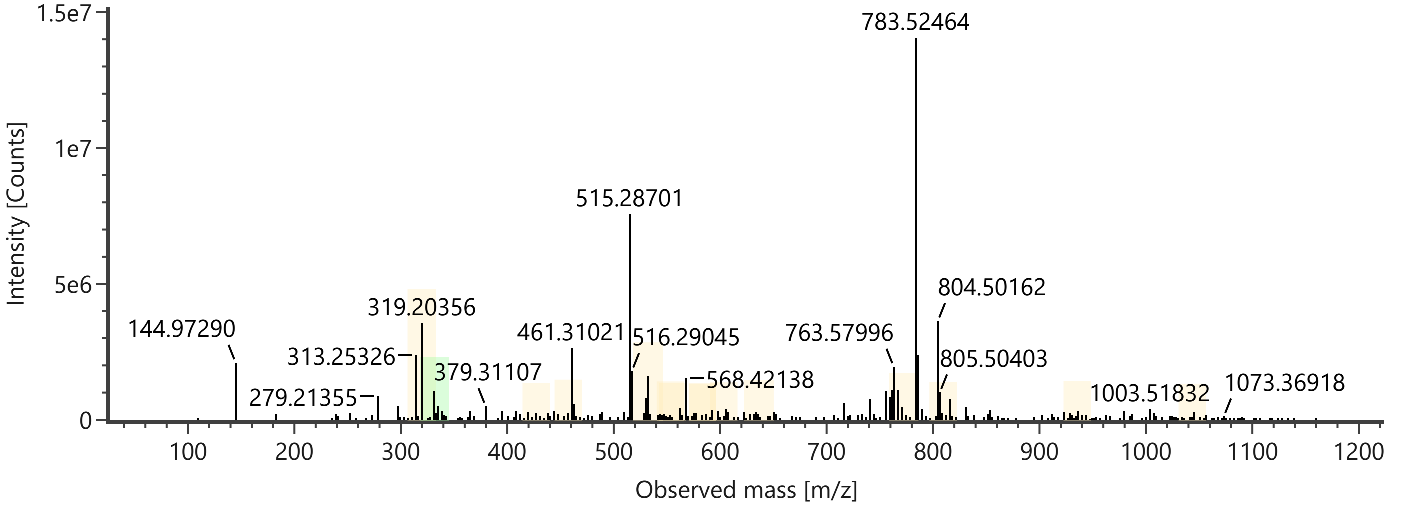

Item name: La_VT_Ea_10042026
Item description:

Channel name: High energy : Time 20.3855 +/- 0.0525 minutes

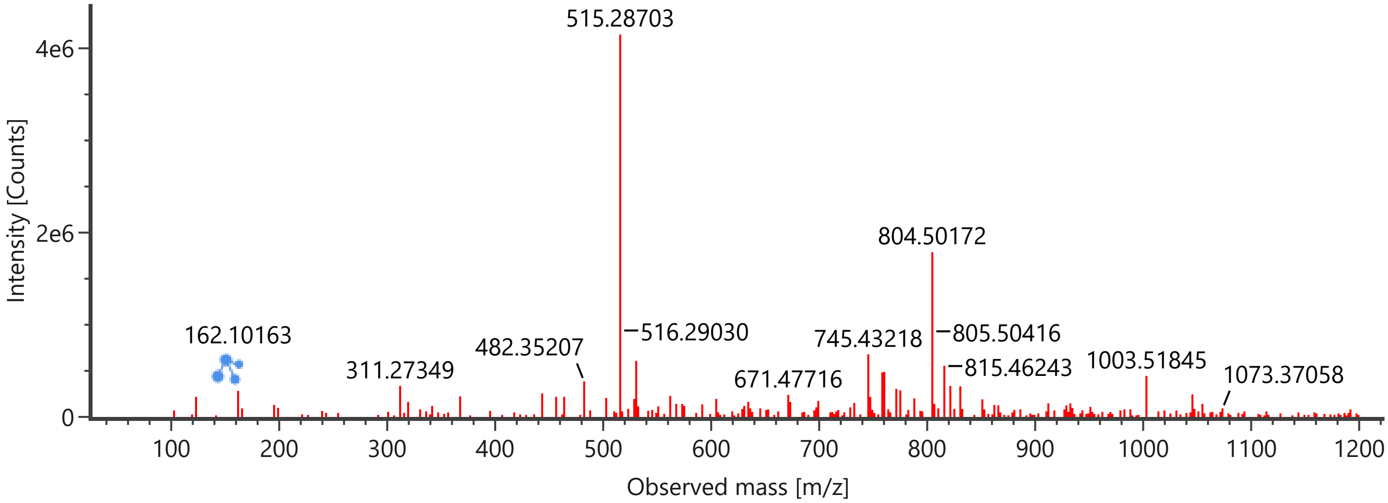

Item name: La_VT_08052026

Component name: 19-Acetoxy-9(11),15-pimaradiene

Item name: La_VT_Ea_10042026
Channel name: 19-Acetoxy-9(11),15-pimaradiene [+H] : (45.5 PPM) 331.2635

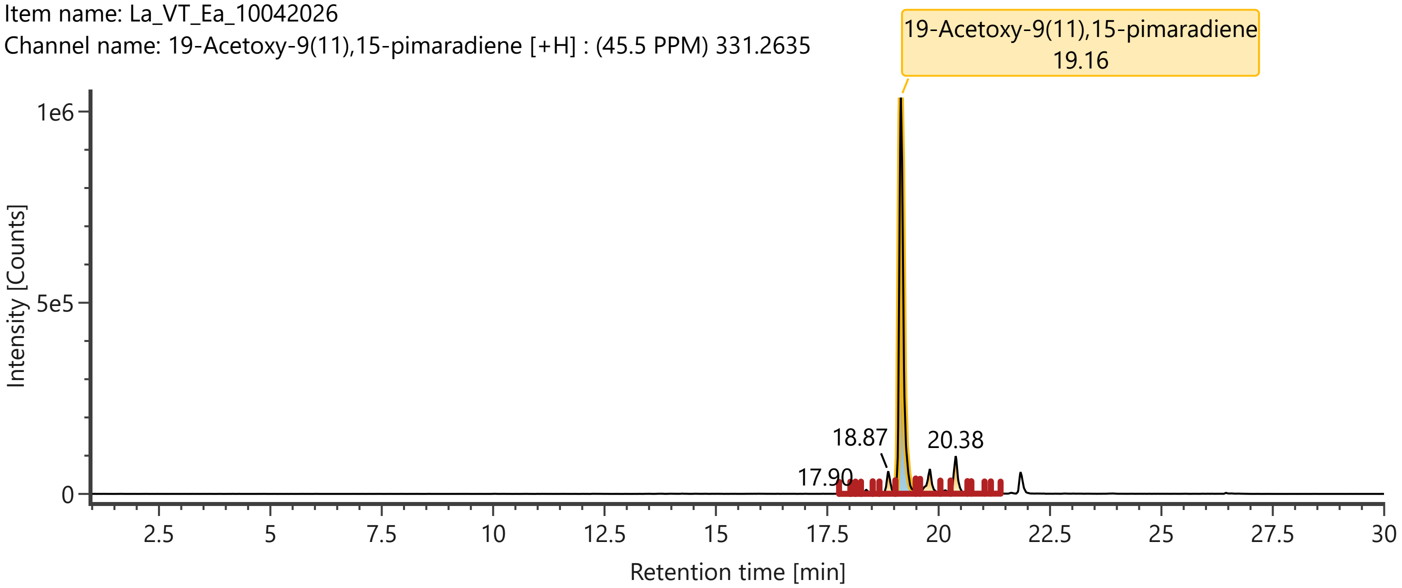

Item name: La_VT_Ea_10042026
Channel name: Low energy : Time 19.1611 +/- 0.0525 minutes
Item description:

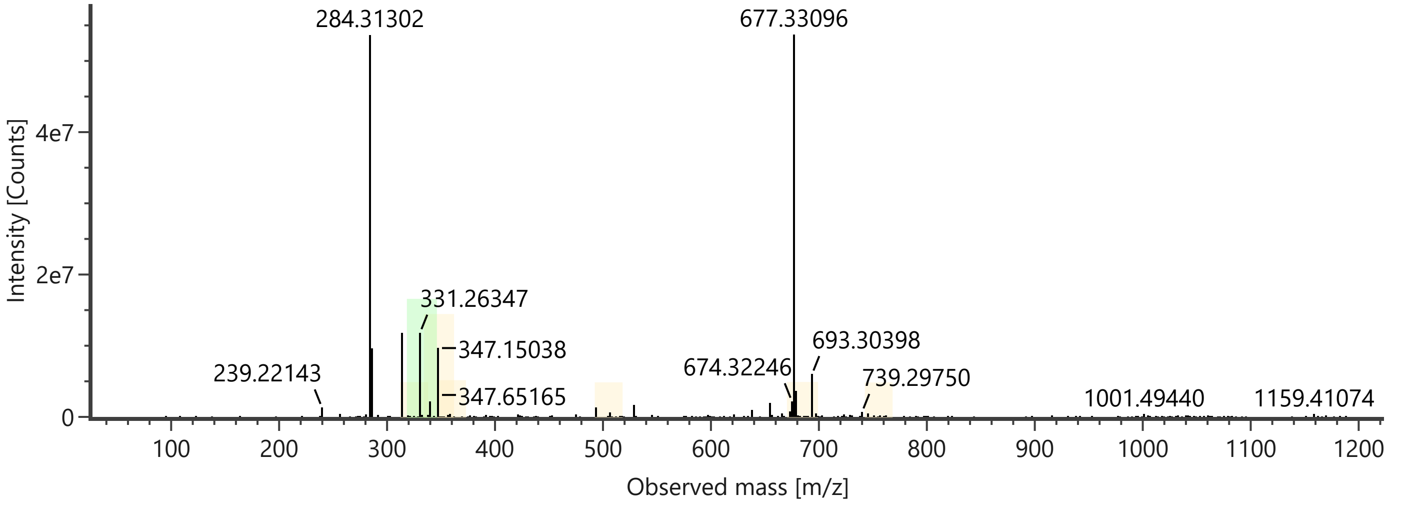

Item name: La_VT_Ea_10042026
Item description:

Channel name: High energy : Time 19.1611 +/- 0.0525 minutes

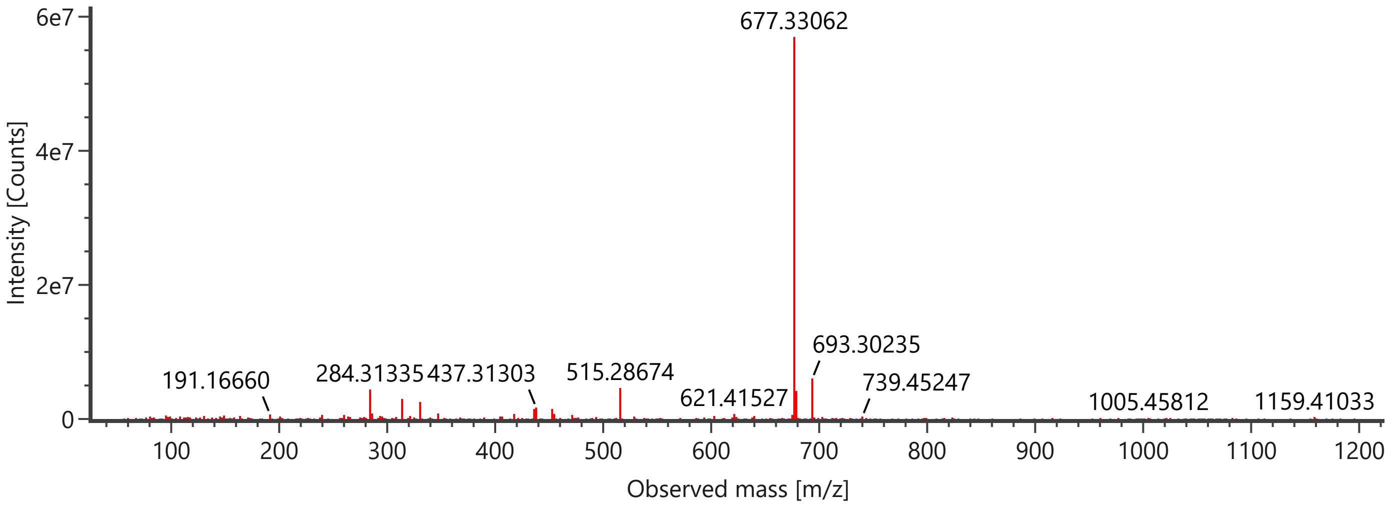

Item name: La_VT_08052026

Component name: 19-Acetoxy-9(11),15-pimaradiene

Item name: La_VT_Ea_10042026
Channel name: 19-Acetoxy-9(11),15-pimaradiene [+H] : (45.5 PPM) 331.2635

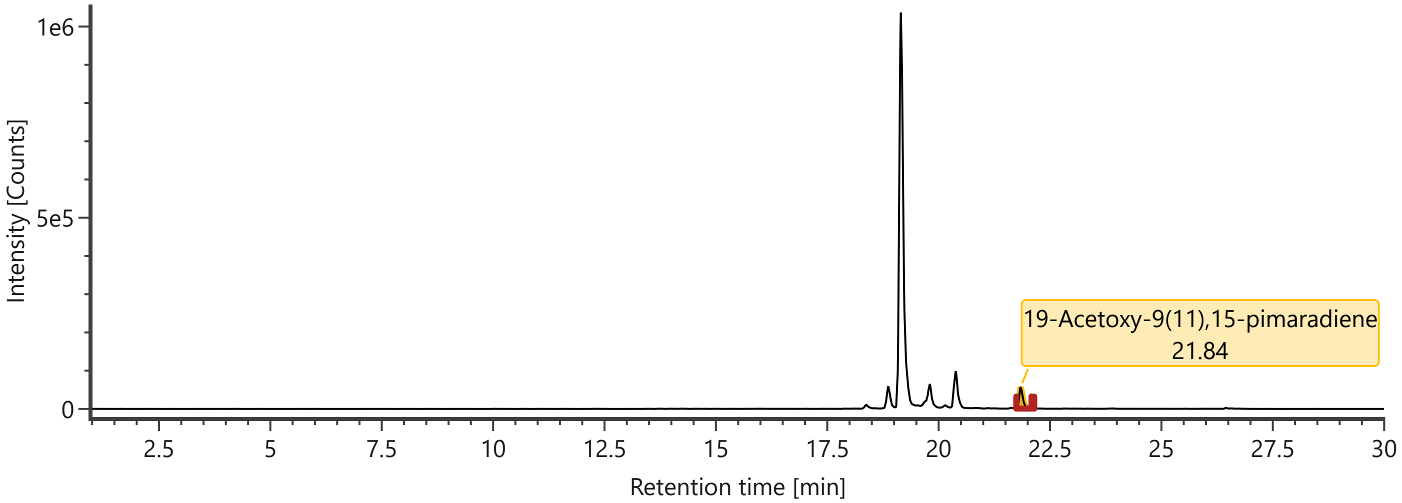

Item name: La_VT_Ea_10042026
Channel name: Low energy : Time 21.8515 +/- 0.0525 minutes
Item description:

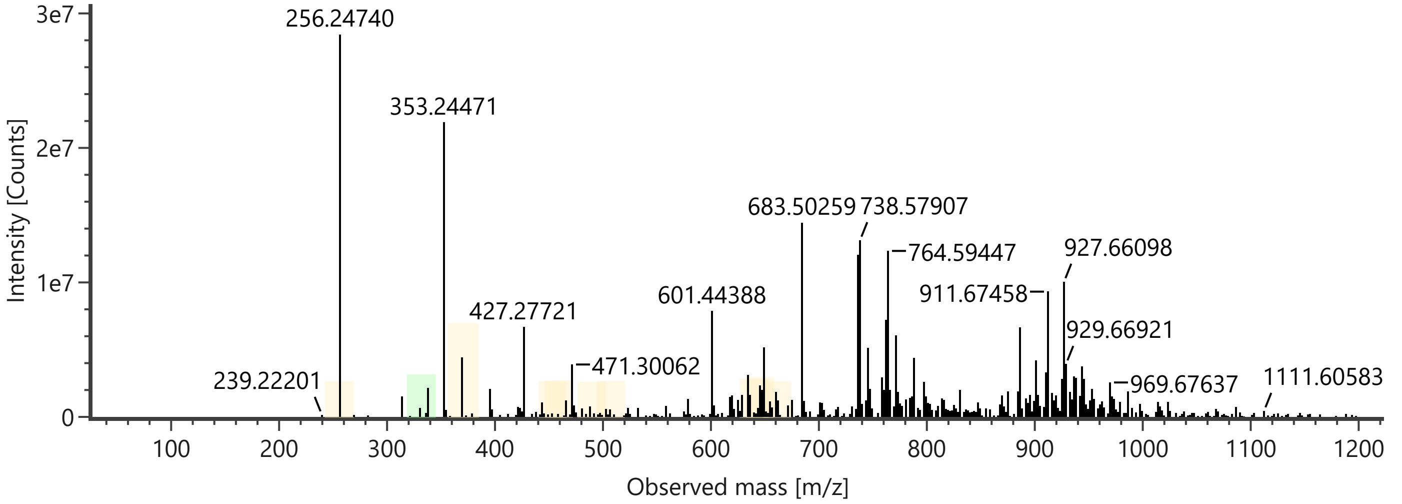

Item name: La_VT_Ea_10042026
Item description:

Channel name: High energy : Time 21.8515 +/- 0.0525 minutes

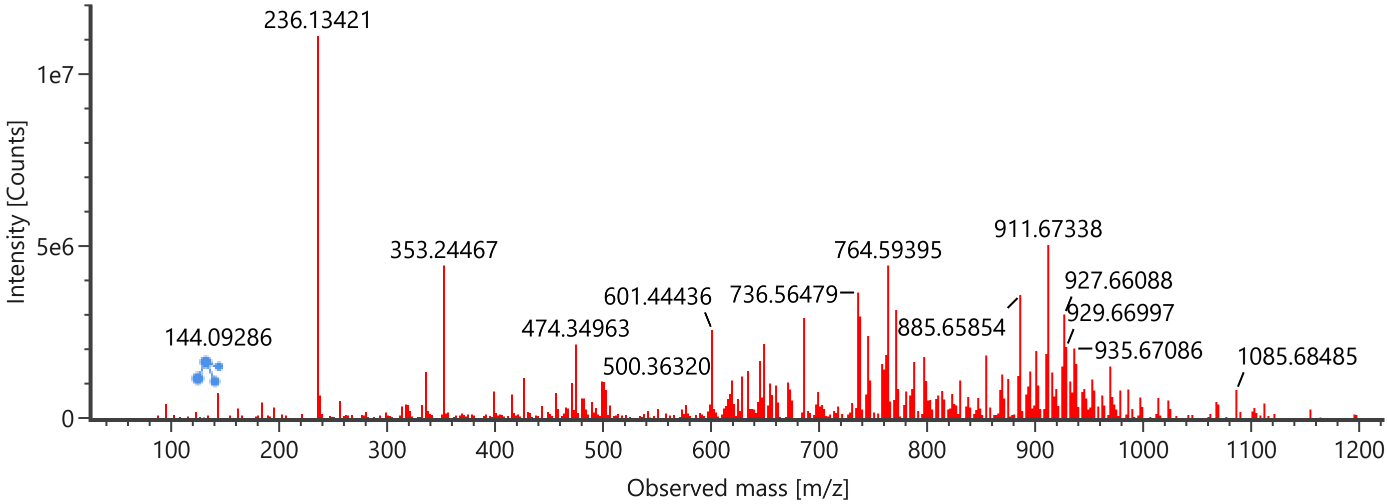

Item name: La_VT_08052026

Component name: 2,4,7-Trimethoxy phenanthrene

Item name: La_VT_Ea_10042026
Channel name: 2,4,7-Trimethoxy phenanthrene [+H] : (45.5 PPM) 269.1181

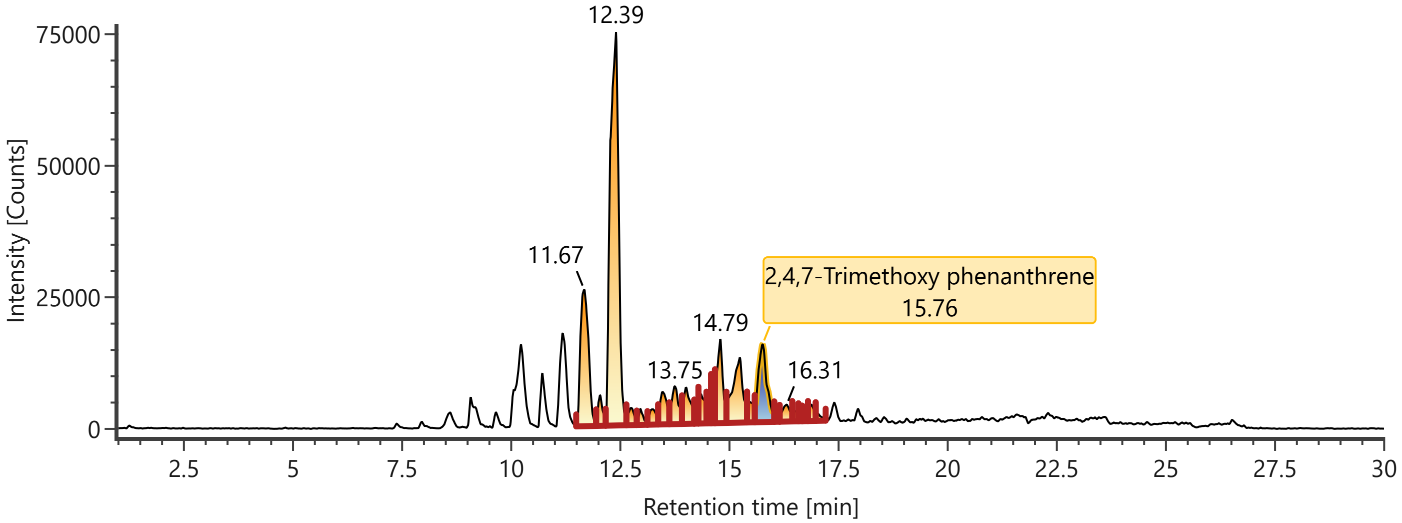

Item name: La_VT_Ea_10042026
Item description:

Channel name: Low energy : Time 15.7488 +/- 0.0525 minutes

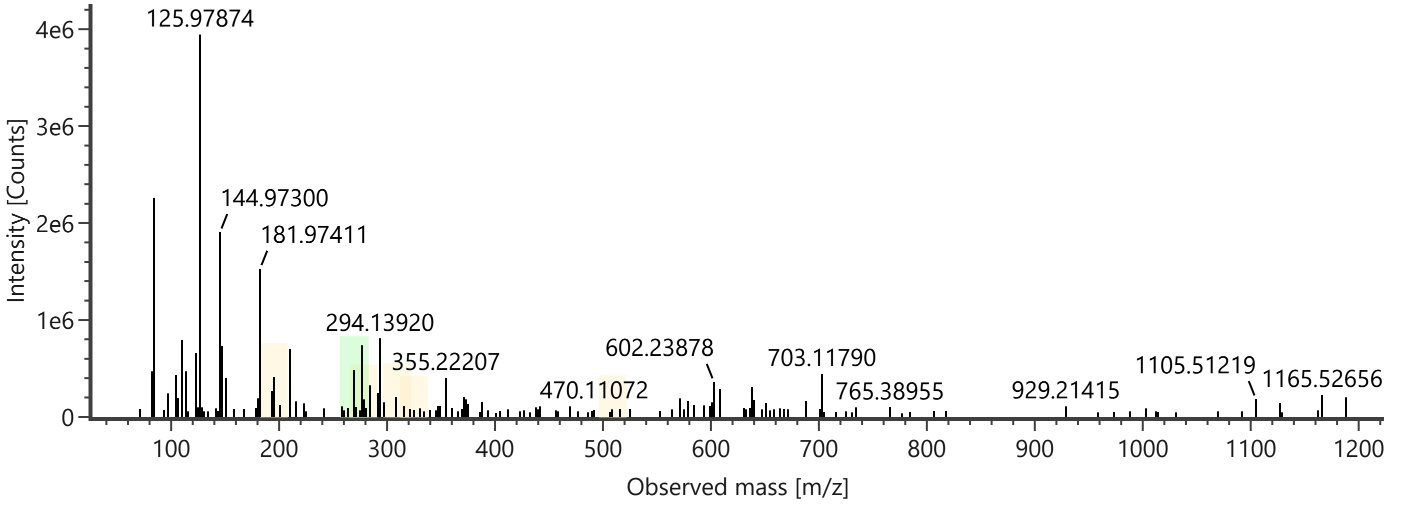

Item name: La_VT_Ea_10042026
Item description:

Channel name: High energy : Time 15.7488 +/- 0.0525 minutes

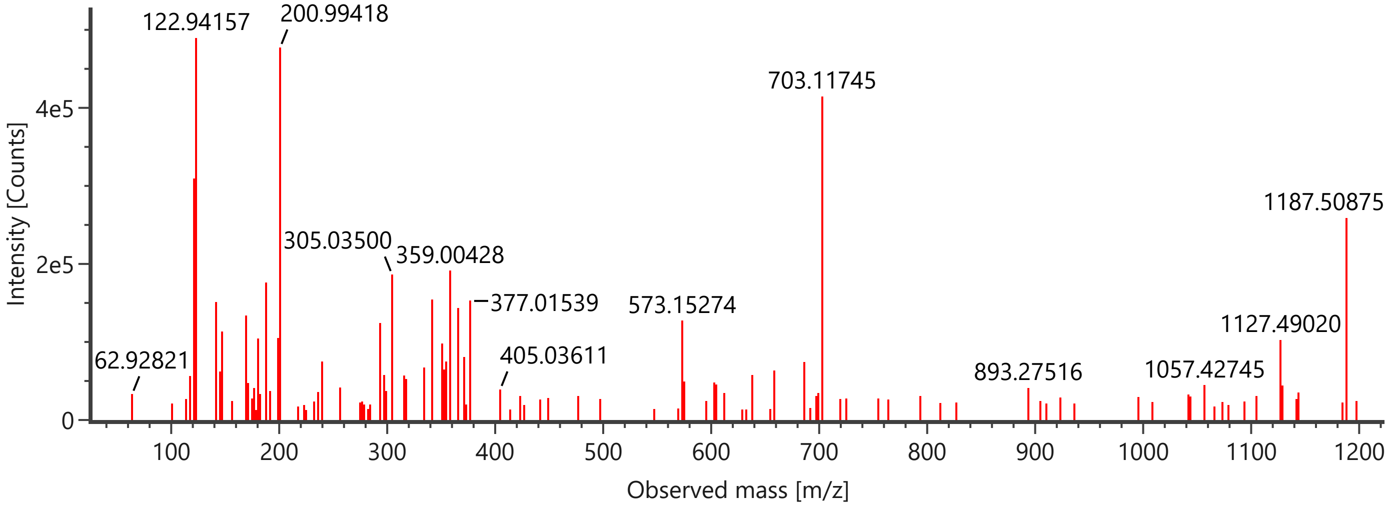

Item name: La_VT_08052026

Component name: 2,6-Dimethylaniline

Item name: La_VT_Ea_10042026
Channel name: 2,6-Dimethylaniline [+H] : (45.5 PPM) 122.0962

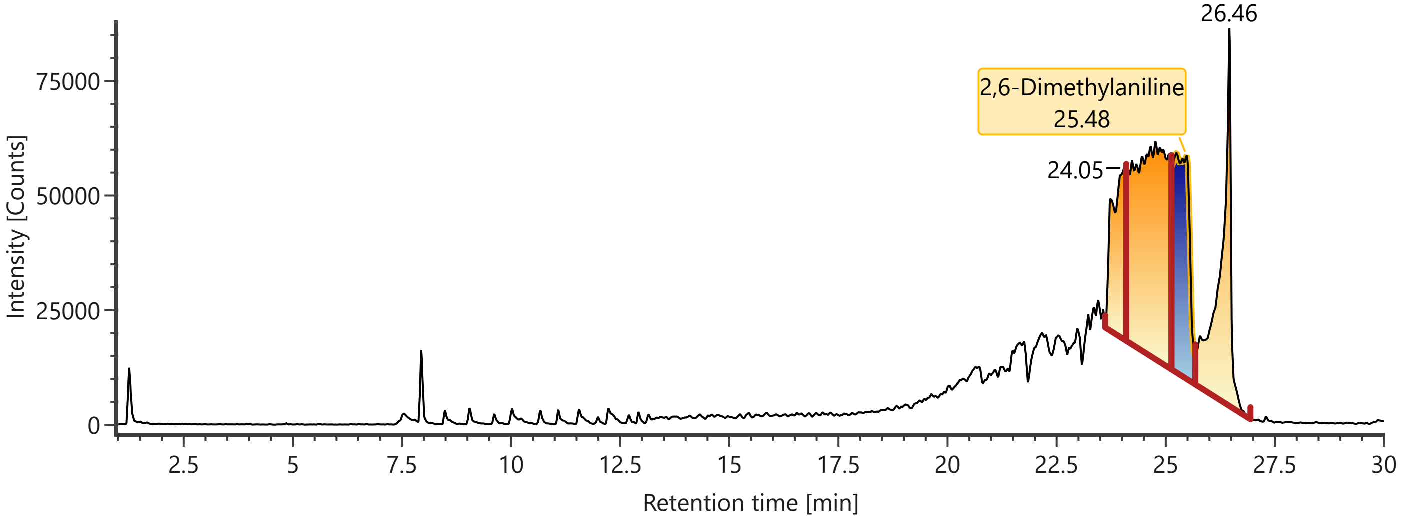

Item name: La_VT_Ea_10042026
Item description:

Channel name: Low energy : Time 25.4839 +/- 0.0525 minutes

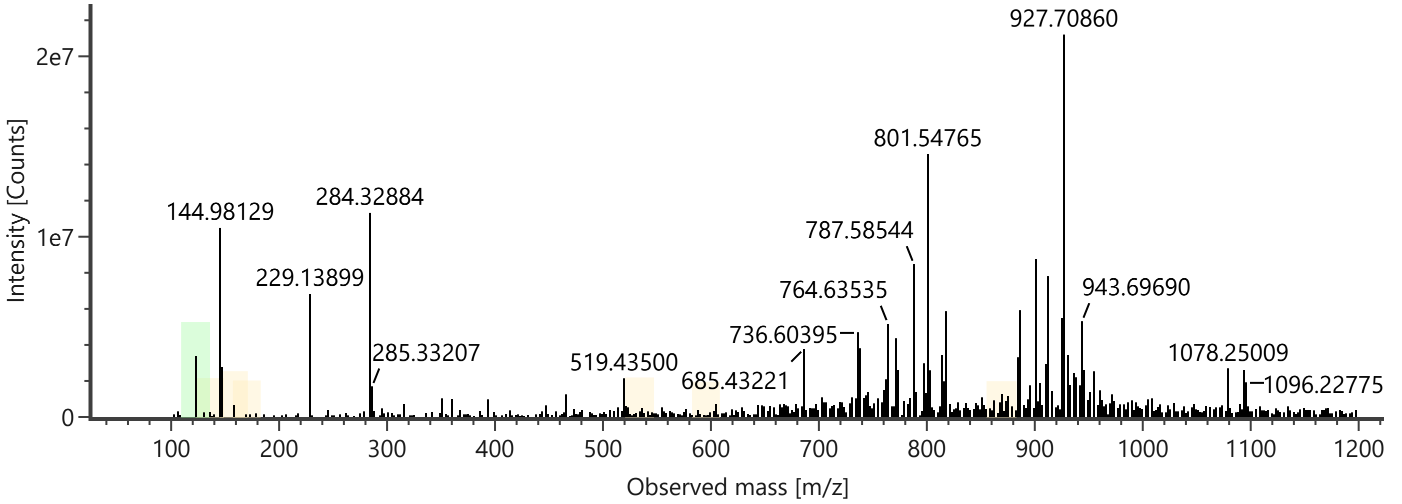

Item name: La_VT_Ea_10042026
Item description:

Channel name: High energy : Time 25.4839 +/- 0.0525 minutes

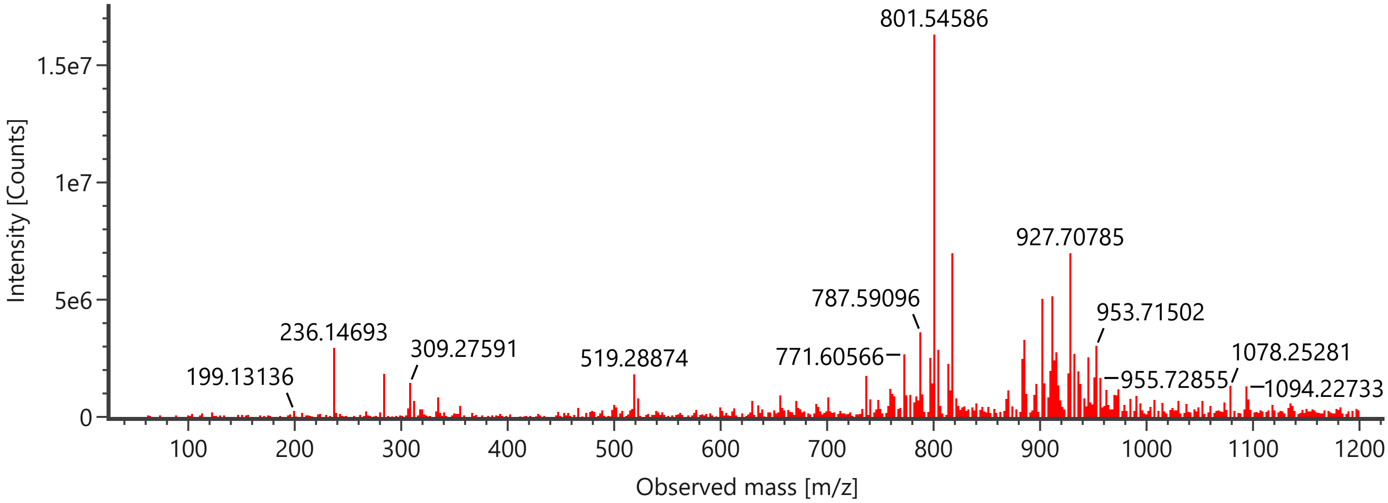

Component name: 2,6-Dimethylaniline

Item name: La_VT_Ea_10042026
Channel name: 2,6-Dimethylaniline [+H] : (45.5 PPM) 122.0963

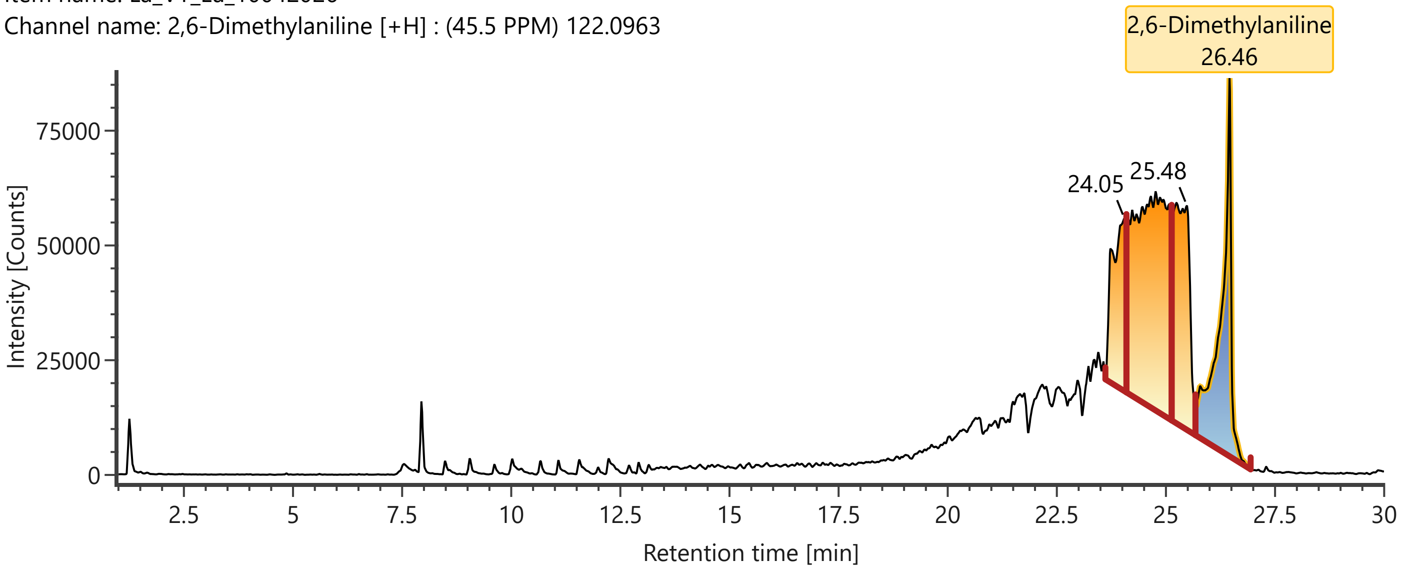

Item name: La_VT_Ea_10042026
Item description:
Channel name: Low energy : Time 26.4447 +/- 0.0525 minutes

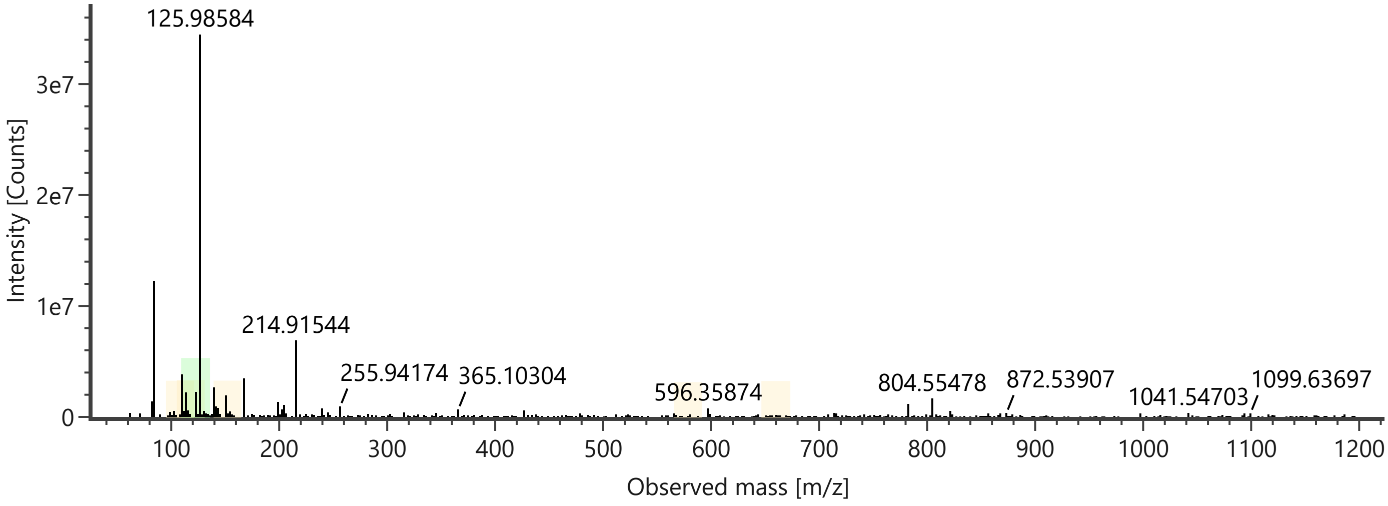

Item name: La_VT_Ea_10042026
Item description:

Channel name: High energy : Time 26.4447 +/- 0.0525 minutes

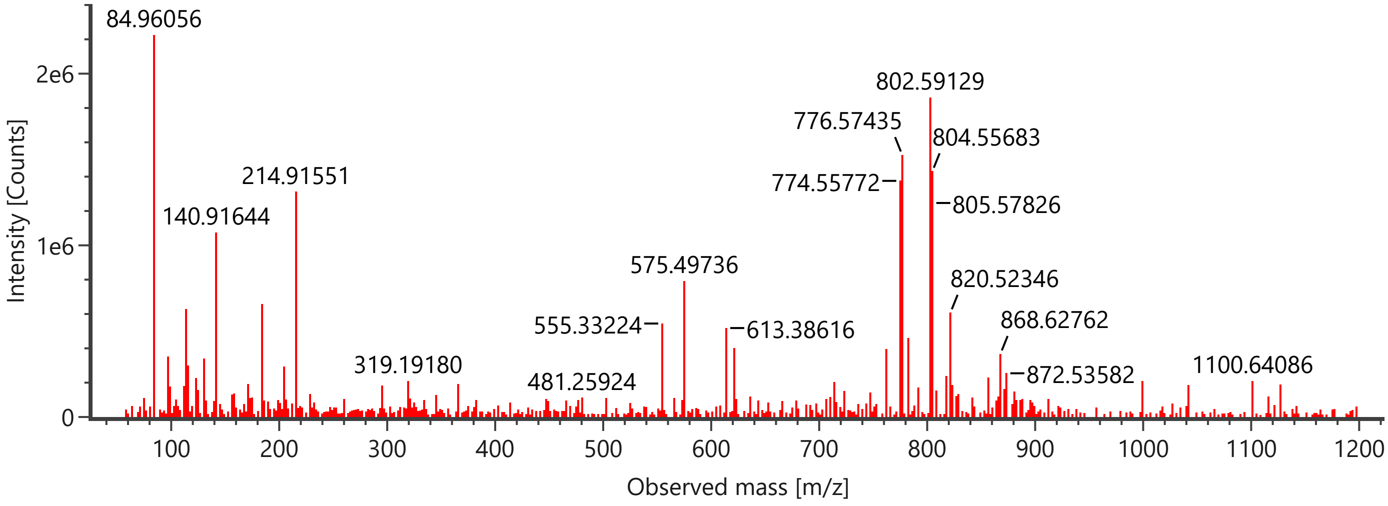

Component name: 2-Carboxymethyl-3-prenyl-2,3-epoxy-1,4-naphthpeuinone

Item name: La_VT_Ea_10042026
Channel name: 2-Carboxymethyl-3-prenyl-2,3-epoxy-1,4-naphthpeuinone [+H] : (45.5 PPM) 285.1117

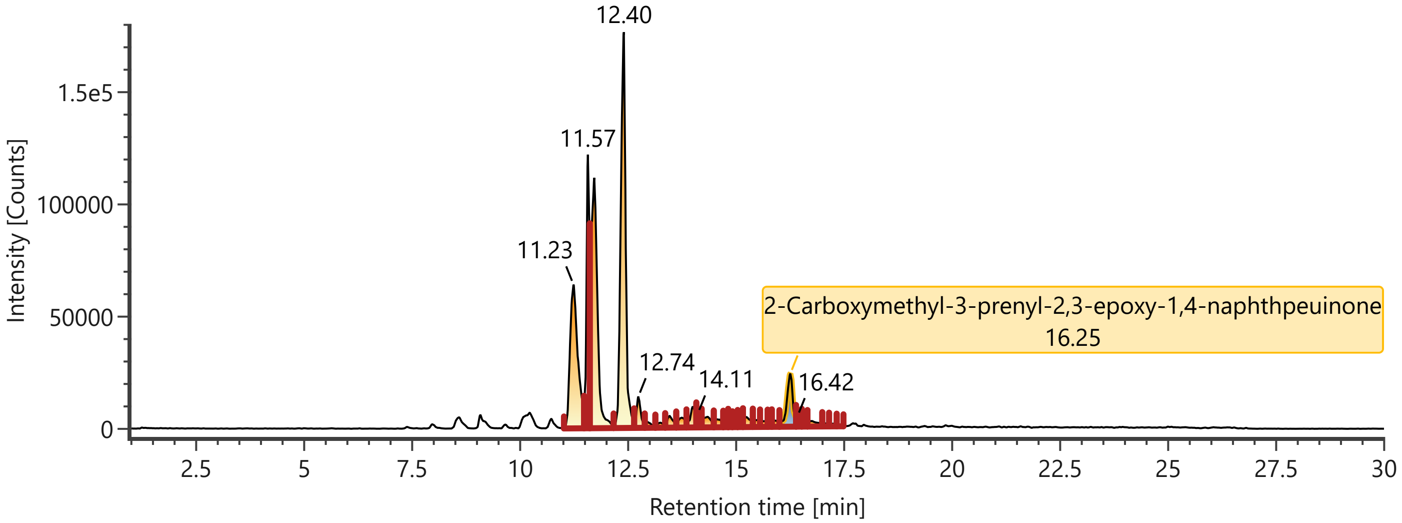

Item name: La_VT_Ea_10042026
Item description:
Channel name: Low energy : Time 16.2481 +/- 0.0525 minutes

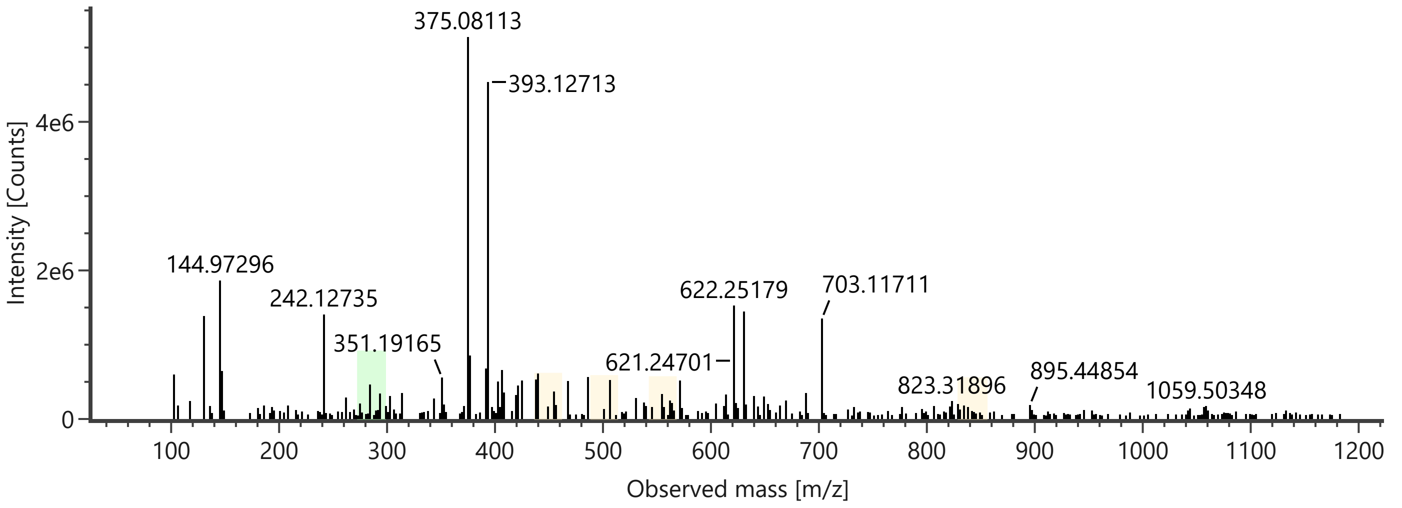

Item name: La_VT_08052026

Item name: La_VT_Ea_10042026
Item description:

Channel name: High energy : Time 16.2481 +/- 0.0525 minutes

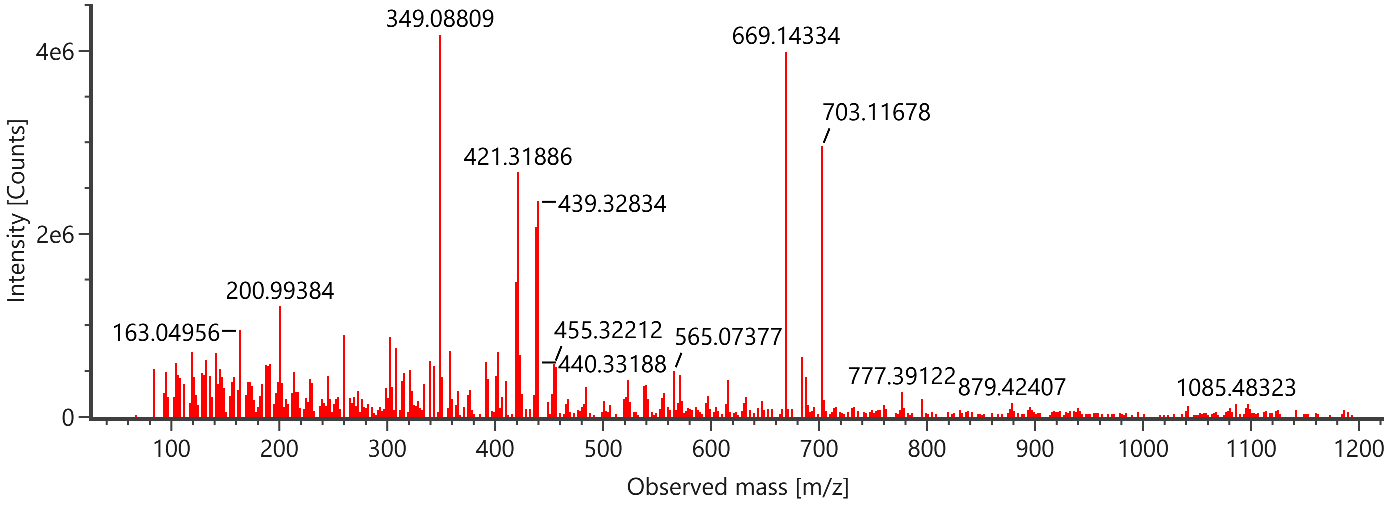

Item name: La_VT_08052026

Created by: Administrator, UNIFI

Created on: May 08, 2026

Created time: 14:44:42 SE Asia Standard Time

Component name: 7-O-(3,3-Dimethylallyl)-scopoletin

Item name: La_VT_Ea_10042026
Channel name: 7-O-(3,3-Dimethylallyl)-scopoletin [+H] : (45.5 PPM) 261.1130

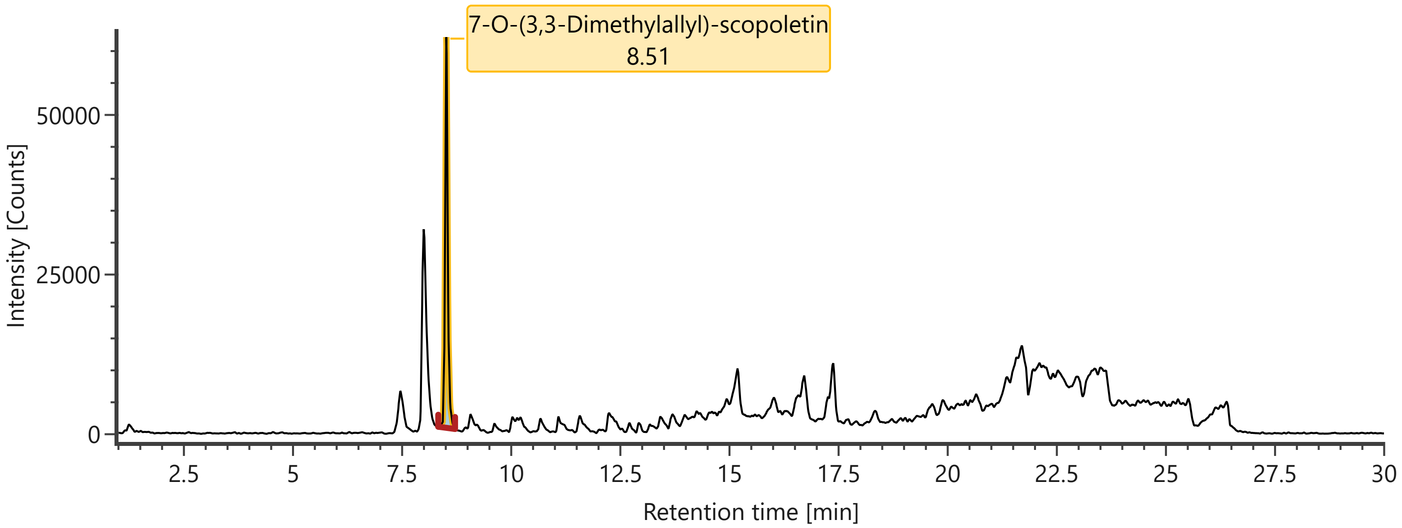

Item name: La_VT_Ea_10042026
Item description:

Channel name: Low energy : Time 8.5176 +/- 0.0525 minutes

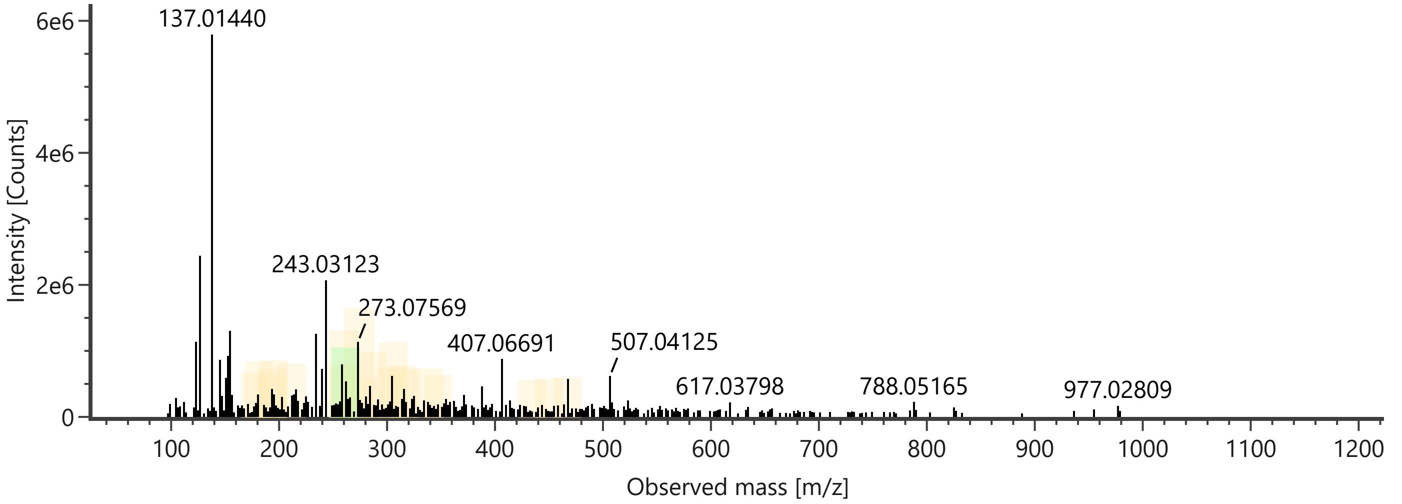

Item name: La_VT_08052026

Created by: Administrator, UNIFI

Created on: May 08, 2026

Created time: 14:44:42 SE Asia Standard Time

Item name: La_VT_Ea_10042026

Item description:

Channel name: High energy : Time 8.5176 +/- 0.0525 minutes

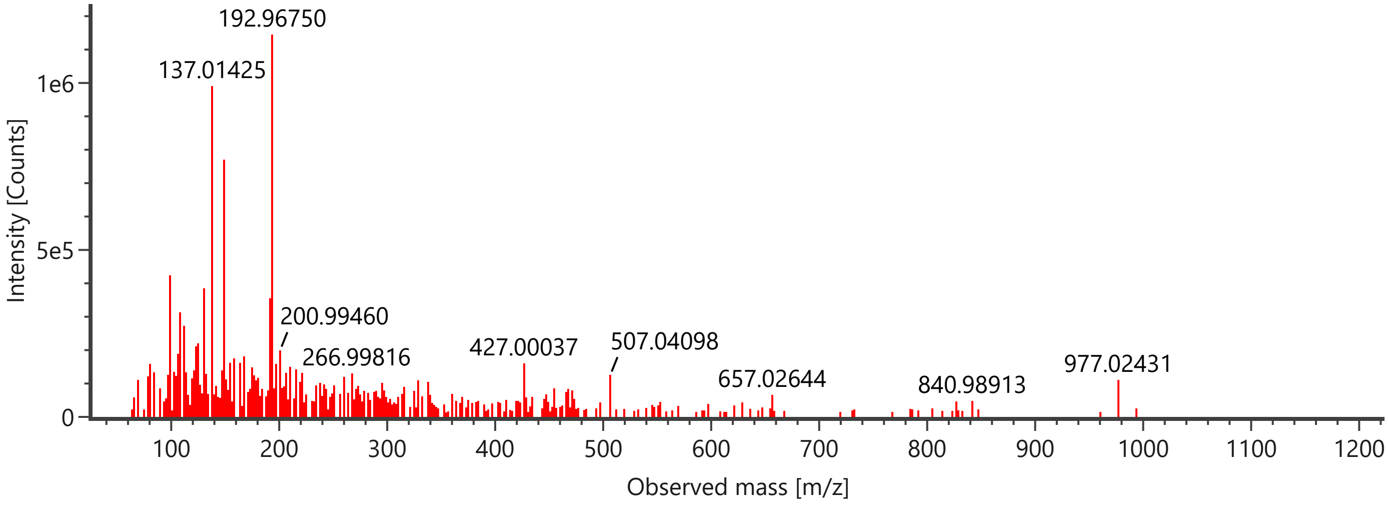

Item name: La_VT_08052026

Component name: 7-O-(3,3-Dimethylallyl)-scopoletin

Item name: La_VT_Ea_10042026
Channel name: 7-O-(3,3-Dimethylallyl)-scopoletin [+H] : (45.5 PPM) 261.1134

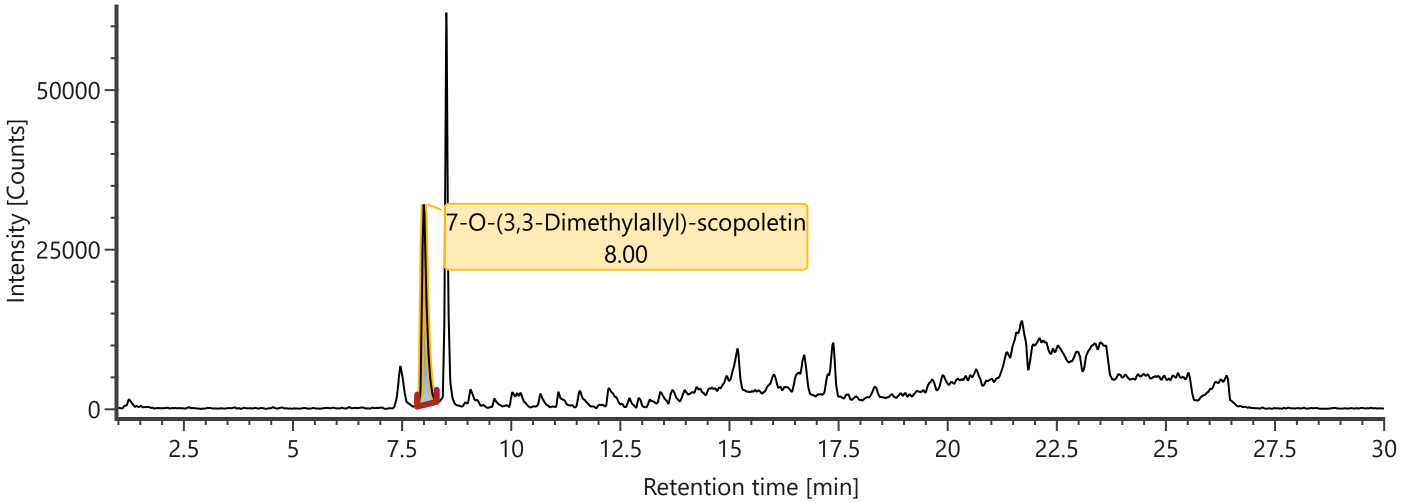

Item name: La_VT_Ea_10042026
Item description:
Channel name: Low energy : Time 8.0063 +/- 0.0525 minutes

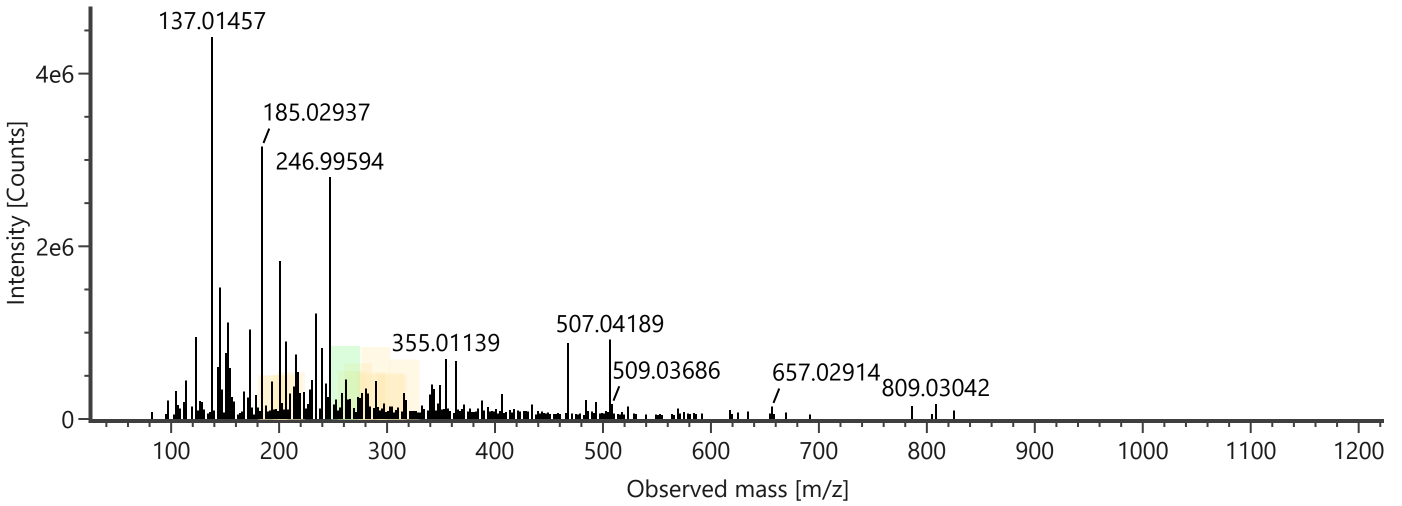

Item name: La_VT_Ea_10042026
Item description:

Channel name: High energy : Time 8.0063 +/- 0.0525 minutes

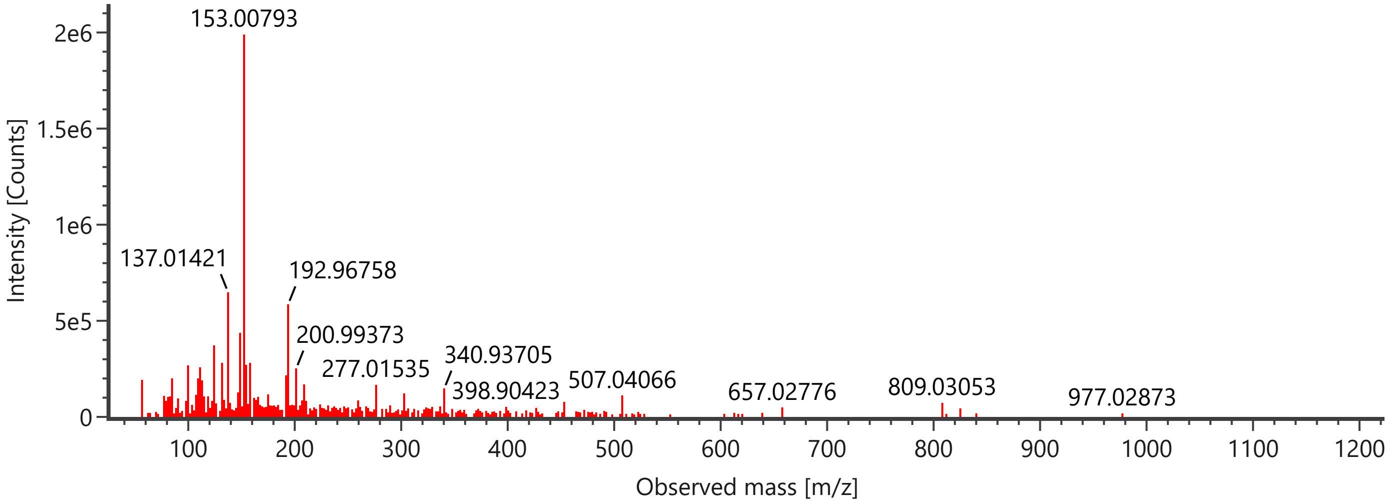

Item name: La_VT_08052026

Component name: Cistanoside D

Item name: La_VT_Ea_10042026
Channel name: Cistanoside D [+H] : (45.5 PPM) 653.2422

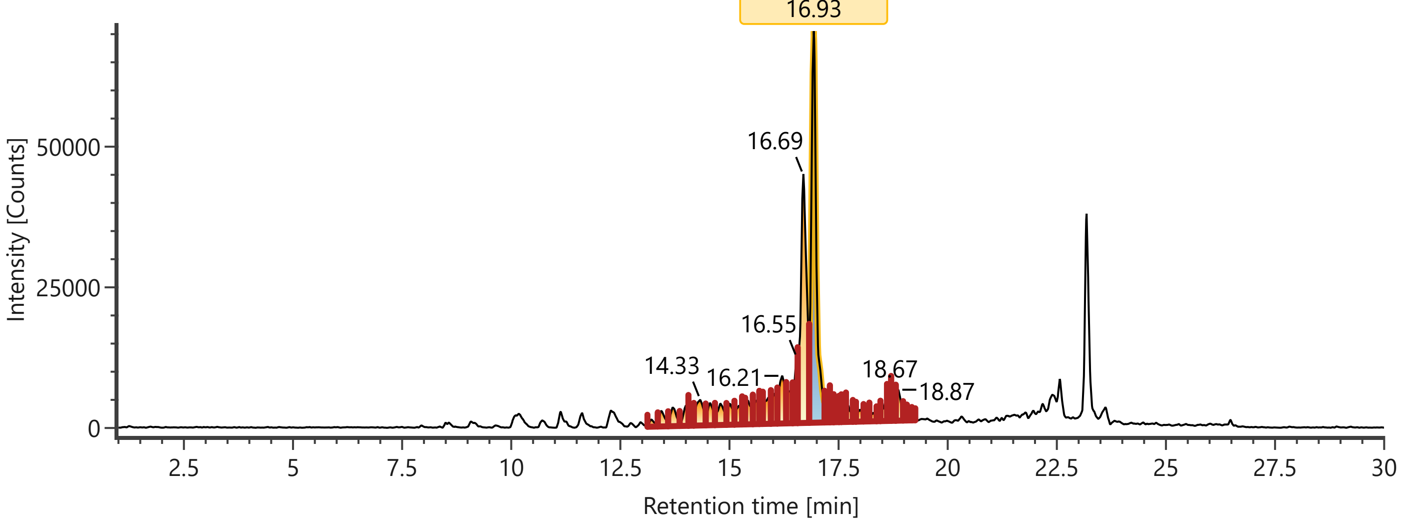

Item name: La_VT_Ea_10042026
Item description:

Channel name: Low energy : Time 16.9374 +/- 0.0525 minutes

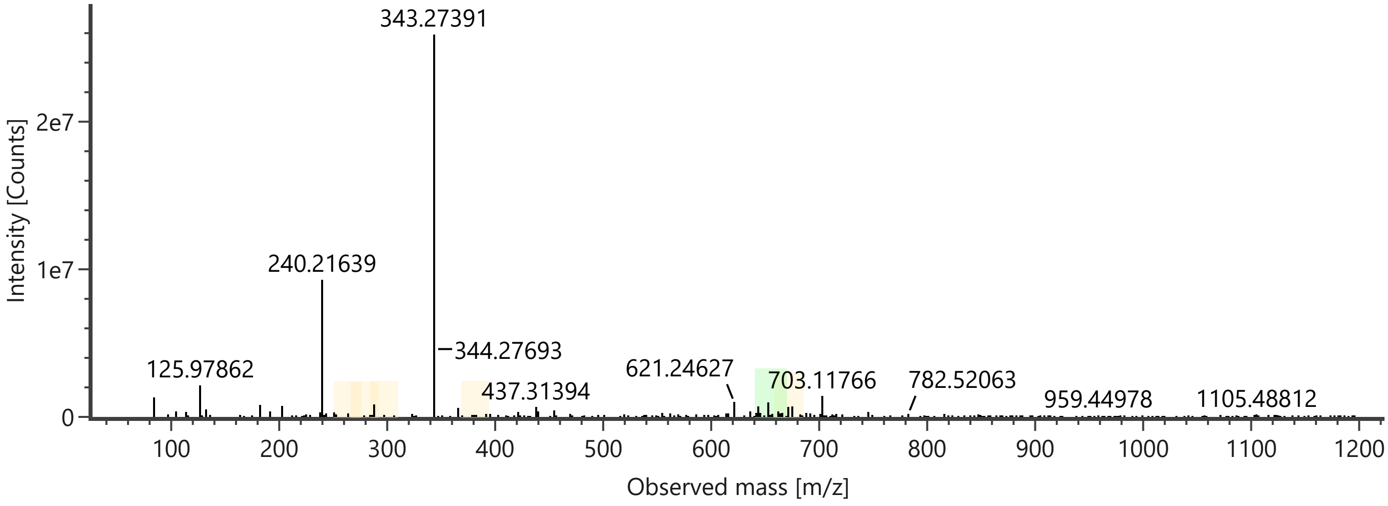

Created by: Administrator, UNIFI

Created on: May 08, 2026

Item name: La_VT_08052026

Created time: 14:44:42 SE Asia Standard Time

Item name: La_VT_Ea_10042026

Item description:

Channel name: High energy : Time 16.9374 +/- 0.0525 minutes

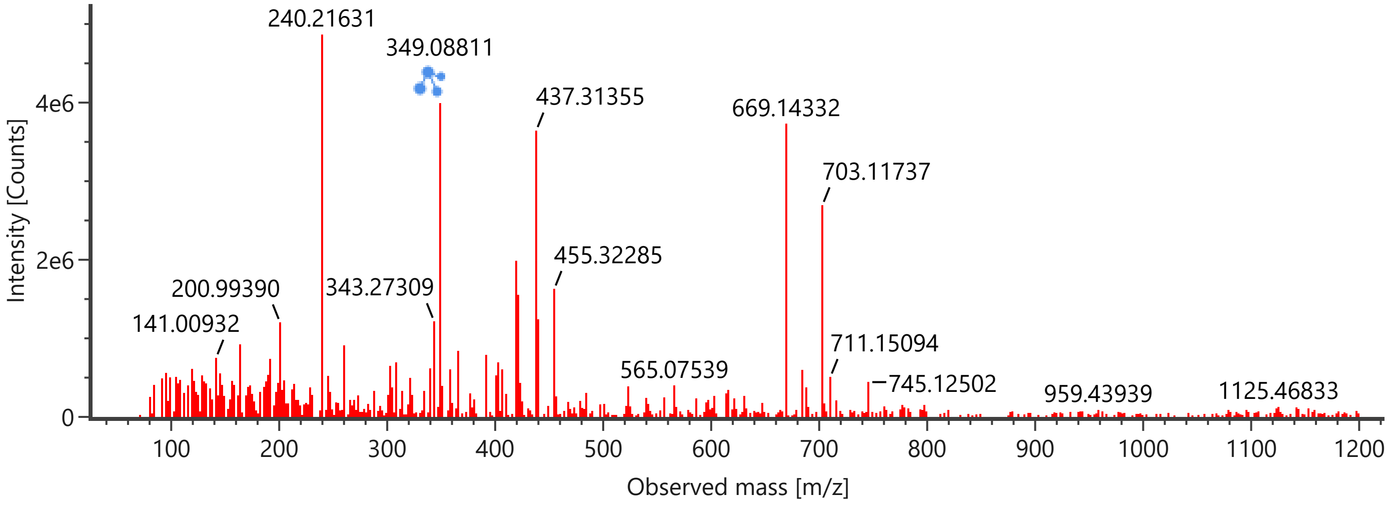

Created by: Administrator, UNIFI

Created on: May 08, 2026

Created time: 14:44:42 SE Asia Standard Time

Item name: La_VT_08052026

Component name: Eleutherazine B

Item name: La_VT_Ea_10042026
Channel name: Eleutherazine B [+H] : (45.5 PPM) 485.2601

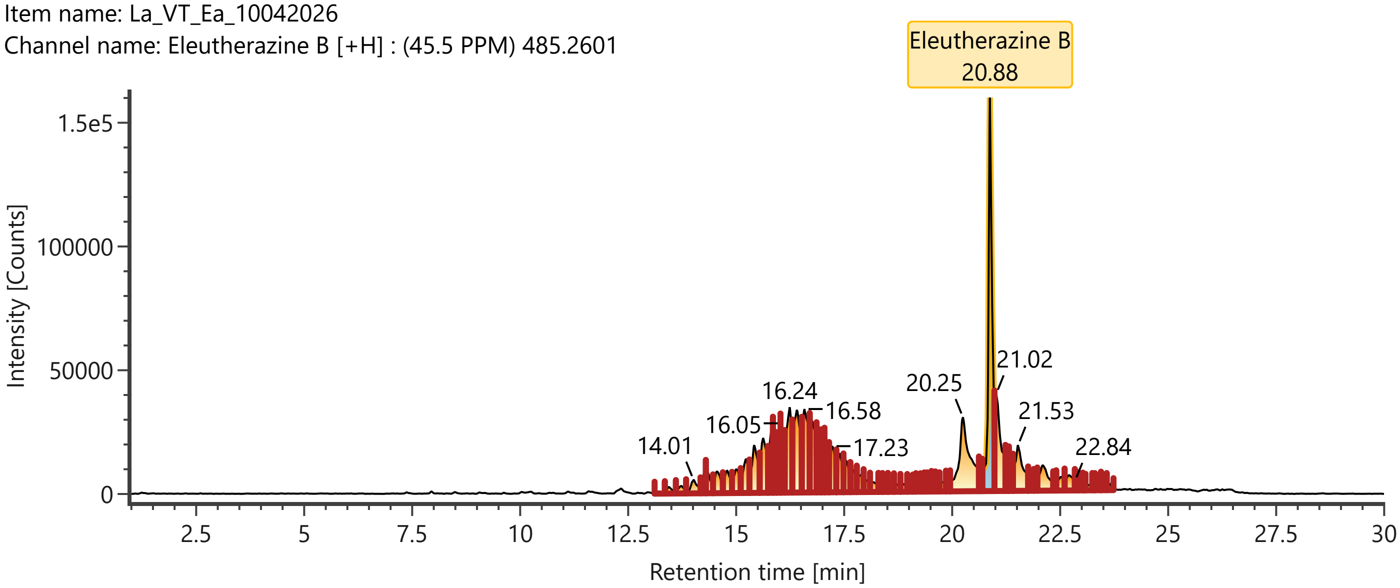

Item name: La_VT_Ea_10042026
Item description:

Channel name: Low energy : Time 20.8854 +/- 0.0525 minutes

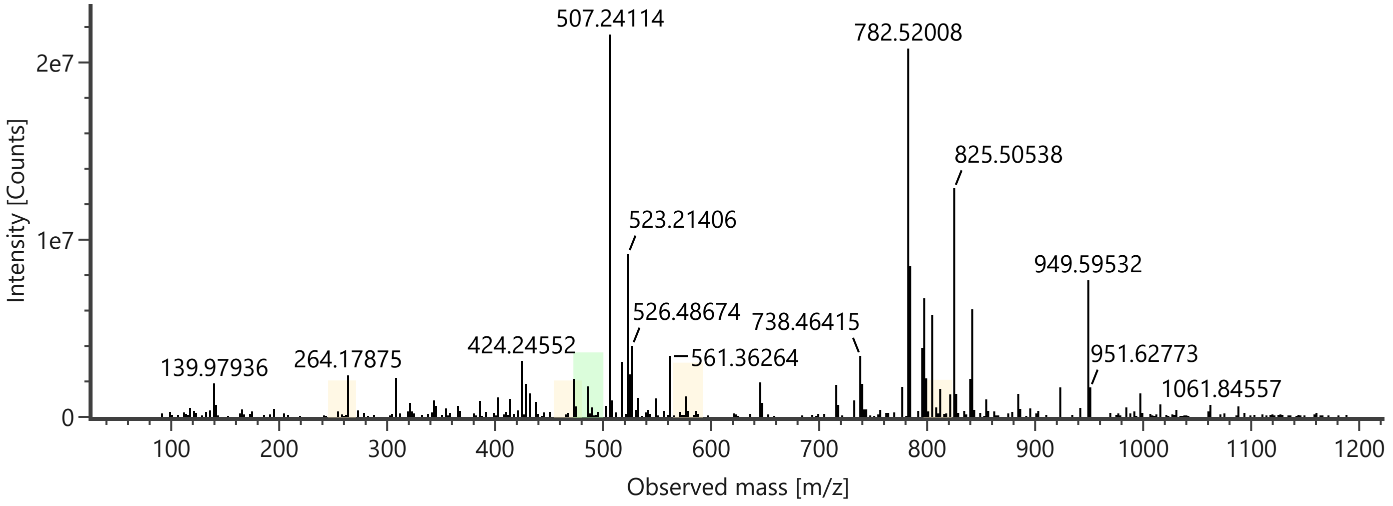

Item name: La_VT_Ea_10042026
Item description:

Channel name: High energy : Time 20.8854 +/- 0.0525 minutes

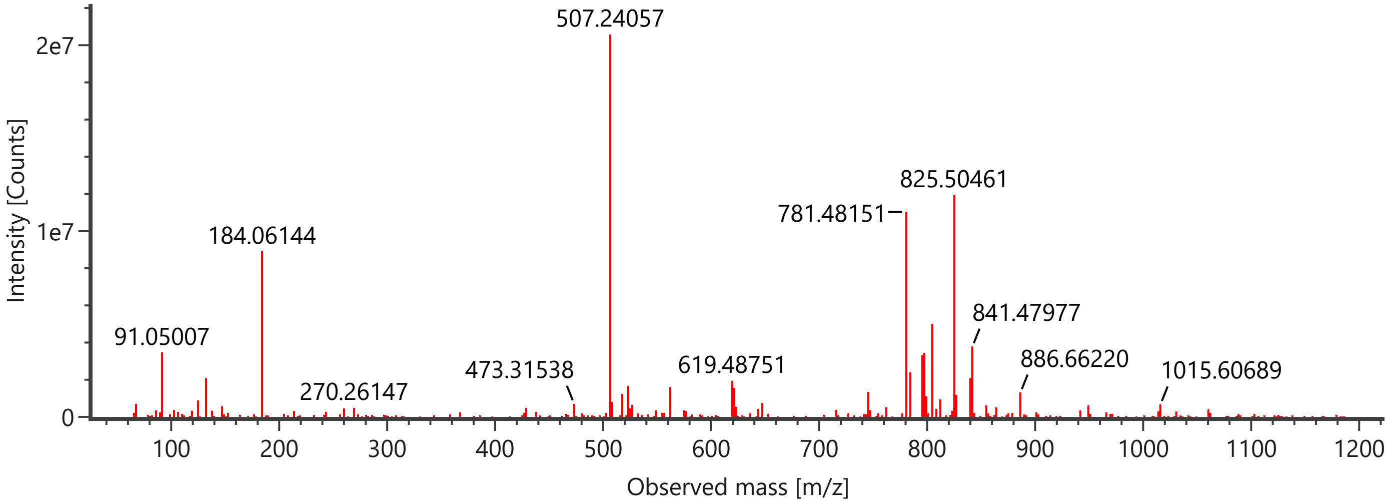

Item name: La_VT_08052026

Component name: Flavokawain B

Item name: La_VT_Ea_10042026
Channel name: Flavokawain B [+H] : (45.5 PPM) 285.1114

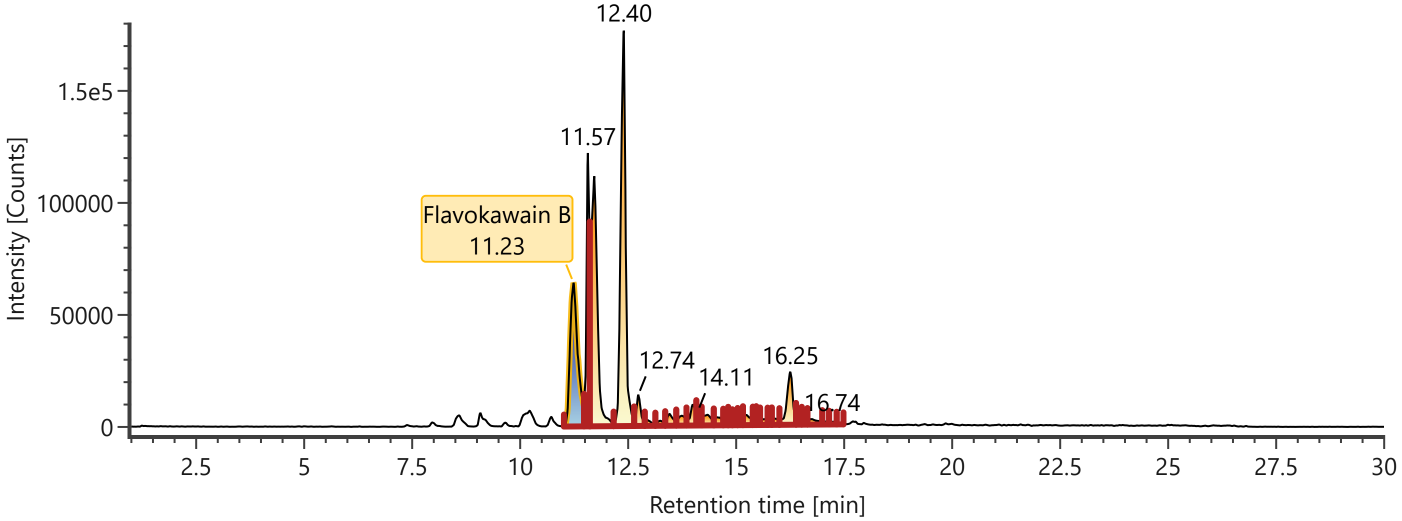

Item name: La_VT_Ea_10042026
Item description:

Channel name: Low energy : Time 11.2292 +/- 0.0525 minutes

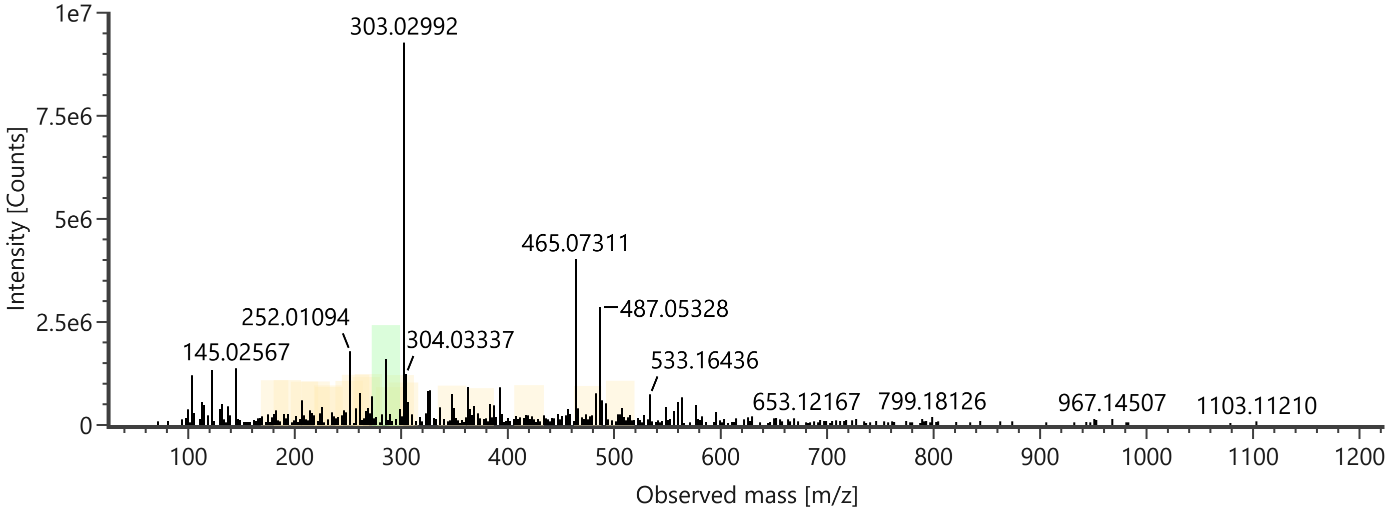

Item name: La_VT_Ea_10042026
Item description:

Channel name: High energy : Time 11.2292 +/- 0.0525 minutes

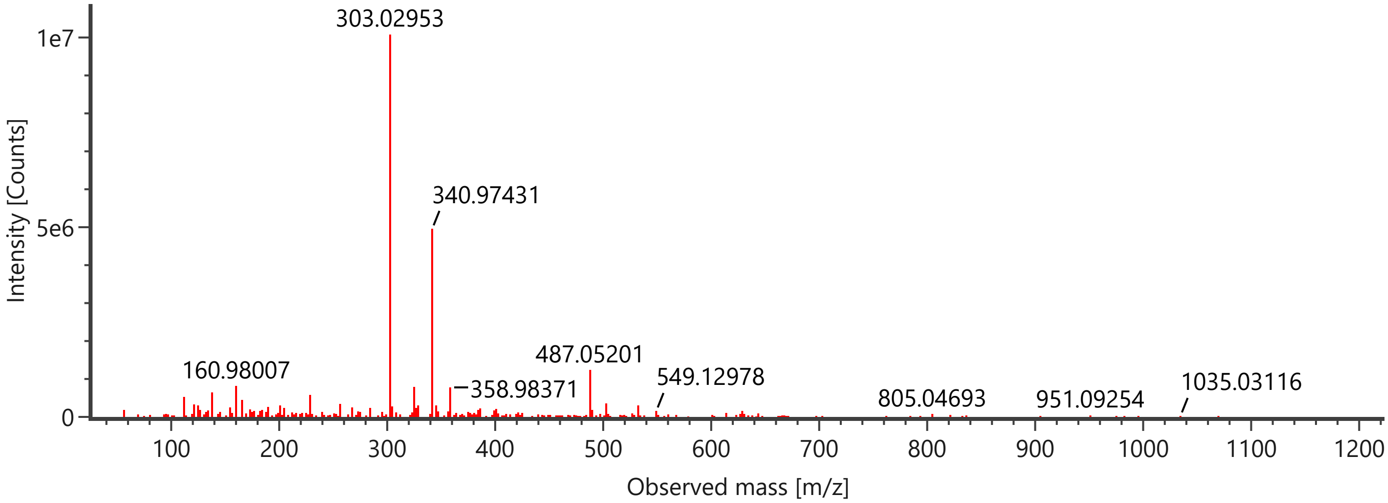

Created by: Administrator, UNIFI

Created on: May 08, 2026

Created time: 14:44:42 SE Asia Standard Time

Item name: La_VT_08052026

Component name: Glucosinalbin

Item name: La_VT_Ea_10042026
Channel name: Glucosinalbin [+H] : (45.5 PPM) 426.0539

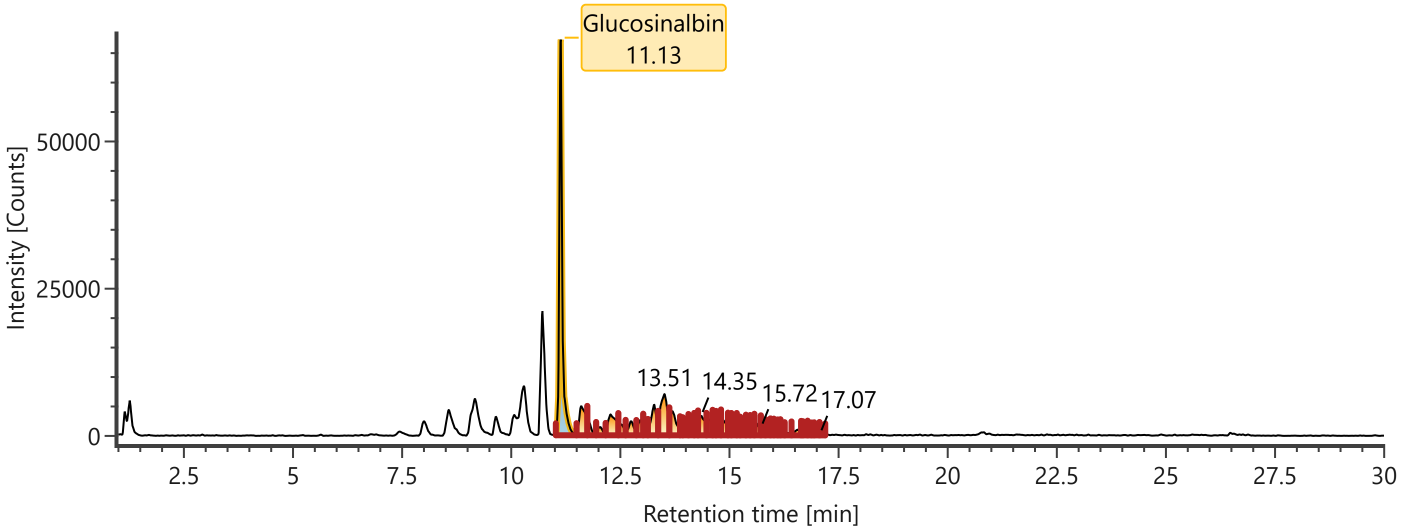

Item name: La_VT_Ea_10042026
Item description:

Channel name: Low energy : Time 11.1447 +/- 0.0525 minutes

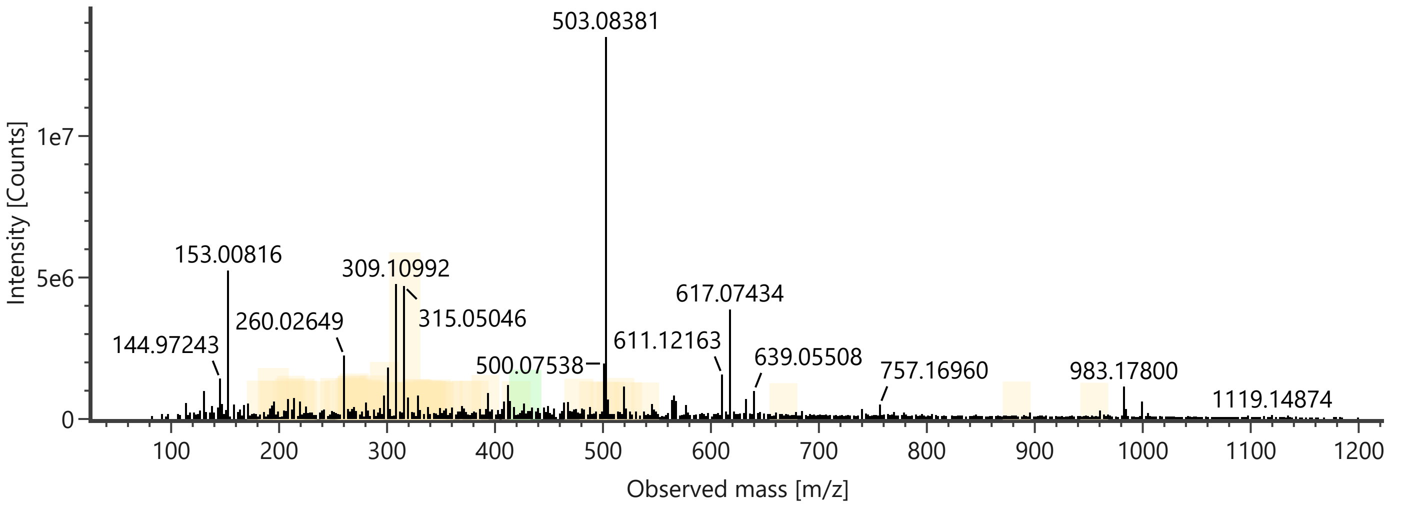

Item name: La_VT_Ea_10042026
Item description:

Channel name: High energy : Time 11.1447 +/- 0.0525 minutes

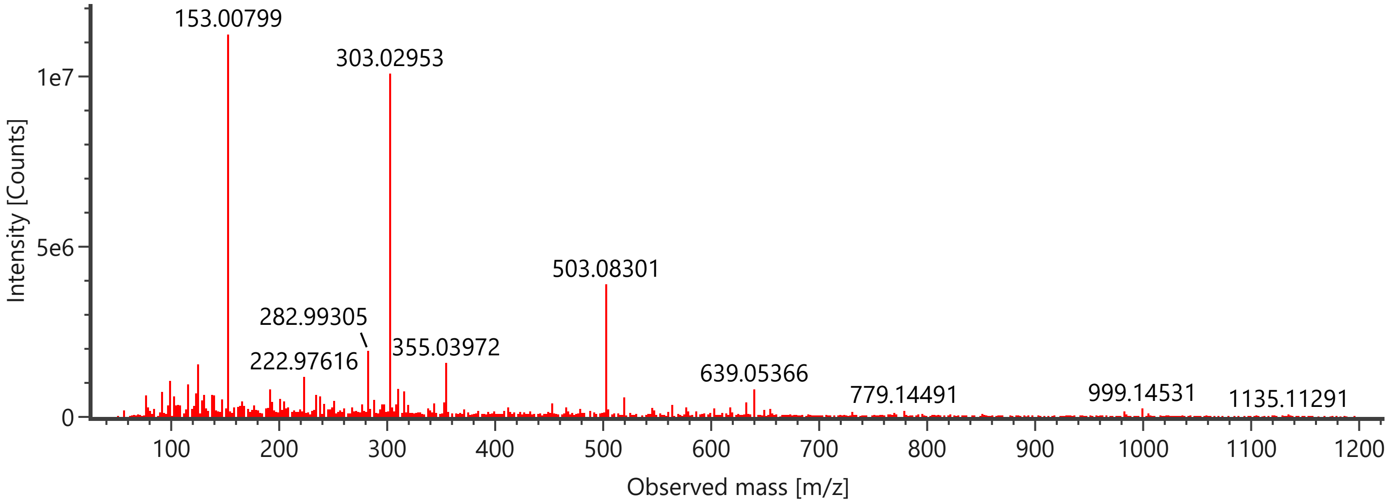

Created by: Administrator, UNIFI

Created on: May 08, 2026

Created time: 14:44:42 SE Asia Standard Time

Item name: La_VT_08052026

Component name: Hispidin

Item name: La_VT_Ea_10042026
Channel name: Hispidin [+H] : (45.5 PPM) 247.0612

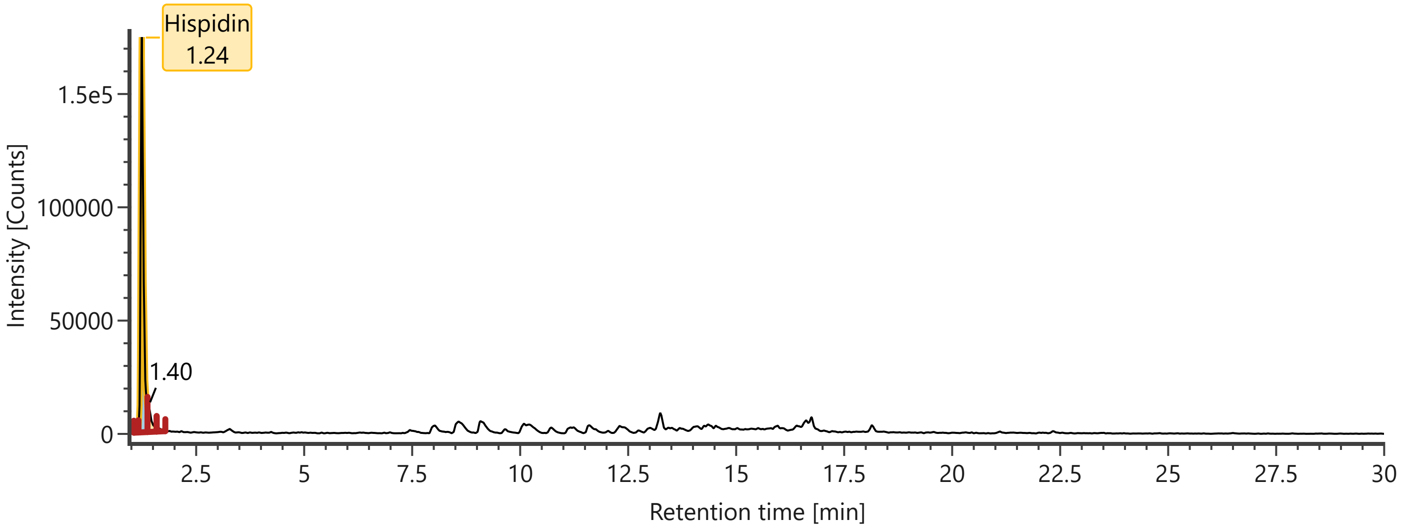

Item name: La_VT_Ea_10042026
Item description:

Channel name: Low energy : Time 1.2501 +/- 0.0525 minutes

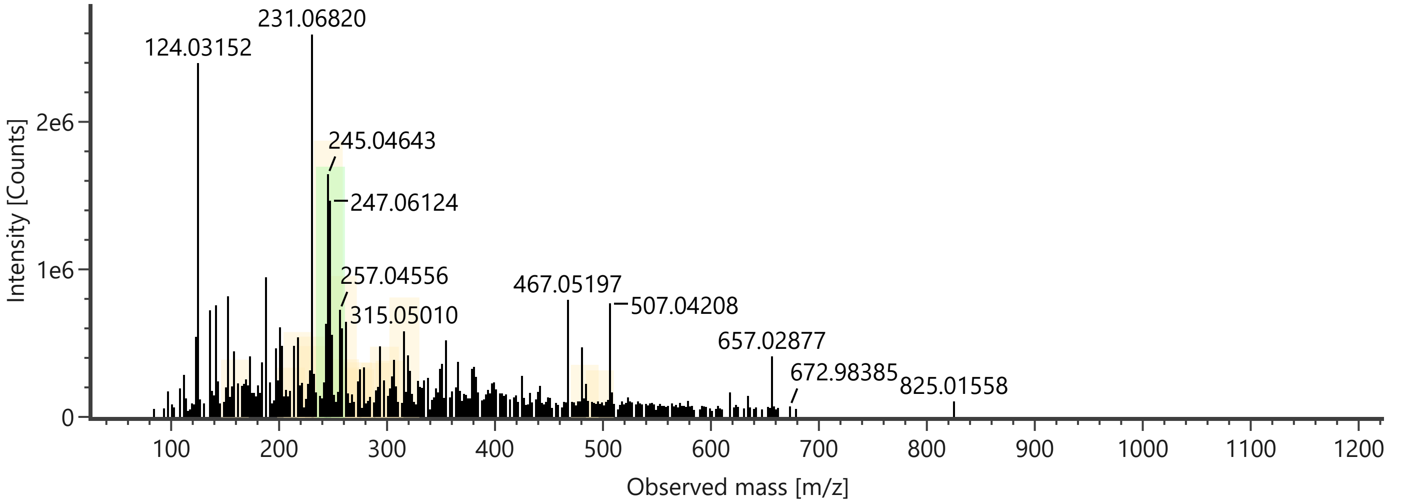

Item name: La_VT_Ea_10042026
Item description:

Channel name: High energy : Time 1.2501 +/- 0.0525 minutes

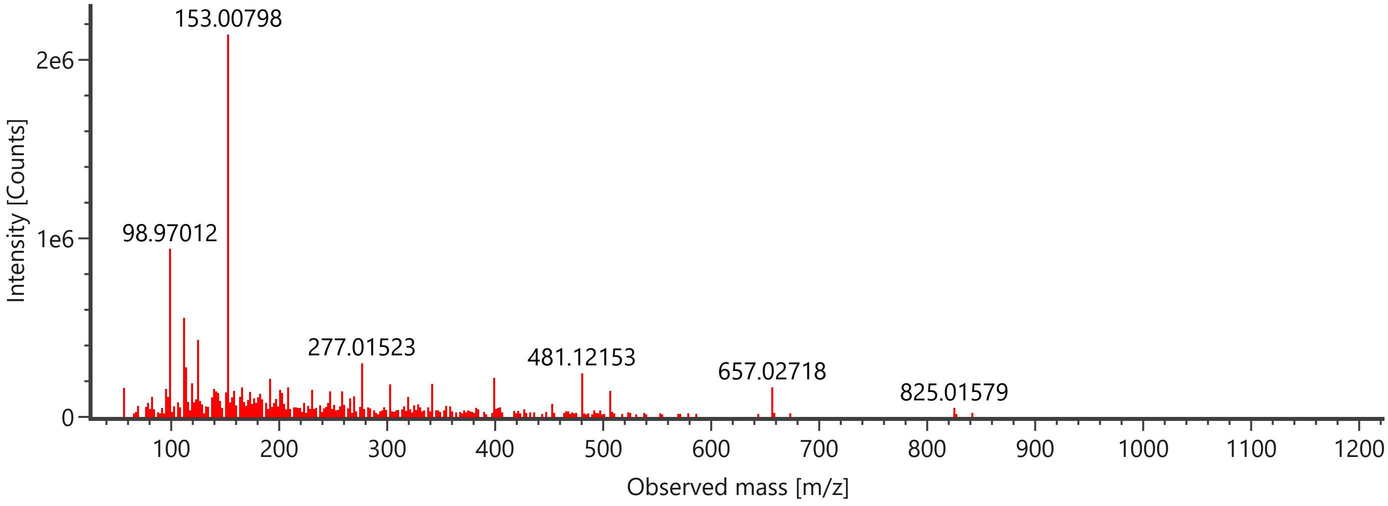

Item name: La_VT_08052026

Component name: Nuezhenidic acid

Item name: La_VT_Ea_10042026
Channel name: Nuezhenidic acid [+H] : (45.5 PPM) 453.1244

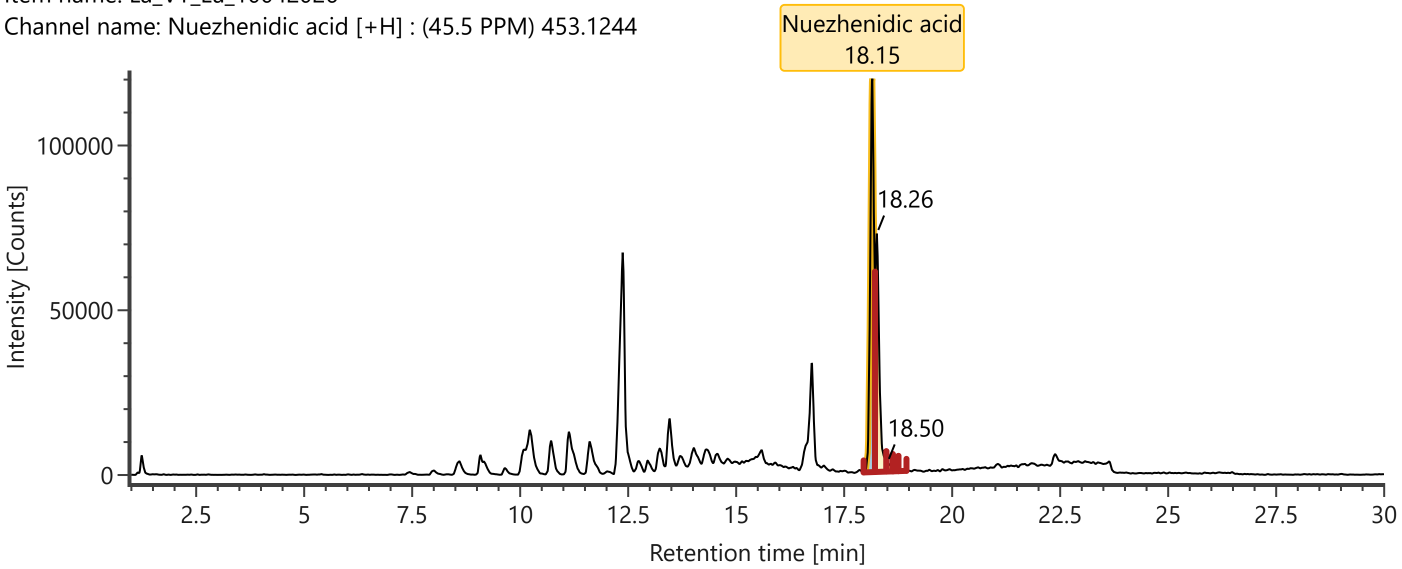

Item name: La_VT_Ea_10042026
Item description:
Channel name: Low energy : Time 18.1502 +/- 0.0525 minutes

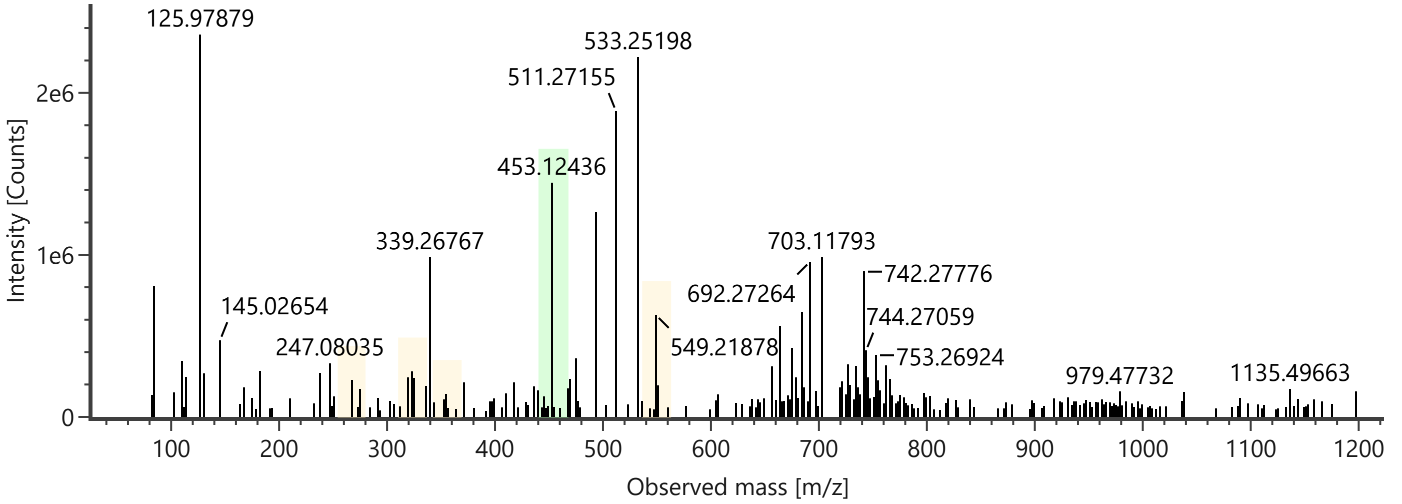

Item name: La_VT_Ea_10042026
Item description:

Channel name: High energy : Time 18.1502 +/- 0.0525 minutes

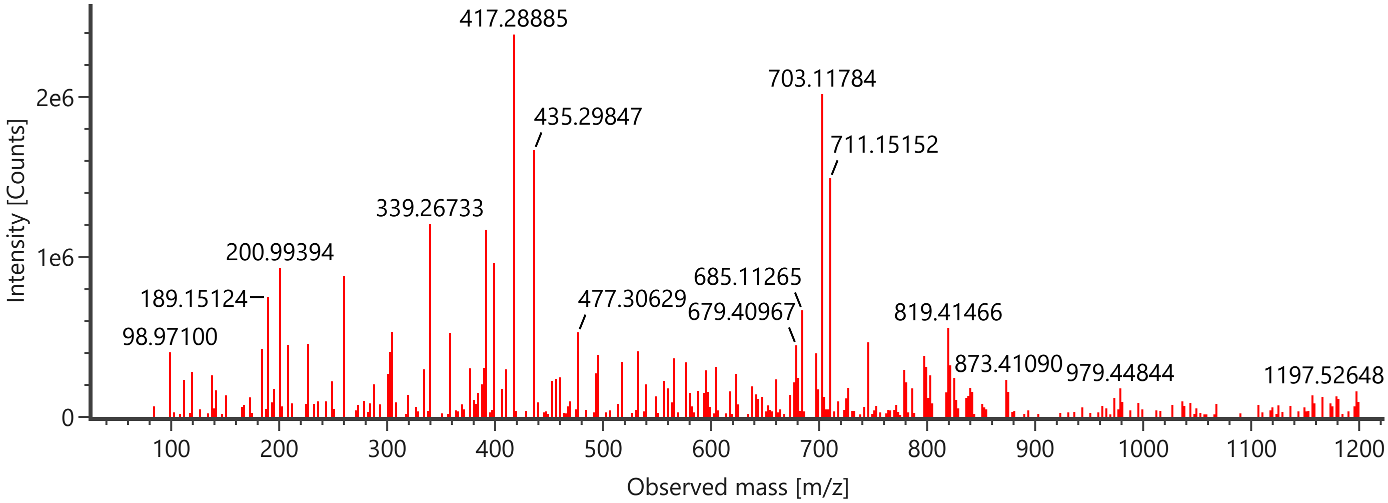

Item name: La_VT_08052026

Component name: Periplocoside C

Item name: La_VT_Ea_10042026
Channel name: Periplocoside C [+H] : (45.5 PPM) 921.5186

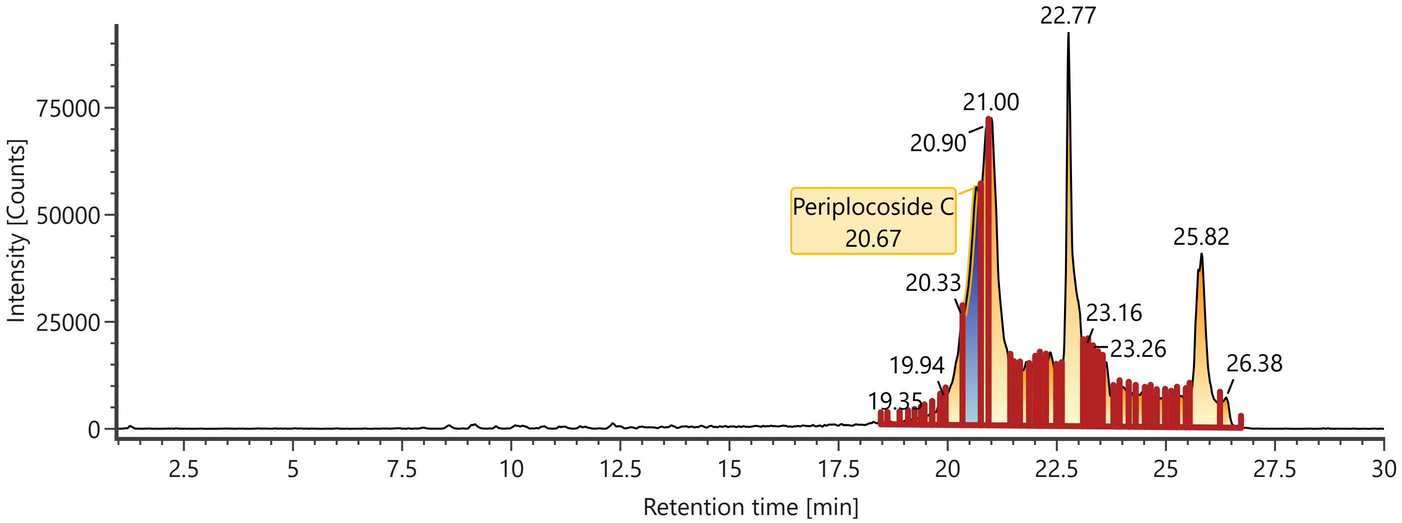

Item name: La_VT_Ea_10042026
Item description:
Channel name: Low energy : Time 20.6508 +/- 0.0525 minutes

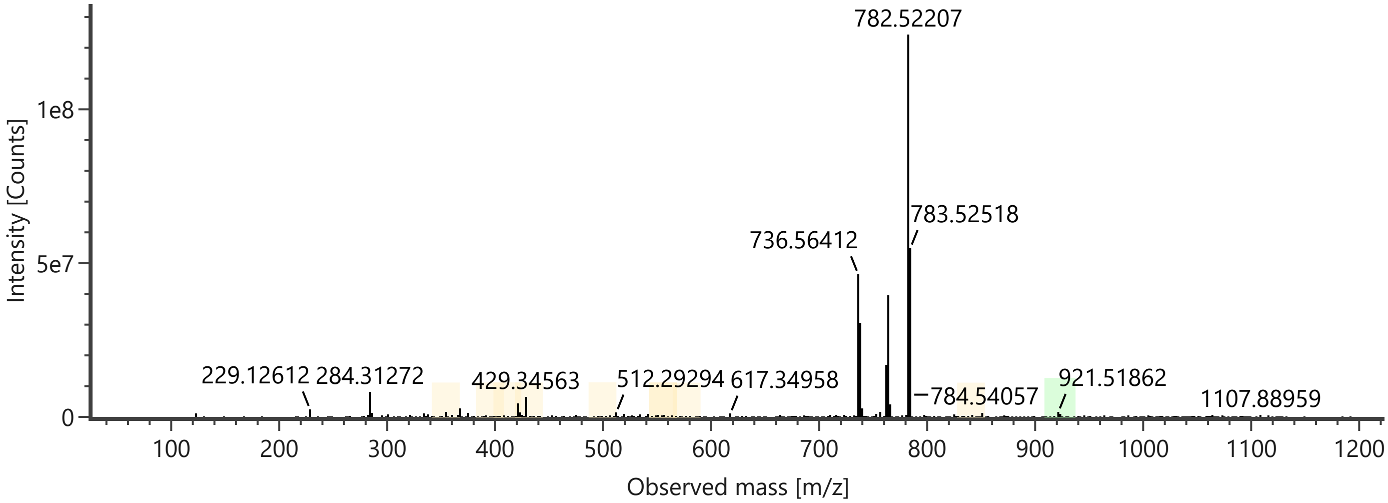

Item name: La_VT_08052026

Created by: Administrator, UNIFI

Created on: May 08, 2026

Created time: 14:44:42 SE Asia Standard Time

Item name: La_VT_Ea_10042026
Item description:

Channel name: High energy : Time 20.6508 +/- 0.0525 minutes

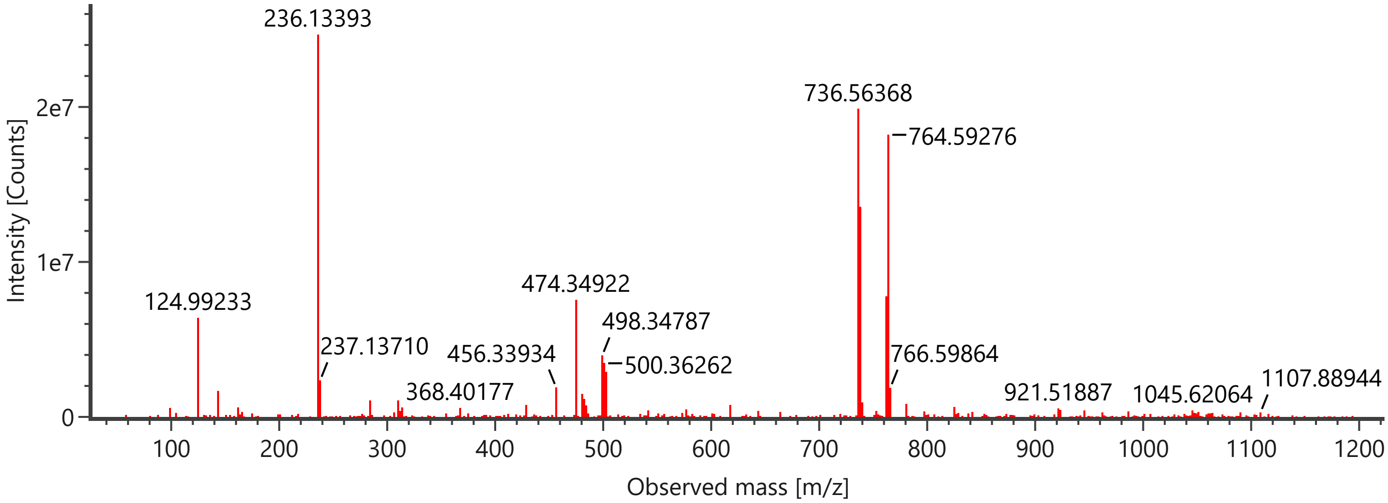

Item name: La_VT_08052026

Created by: Administrator, UNIFI

Created on: May 08, 2026

Created time: 14:44:42 SE Asia Standard Time

Component name: Periplocoside M

Item name: La_VT_Ea_10042026
Channel name: Periplocoside M [+H] : (45.5 PPM) 605.3687

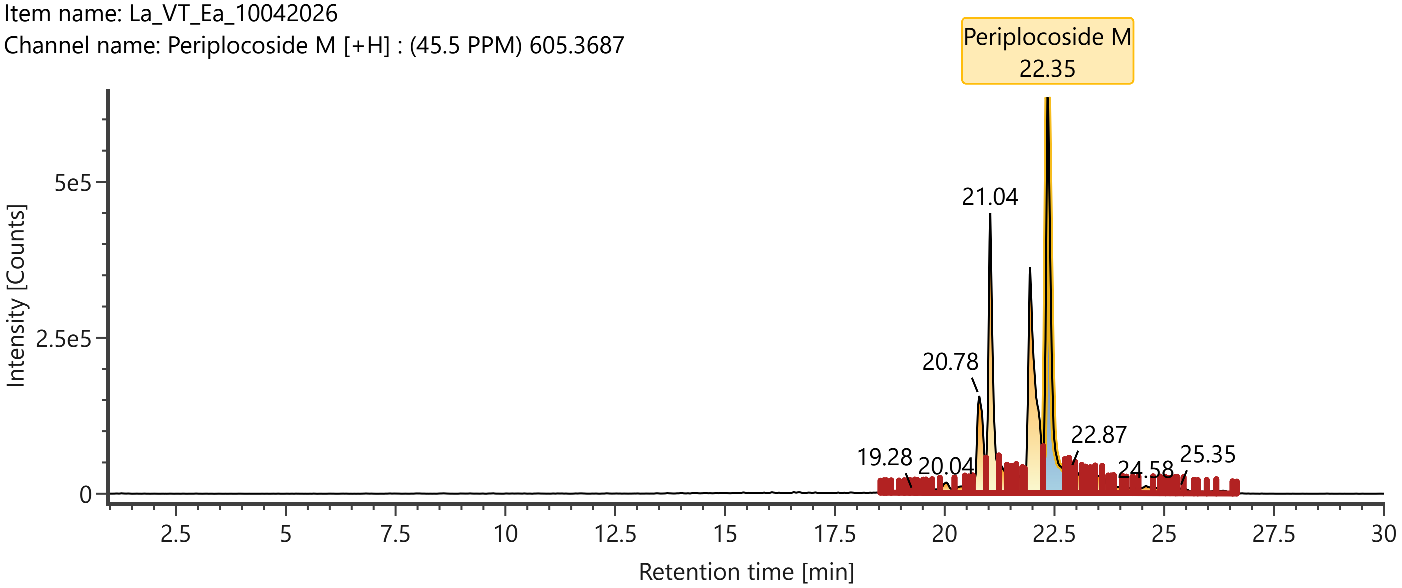

Item name: La_VT_Ea_10042026
Item description:

Channel name: Low energy : Time 22.3571 +/- 0.0525 minutes

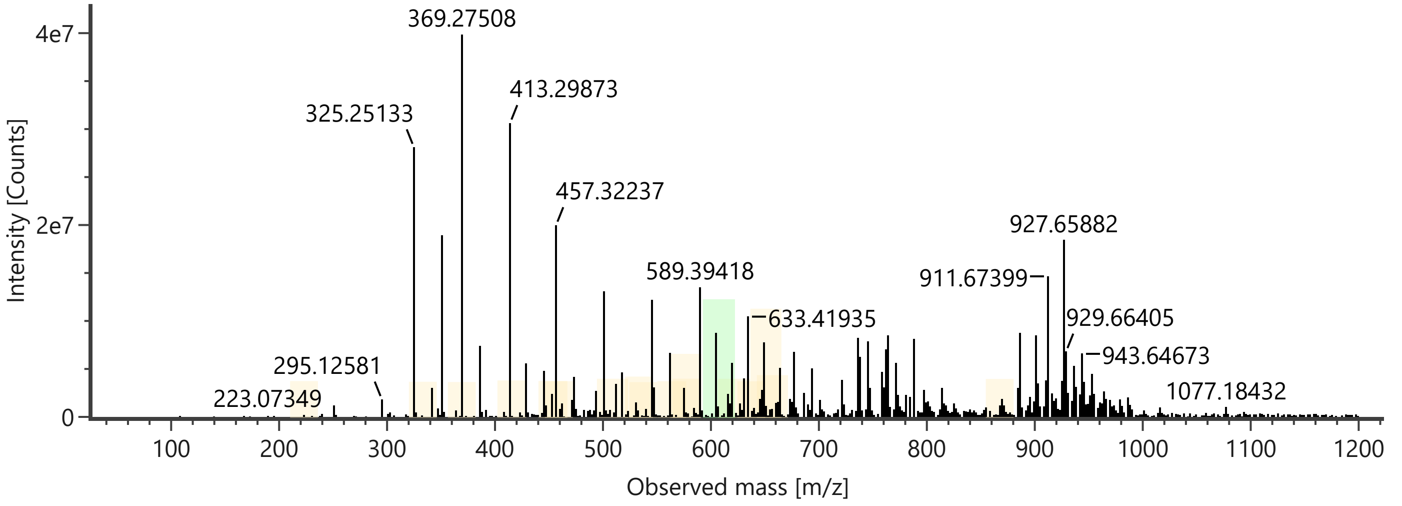

Item name: La_VT_08052026

Created by: Administrator, UNIFI

Created on: May 08, 2026

Created time: 14:44:42 SE Asia Standard Time

Item name: La_VT_Ea_10042026
Item description:

Channel name: High energy : Time 22.3571 +/- 0.0525 minutes

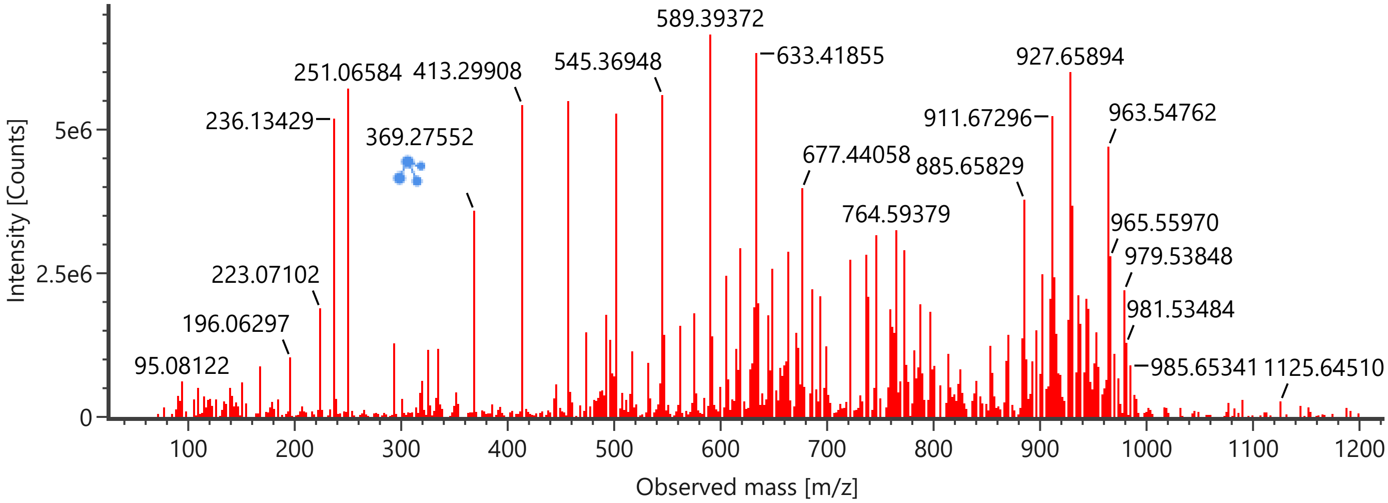

Item name: La_VT_08052026

Created by: Administrator, UNIFI

Created on: May 08, 2026

Created time: 14:44:42 SE Asia Standard Time

Component name: Phytolaccagenin

Item name: La_VT_Ea_10042026
Channel name: Phytolaccagenin [+H] : (45.5 PPM) 533.3479

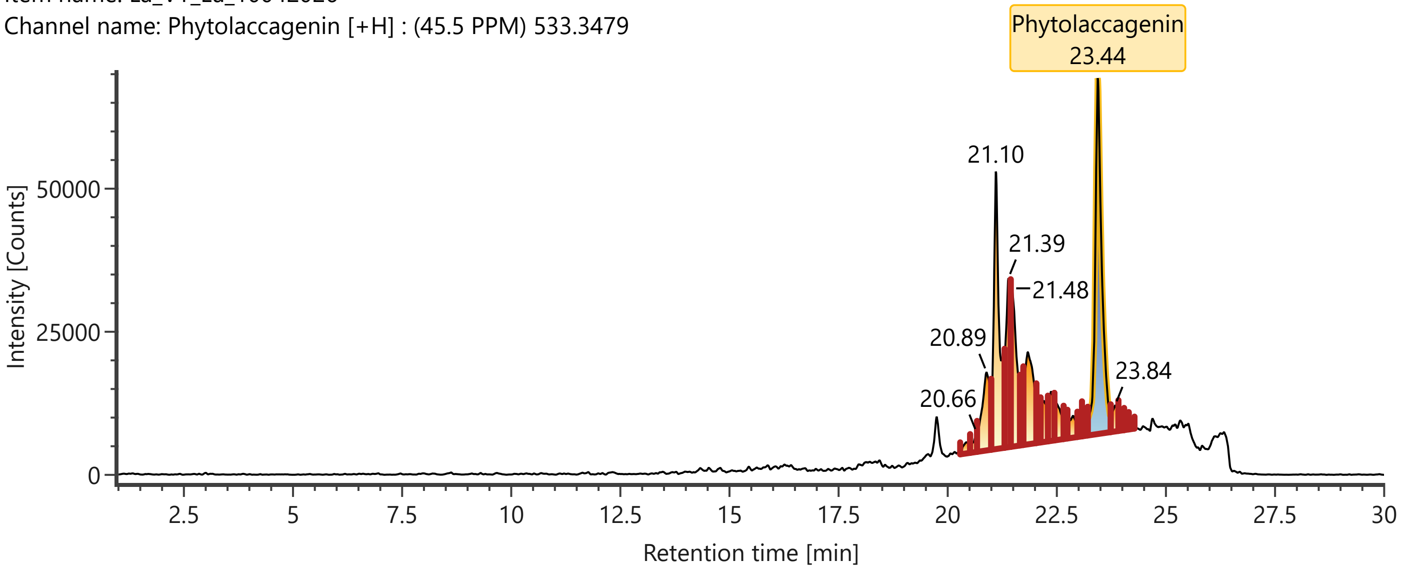

Item name: La_VT_Ea_10042026
Item description:

Channel name: Low energy : Time 23.4467 +/- 0.0525 minutes

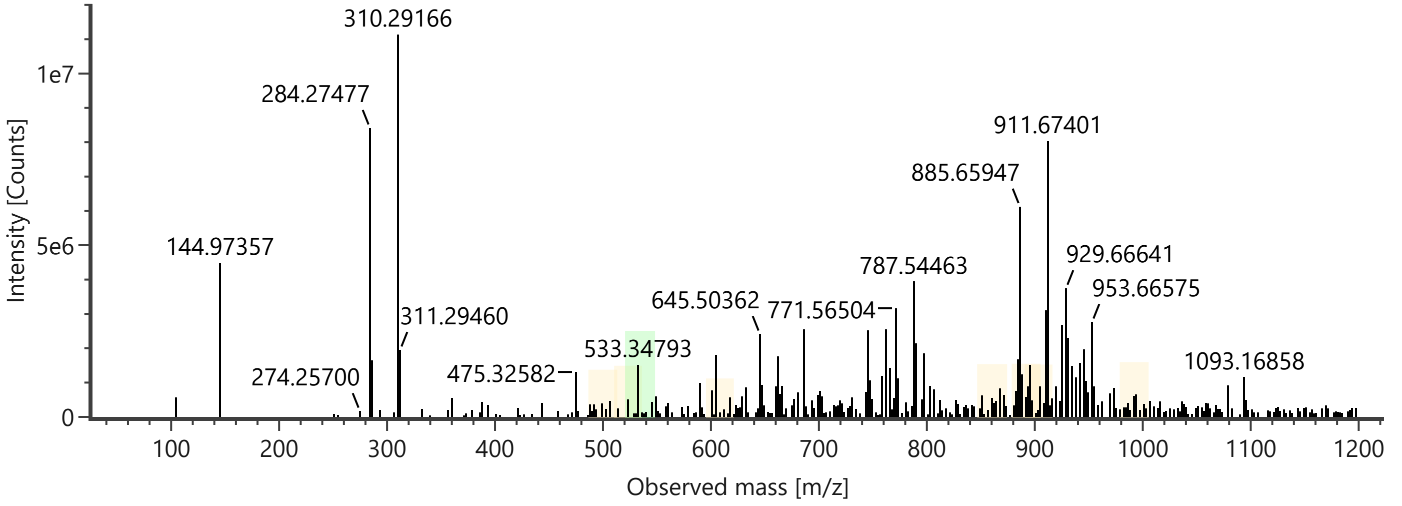

Item name: La_VT_Ea_10042026
Item description:

Channel name: High energy : Time 23.4467 +/- 0.0525 minutes

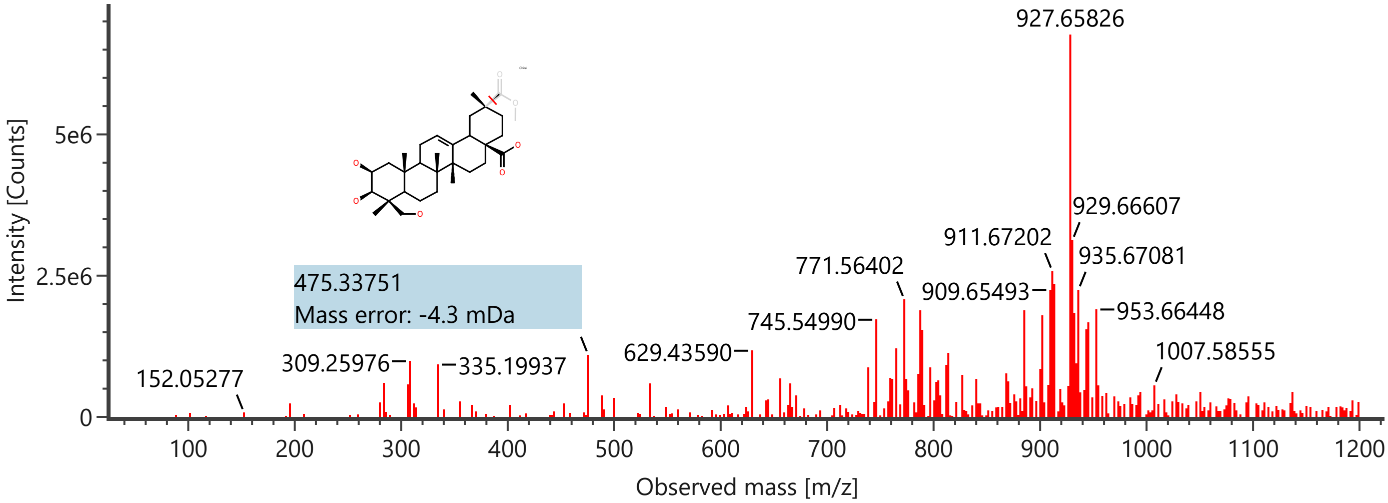

Item name: La_VT_08052026

Component name: Toosendanin_1

Item name: La_VT_Ea_10042026
Channel name: Toosendanin_1 [+H] : (45.5 PPM) 575.2485

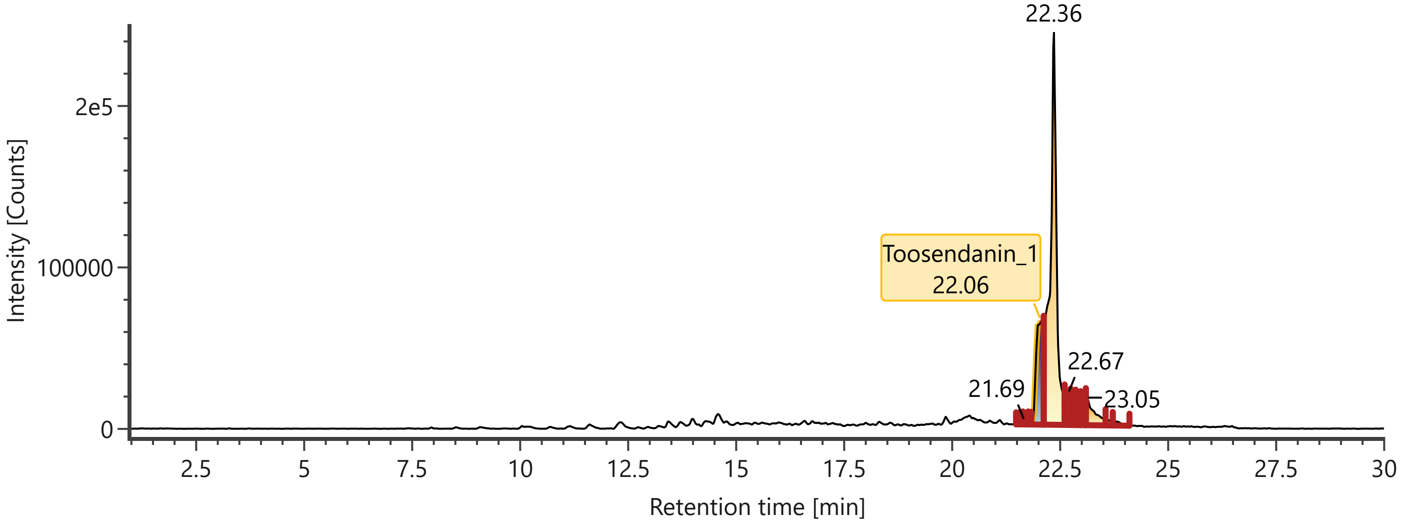

Item name: La_VT_Ea_10042026
Item description:
Channel name: Low energy : Time 22.0109 +/- 0.0525 minutes

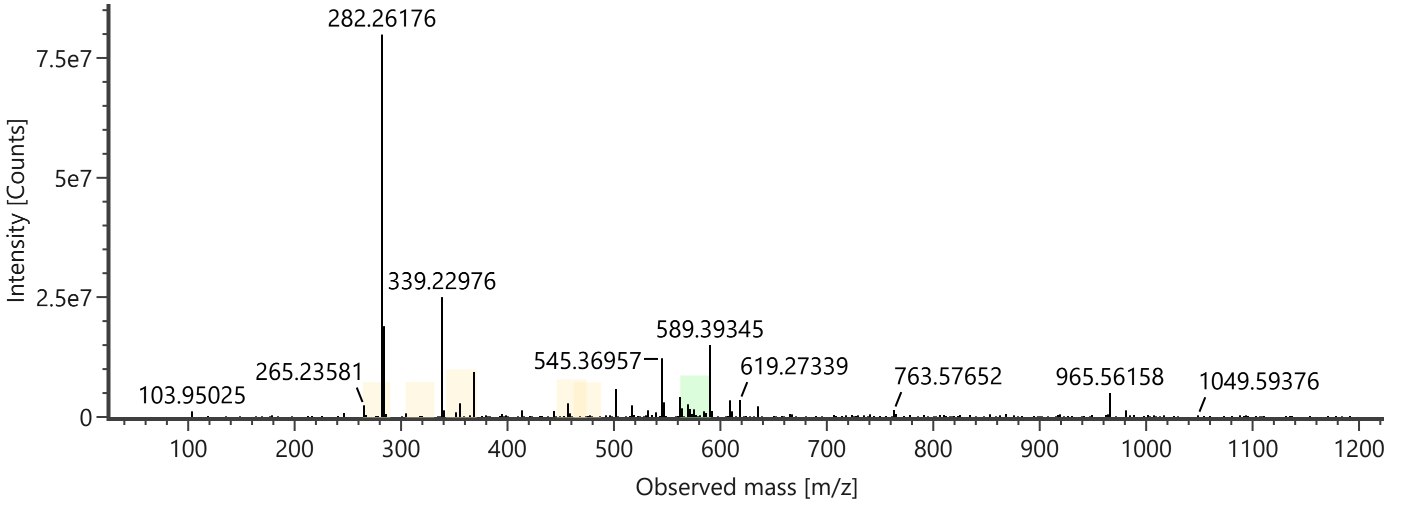

Created by: Administrator, UNIFI

Created on: May 08, 2026

Created time: 14:44:42 SE Asia Standard Time

Item name: La_VT_08052026

Item name: La_VT_Ea_10042026

Item description:

Channel name: High energy : Time 22.0109 +/- 0.0525 minutes

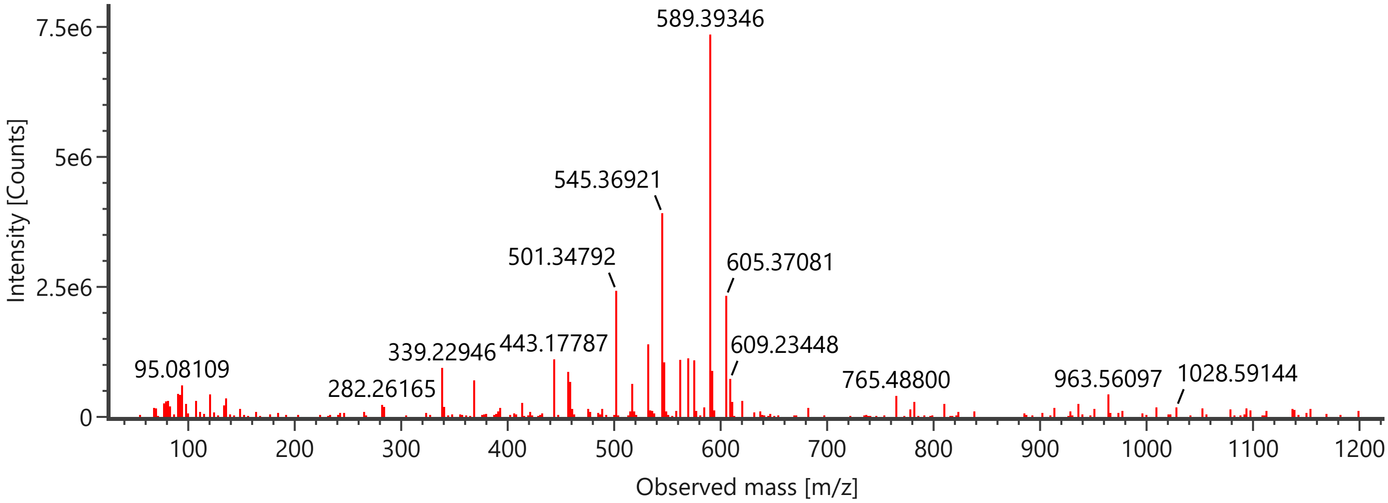

Item name: La_VT_08052026

Created by: Administrator, UNIFI

Created on: May 08, 2026

Created time: 14:44:42 SE Asia Standard Time

Component name: Toosendanin_1

Item name: La_VT_Ea_10042026
Channel name: Toosendanin_1 [+H] : (45.5 PPM) 575.2497

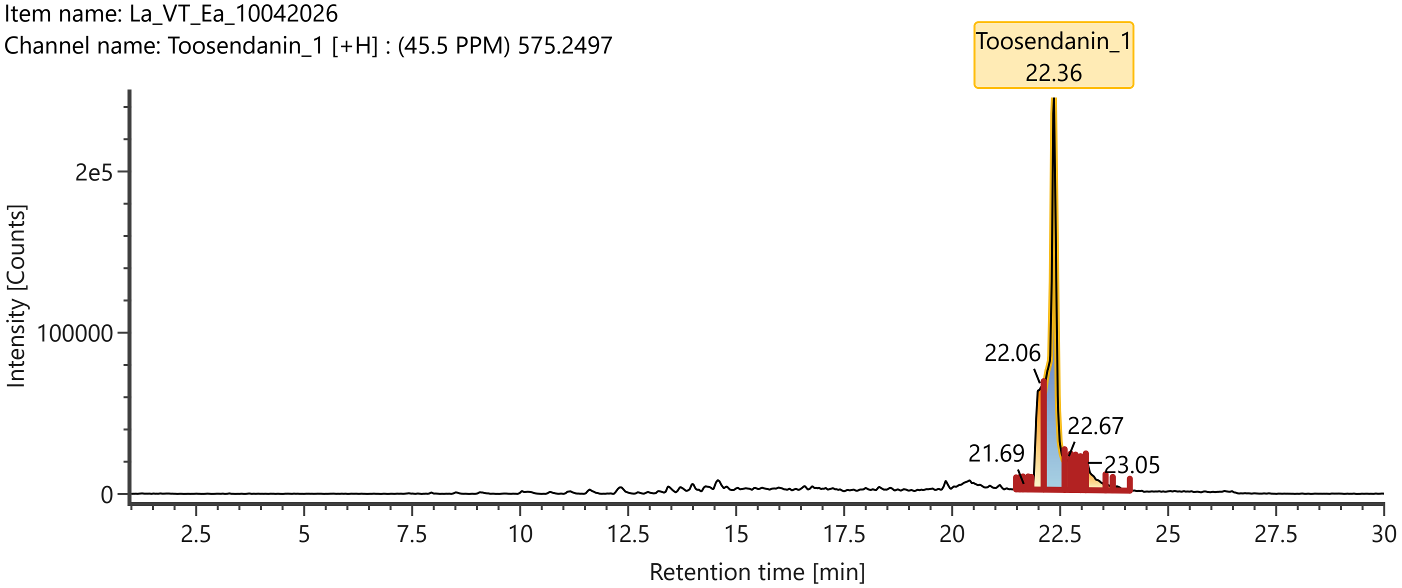

Item name: La_VT_Ea_10042026
Item description:

Channel name: Low energy : Time 22.3592 +/- 0.0525 minutes

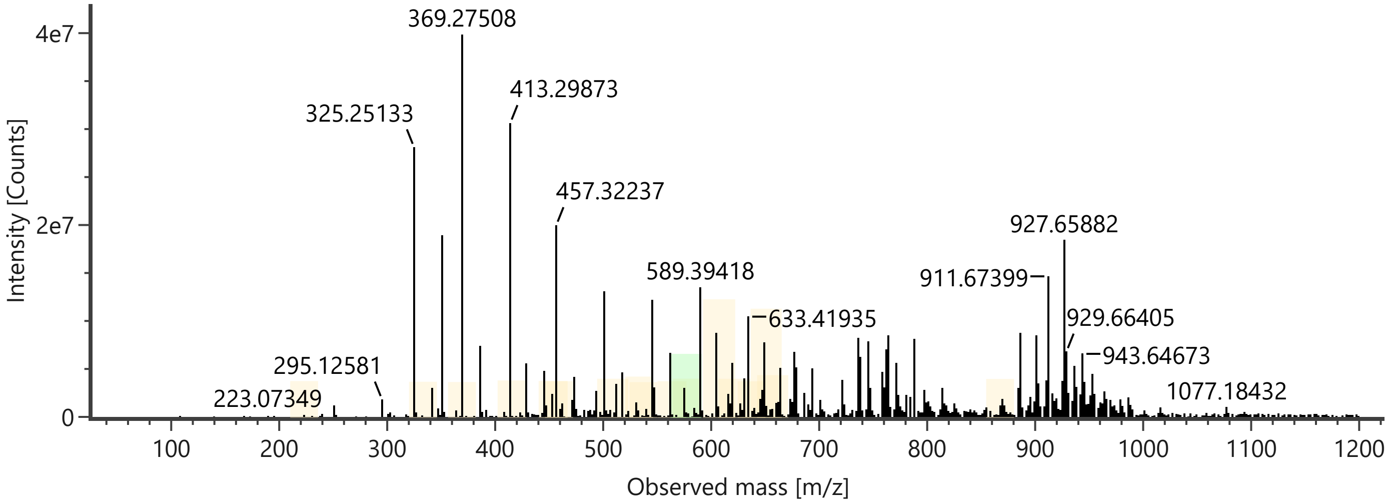

Item name: La_VT_Ea_10042026
Item description:

Channel name: High energy : Time 22.3592 +/- 0.0525 minutes

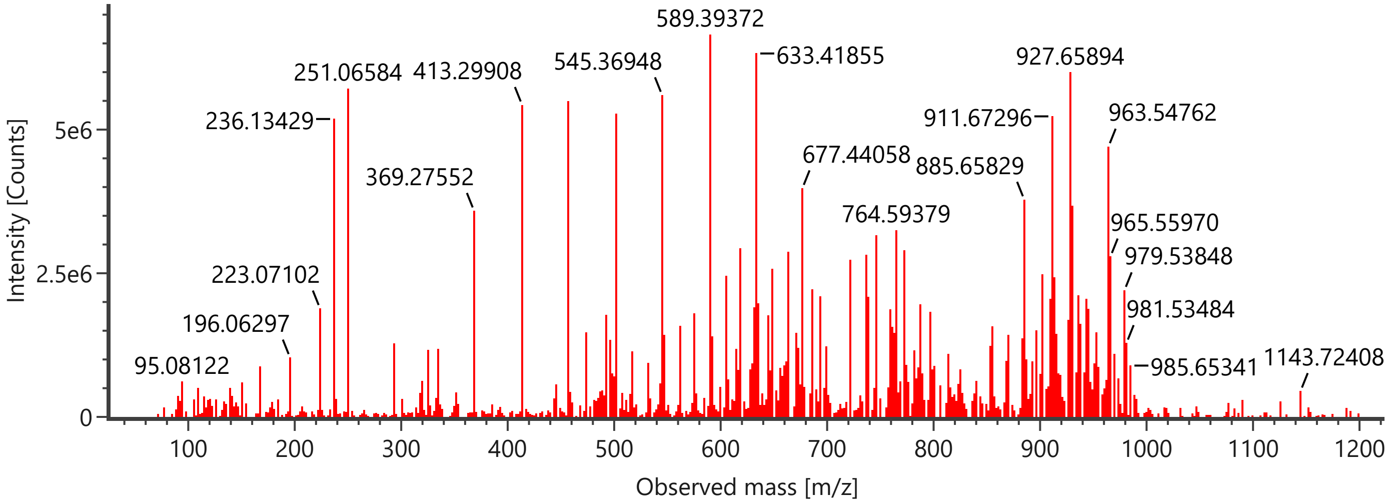

Item name: La_VT_08052026

Component name: Yadanzioside A

Item name: La_VT_Ea_10042026
Channel name: Yadanzioside A [+H] : (45.5 PPM) 685.2683

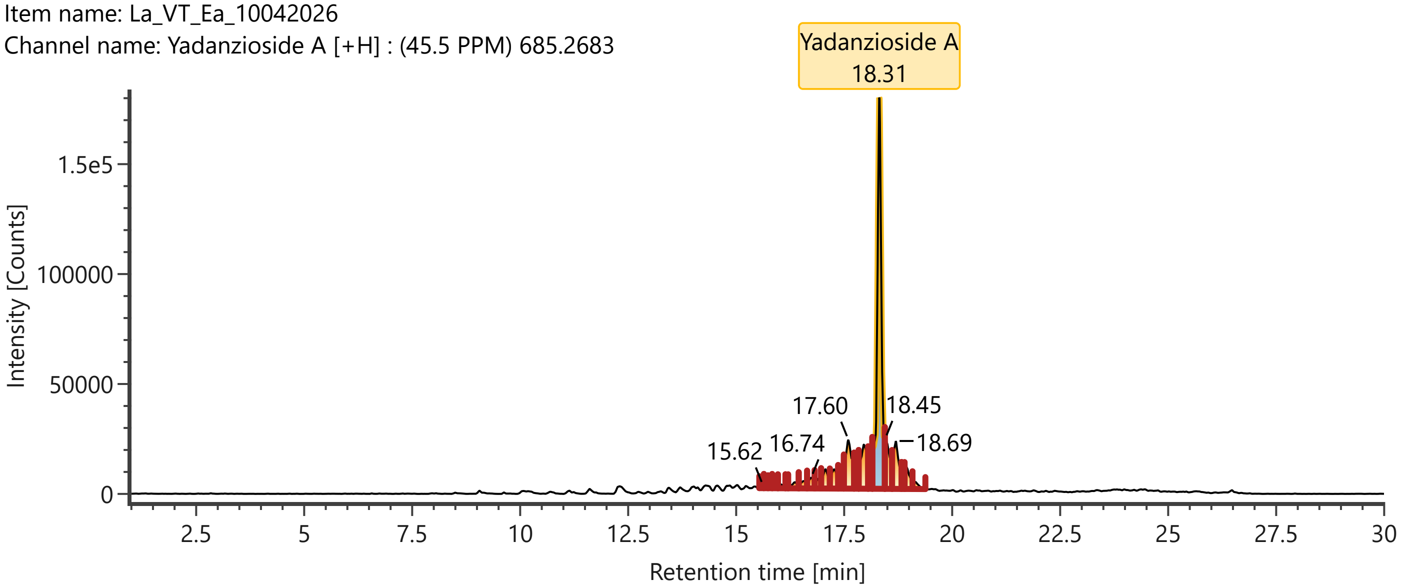

Item name: La_VT_Ea_10042026
Item description:

Channel name: Low energy : Time 18.3174 +/- 0.0525 minutes

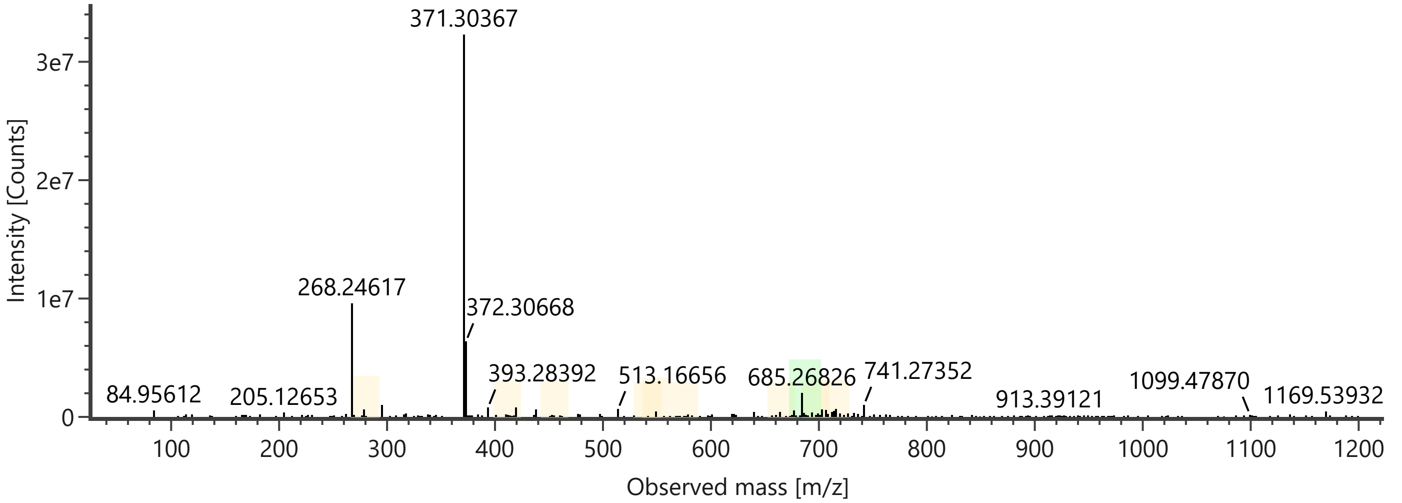

Item name: La_VT_Ea_10042026
Item description:

Channel name: High energy : Time 18.3174 +/- 0.0525 minutes

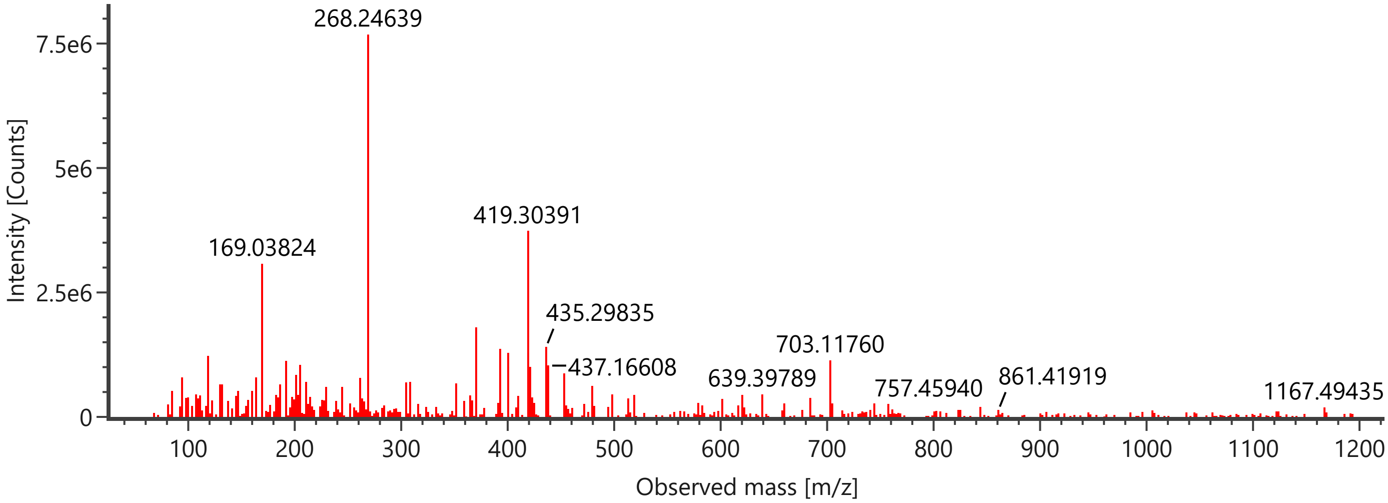

Item name: La_VT_08052026

Component name: Yemuoside YM6

Item name: La_VT_Ea_10042026
Channel name: Yemuoside YM6 [+H] : (45.5 PPM) 671.2541

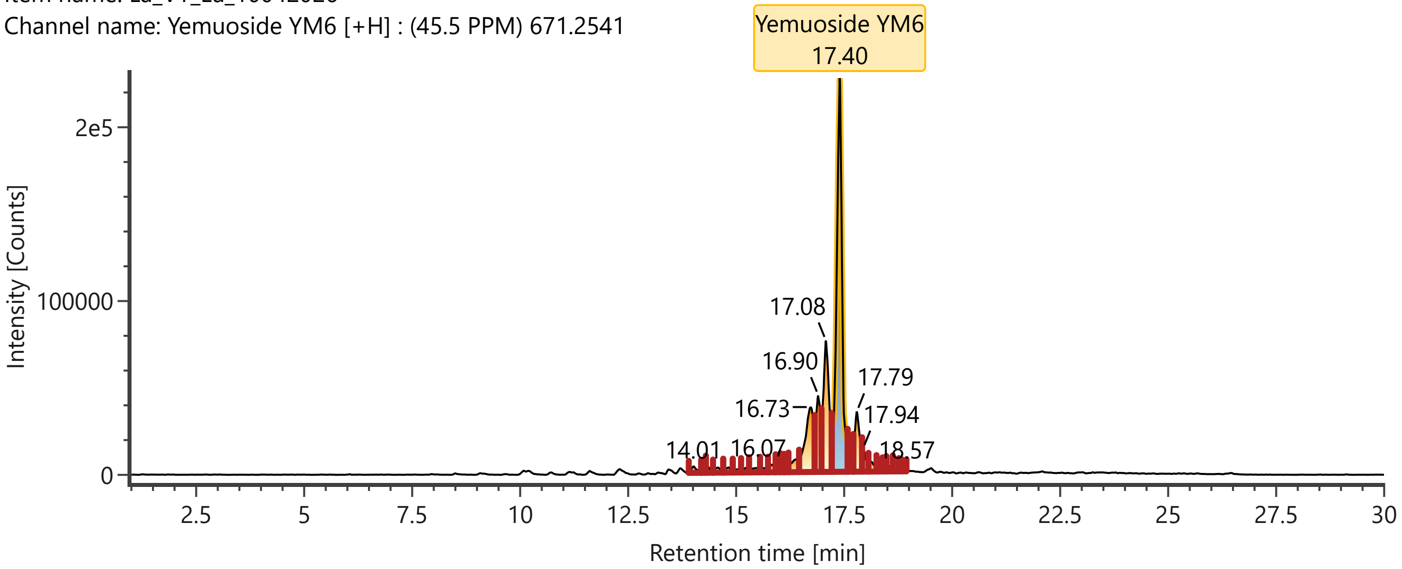

Item name: La_VT_Ea_10042026
Item description:
Channel name: Low energy : Time 17.3929 +/- 0.0525 minutes

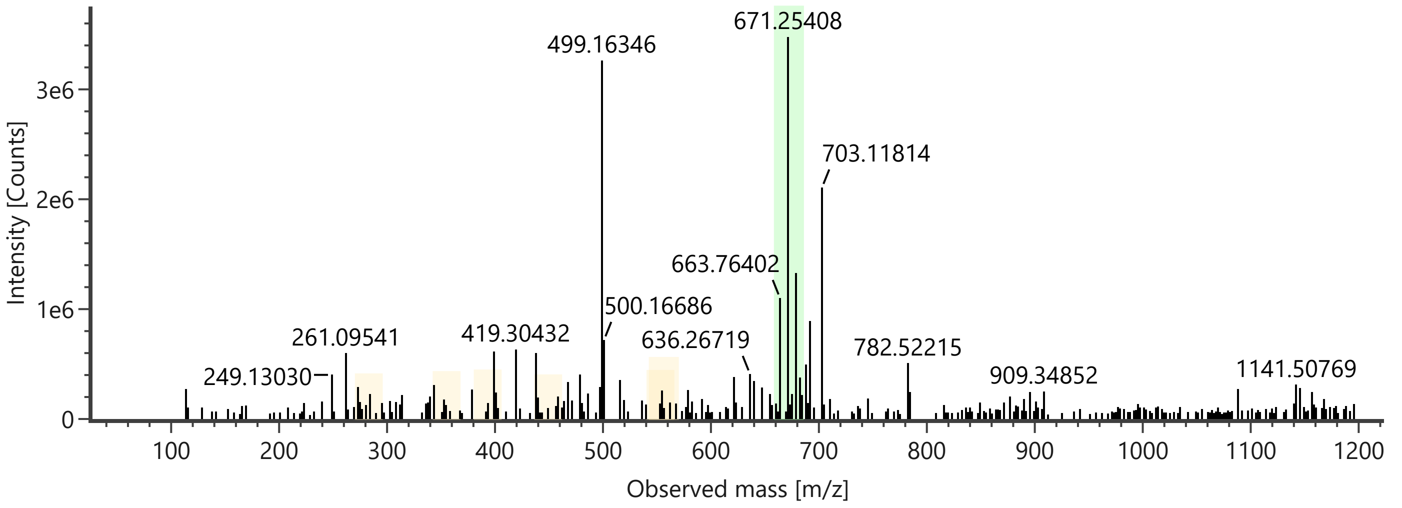

Item name: La_VT_Ea_10042026
Item description:

Channel name: High energy : Time 17.3929 +/- 0.0525 minutes

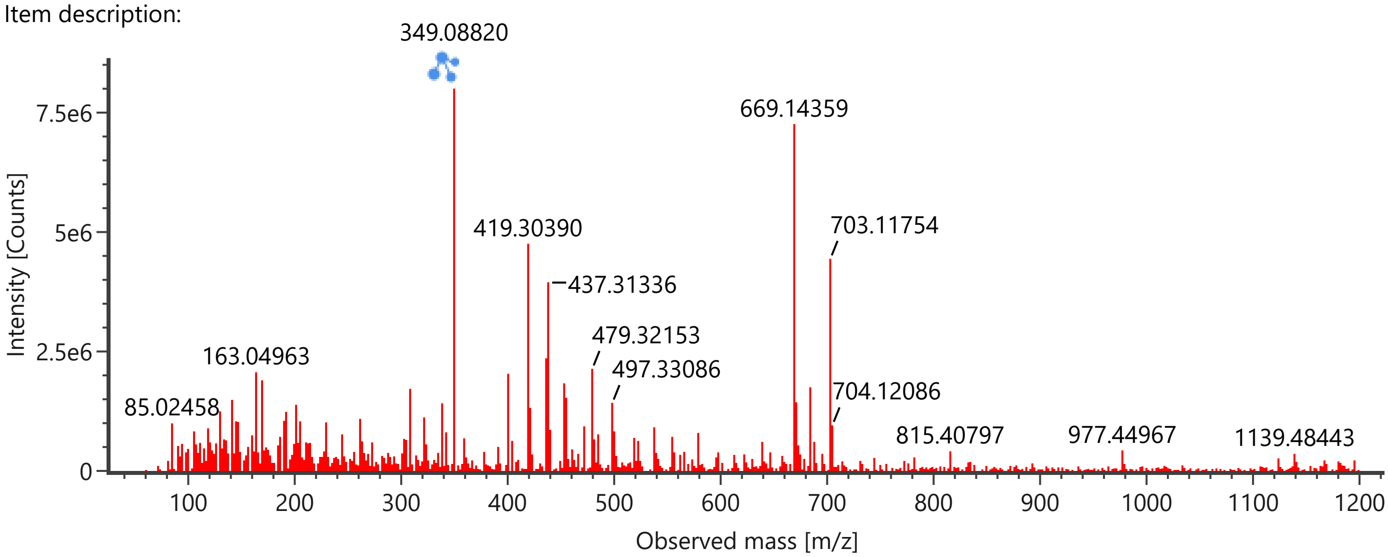

Report Log

Template Report

Report Template "Untitled" has not been saved.
